# Glycosyl-Nucleolipids as New Bioinspired Amphiphiles

**DOI:** 10.3390/molecules181012241

**Published:** 2013-09-30

**Authors:** Laurent Latxague, Amit Patwa, Eric Amigues, Philippe Barthélémy

**Affiliations:** 1 INSERM U869, Bordeaux, F-33076, France; 2 Université de Bordeaux, Bordeaux, F-33076, France

**Keywords:** nucleolipid, nucleoside, glycosyl, fluorocarbon, amphiphiles, surface tension

## Abstract

Four new Glycosyl-NucleoLipid (GNL) analogs featuring either a single fluorocarbon or double hydrocarbon chains were synthesized in good yields from azido thymidine as starting material. Physicochemical studies (surface tension measurements, differential scanning calorimetry) indicate that hydroxybutanamide-based GNLs feature endothermic phase transition temperatures like the previously reported double chain glycerol-based GNLs. The second generation of GNFs featuring a free nucleobase reported here presents a better surface activity (lower γ_lim_) compared to the first generation of GNFs.

## 1. Introduction

**Figure 1:**
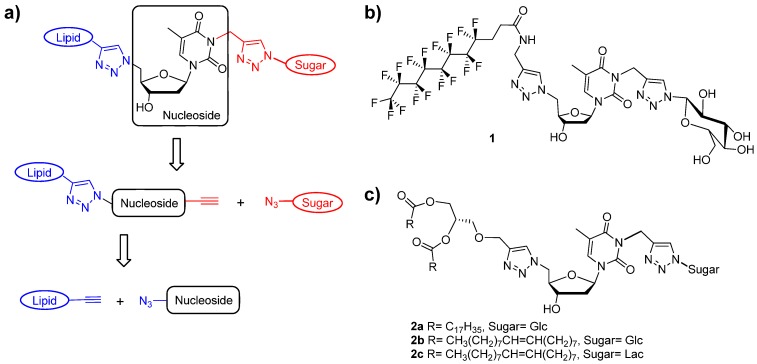
(**a**) Retrosynthetic pathway leading to the GNLs in to steps, (**b**) Fluorinated GNL **1** also designed by the acronym GNF (Glycosyl-nucleoside fluorinated amphiphile). (**c**) Double chain GNLs based on a glycerol backbone.

The chemical combination of biological molecules such as nucleic acids [1,2,3], aminoacids [4], peptides [5,6,7,8] or sugar [9,10,11] with lipids, remains an amazing approach to create new hybrid amphiphilic structures. Owning to their biocompatibility properties and biological functions, hybrid amphiphiles hold considerable potential for biomedical and biotechnological applications. For example, the association of nucleic acid moieties with lipids was realized to enhance the cellular uptake and/or bio-distribution characteristics of nucleoside drugs [12] for cancer therapies. It has been demonstrated that the chemical combination leading to nucleolipids [13,14,15,16] is of particular interest due to the supramolecular properties of these molecules and their potential applications in the biomedical field [17,18,19,20,21]. Interestingly, additional moieties were recently attached to the nucleolipid platforms, including aminoacids [22,23,24] and sugars [25,26]. Among these amphiphiles, glycosyl-nucleolipids (GNLs) [27] hold a special place as nucleoside derivatives. The GNL structure corresponds to the covalent association of a lipid, a nucleoside and a sugar. Each moiety is separated from the other by a triazole linker resulting from the click-chemistry used for their synthesis (Figure 1a).

Most of the GNL amphiphiles exhibit the ability to form gels with either water or organic solvents giving rise to vesicles, nanofibers networks or nanoparticules as evidenced by TEM studies [28,29]. GNLs represent a new class of low molecular weight gelators (LMWGs) [30], which are of particular interest for biomedical applications due to their intrinsic properties, in particular the non-covalent nature of the molecular interactions at work (π-π stacking between the nucleobases and/or the triazole moieties, intermolecular hydrogen bond, etc). The Hydrophilic-Lipophilic balance (HLB) is also an important parameter to take into account for the design of new soluble LMWG. The lipophilicity parameter can be modulated via the insertion of fluorocarbon chains [31] in the amphiphilic structures. Interestingly, this modification provides fluorinated GNL featuring an absence of cytotoxicity (MTT and neutral red assays) [32]. For example, the fluorinated GNL **1** (*aka* GNF, Figure 1b) in its hydrogel form has been used as a scaffold for stem cell culture and differentiation [33], and many complementary studies are still underway to take advantage of this particular property. In contrast, the hydrocarbon single chain GNLs were cytotoxic above 100 µM in human hepatocarcinoma cell line for example [29], which was attributed to a detergent effect of the single chain on the cell membrane, leading to fracture initiation and then cellular death. Consequently, in parallel to the fluoroalkyl (F-alkyl) chain, we also synthesized GNLs featuring a double H-alkyl C18 chain linked to a glycerol backbone (Figure 1c), which was expected to avoid any toxicity towards cells. As expected, GNL **2a** was not cytotoxic (Figure 1c). Moreover, it promotes liposome internalization in adipose stem cells [25] demonstrating again the inherent relevance of GNLs in biomedical applications.

Further investigations in this domain led us to synthesize new GNLs featuring either a single F-alkyl chain or a double H-alkyl chain as hydrophobic moieties. In this contribution we report the synthesis of these new compounds, which have been characterized by NMR, MS, tensiometry and DSC experiments.

## 2. Results and Discussion

### 2.1.Synthesis of New GNLs

**Figure 2:**
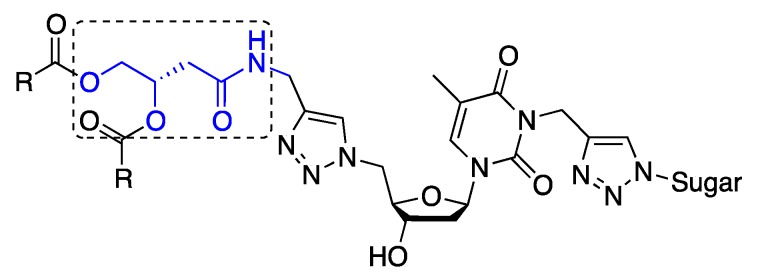
New GNL structure; dotted box highlighting the hydroxybutanamide synthon.

Previous unpublished results from our group obtained with single H-alkyl GNLs, showed that a more stable gel was formed with an amido *vs.* an ether linkage. This prompted us to synthesize new GNLs with modified glycerol backbone structures in favour of a hydroxybutanamide synthon (Figure 2).

**Scheme 1:**
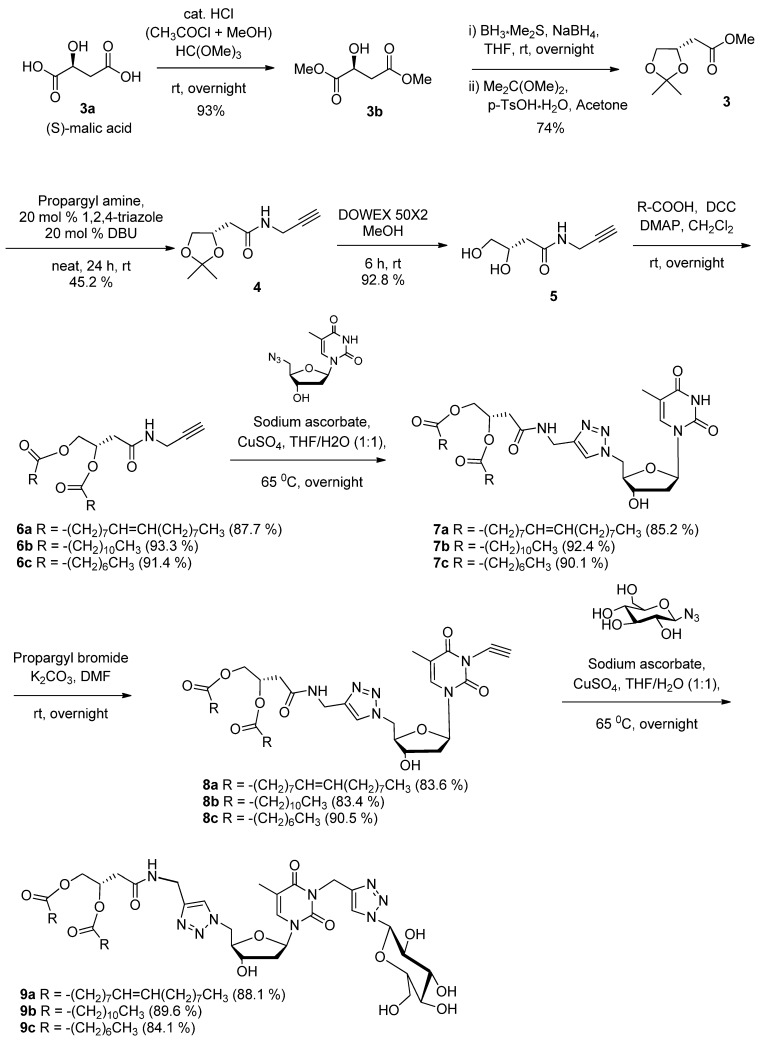
General scheme for synthesis of double chains GNLs **9a**–**c**.

The synthetic pathway leading to different GNLs is described in Scheme 1. The acetonide-ester **3** is the key starting material for the synthesis of all three GNLs and was therefore required in significant quantities. Although commercially available, acetonide-ester **3** is relatively expensive, so it was synthesized, as was the intermediate dimethyl (*S*)-malate **3b**, from (*S*)-malic acid **3a** according to a literature procedure [34]. Aminolysis of unactivated ester **3** with propargylamine turned out to be less effective. The use of sodium methoxide in toluene for amidation of unactivated esters [35] failed. However, aminolysis of acetonide-ester **3** was successfully performed with 1,2,4-triazole anion as catalyst [36], but in a moderate yield, to provide *N*-propargylamide derivative of acetonide **4**. The acetal deprotection of **4** was carried out with Dowex^®^ 50 resin in methanol to give the key intermediate **5** in good yield. Different GNLs were synthesized in four steps starting from compound **5**. Briefly, the fatty acid chain grafting was achieved by means of a DCC/DMAP coupling reaction to afford the lipidic derivatives **6a**–**c**. The first ‘click’ reaction with 5'-azido-5'-deoxythymidine [37] in the presence of CuSO_4_ in a THF/H_2_O mixture provided the expected lipid-triazole-thymidine **7a**–**c**. This lipid-nucleoside intermediates **7a**–**c** were treated with propargyl bromide in the presence of K_2_CO_3_ to afford *N*-propargylated thymidine derivatives **8a**–**c**, which were reacted with 1-azido-1-deoxy-*β*-d-glucopyranoside in the presence of CuSO_4_ in a THF/H_2_O mixture following a second ‘click’ reaction to provide the expected GNLs **9a**–**c** in an average 85% yield.

**Figure 3:**
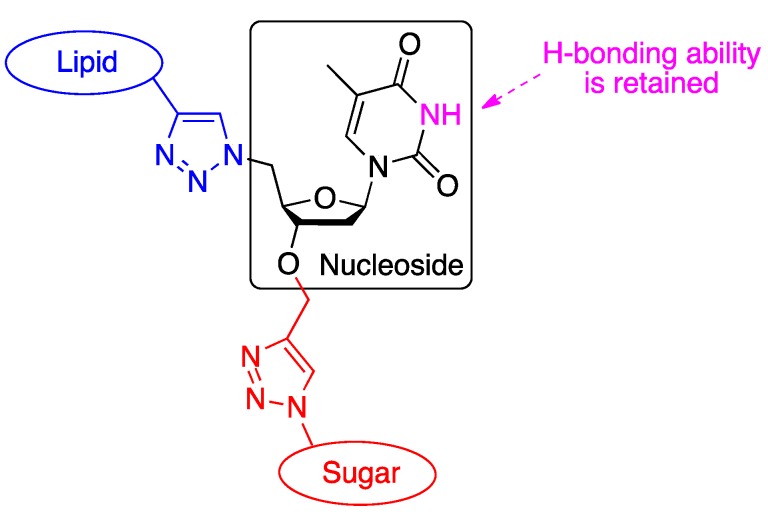
Schematic representation of 2nd generation GNL structure.

It must be emphasized that other GNL structures can be envisioned by connecting the G, N and L moieties differently. Accordingly, the free 3'-ribose position of the nucleoside was used to attach the sugar moiety as depicted below (Figure 3). This new connectivity provides a second generation of GNL featuring a free nucleobase.

This new structure could offer several advantages:

- The GNL synthesis is no more “thymidine-dependent” because any nucleobase can now be used to increase the number of available GNLs, therefore extending the scope of the synthesis.
- Unmodified nucleobases retain their native H-bonding ability, particularly with a complementary base, which may lead to potentially new supramolecular assemblies.

**Scheme 2:**
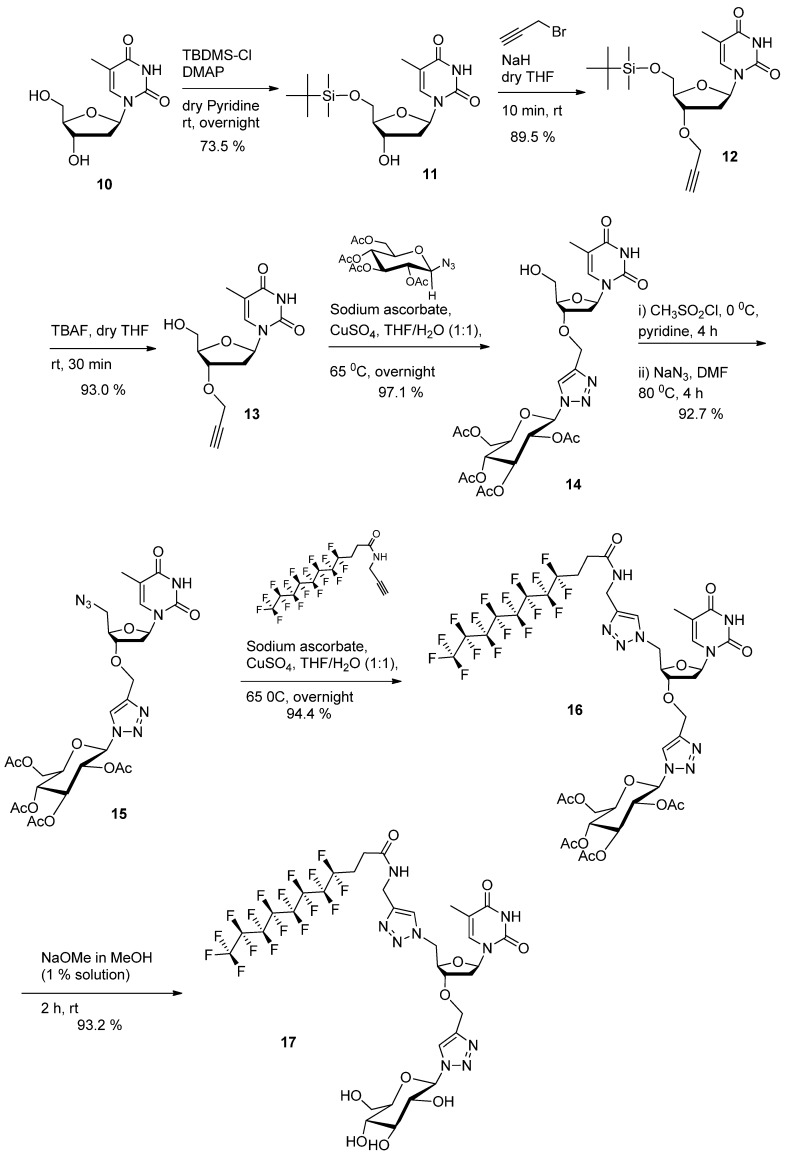
Synthesis of 2nd generation GNF **17** (2nd GNF).

As a preparatory step to a more detailed consideration of this issue, the first so-called “2nd generation” GNL we wanted to investigate is the analog of GNF (2nd GNF). The synthetic route for the preparation of “2nd generation” GNL is shown in Scheme 2. It relies on the preparation of 3'-propargyl thymidine. First, we selectively protected the 5'-hydroxyl group of thymidine with TBDMSCl to obtain 5'-OTBDMS-thymidine **11**. Then the 3'-hydroxy group was propargylated with NaH (2.5 equiv.) and propargyl bromide (2.5 equiv.) in THF at room temperature [38,39]. The selective 3'-*O*-alkylated product **12** was obtained in 90% yield after purification. The TBDMS protection was then removed with TBAF to afford 3'-*O*-propargylated thymidine **13**. Then, 1-azido-2,3,4,6-tetra-*O*-acetyl-β-d-glucopyranoside [40] was reacted with the propargyl derivative **13** in the presence of CuSO_4_ following a first ‘click’ reaction to provide click product **14**. In contrast with the 1st generation GNL synthesis, the use of a protected glucose here allowed the exclusive formation of the desired mesylate intermediate which was directly displaced with sodium azide to yield the azido derivative **15** in 92% yield. Following a second ‘click’ reaction, the *N*-propargyl-1*H*,1*H*,2*H*,2*H*-perfluoroundecanoyl amide [29] was reacted with compound **15** in the presence of CuSO_4_ in a THF/H_2_O mixture to afford the expected acetyl protected GNF **16**. Finally, deacetylation of glucose moiety was carried out with sodium methoxide in methanol at room temperature to give the final compound **17** in 93% yield.

### 2.2.Physicochemical Studies

One of the goals of the present study was to determine the impact of structural modifications, on the self-assembly properties of the new GNLs. The phase transition temperature (*T_m_*) and phase transition enthalpies of the new double chains GNLs were determined by differential scanning calorimetry (DSC). The *T_m_* values of −16.1, 51.7 and 62.1 °C were measured for **9a**, **9b** and **9c**, respectively (Table 1).

Except for compound **9a**, which possess unsaturated oleyl chains, these values are similar to the glycerol based GNL **2a**, suggesting that the hydroxybutanamide moiety does not affect the melting temperature of the GNLs. The phase transition enthalpies for the new GNLs **9a**, **9b** and **9c** and the glycerol based compound **2a** have been also analyzed by DSC. For all compounds, the evolution towards the fluid state of the alkyl chains is endothermic reflecting that amphiphiles possess different organizations below and/or above the *T_m_*. Note that no phase transition was observed for both first and second generation GNFs due to their rigid fluorocarbon chain.

**Table 1:** Melting temperatures and phase transition enthalpies of GNLs (n.o.: not observed).

GNLs	Chains	*T_m_* in °C	ΔH (KJ/mol)
**9a**	Double Oleyl	−16.3 ± 2.8 °C	300 ± 15
**9b**	Double saturated C_12_	51.7 ± 1.8 °C	26.5 ± 5
**9c**	Double saturated C_8_	51,9 ± 1.8 °C	0.46 ± 0.02
**2a**	Double saturated C_18_	56.1 ± 2.4 °C	1.03 ± 0.06
**17**	Single Fluorocarbon	n.a.	n.a.

**Figure 4:**
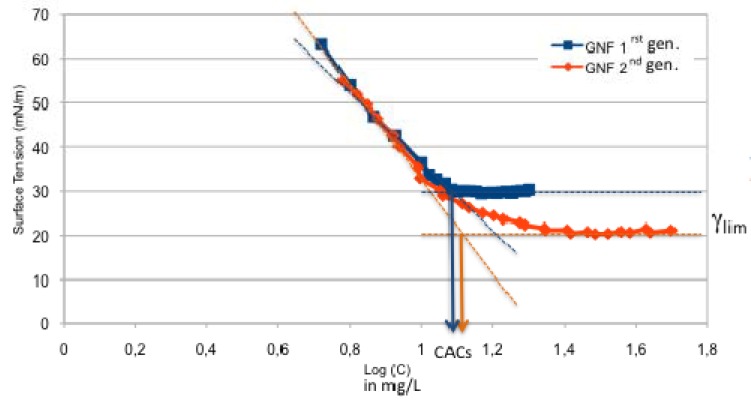
Air–water interfacial tension γ *versus* concentration for 1st and 2nd generations GNFs (blue square and orange diamond) at 25 °C.

The critical aggregation concentrations (CAC’s) of GNFs were determined by air-solution surface tension (γ) measurements as a function of amphiphile concentration (C). Examples of (γ) *versus* (C) curves, measured at 25 °C, are shown in Figure 4. The GNF first and second generation **17** gave breaks in c *versus* Log (C) characteristic of CAC’s of roughly 11 µM for both GNFs indicating that the CAC depends mainly on the hydrophobic segment. Unexpectedly, the structural modification of polar head induces a decrease of the γ_lim_ value (30 mN/M, 20 mN/M, for 1st and 2nd generations GNFs, respectively) likely due to a tight packing of the molecule at the air water interface in the case of the 2nd generation GNF **17**. In contrast with the 1st GNF, which forms gels in water at very low concentration (0.1% w/w), 2nd GNF **17** induces the formation of very viscous colloidal suspensions in similar conditions.

## 3. Experimental

### 3.1. General

All compounds were purchased from Sigma-Aldrich (Saint Quentin Fallavier, France), and Alfa Aesar (Schiltigheim, France) unless otherwise mentioned. Solvents for reactions were purchased from Sigma-Aldrich in the highest quality and from VWR (Fontenay sous Bois, France) for other uses. All the reactions were run under nitrogen atmosphere unless otherwise stated. Analytical thin layer chromatography (TLC) was performed on pre-coated silica gel F_254_ plates with fluorescent indicator from Merck (Fontenay sous Bois, France). The detection of compounds was accomplished using a UV light (254 nm) and visualized on TLC plates by subsequent spraying with 10% conc. H_2_SO_4_ solution in ethanol, followed by heating. Column chromatography was performed with flash silica gel (0.04–0.063 mm) from Merck. All the compounds were characterized using ^1^H and ^13^C Nuclear Magnetic Resonance (NMR) spectroscopy. These NMR spectra were recorded (in CDCl_3_/DMSO-*d_6_*/CD_3_OD obtained from Eurisotop, (Gif sur Yvette, France) on a BRUKER Avance DPX-300 spectrometer (^1^H at 300.13 MHz and ^13^C at 75.46 MHz). The chemical shifts (*δ*) are given in parts per million (ppm) relatively to tetramethylsilane or residual solvent peaks (CHCl_3_: ^1^H: 7.26, ^13^C: 77.0). The coupling constants *J* are given in Hertz (Hz); the peak multiplicity is reported as follows: s = singlet, bs = broad singlet, d = doublet, t = triplet, m = multiplet. High resolution electronspray ionization mass spectra (HR ESI-MS) were performed by the CESAMO (Bordeaux, France) on a QSsat Elite Applied Biosystems mass spectrometer. The instrument is equipped with an ESI source and spectra were recorded in negative mode. The electrospray needle was maintained at 4500 V and operated at room temperature. Samples were introduced by injection through a 10 μL sample loop into a 200 μL/min flow of methanol from the LC pump.

### 3.2. Tensiometer

The critical aggregation concentrations (CACs) of GNFs were determined using a DCAT11 tensiometer from Dataphysics (Voisins le Bretonneux, France). To determine the CAC value of the amphiphiles, freshly prepared solutions of GNFs were added dropwise into distilled water. All additions and measurements were automated and performed at room temperature.

### 3.3. Differential Scanning Calorimetry (DSC)

DSC scans were made with a Mettler-Toledo (Viroflay, France) DSC calorimeter. The GNLs (10 mg) were dissolved in 100 μL of water and hermetically sealed in an aluminum pan. The temperature was increased at 0.5 °C/min to 70 °C where it was held for 2 min. The temperature was then reduced to 10 °C and held at this temperature for 2 min. This heating-cooling cycle was repeated one more times before the sample was held isothermal at 10 °C for 20 min. In the case of oleyl derivative the heating-cooling cycle was done between −30 °C and to 70 °C. The data collected on the second cycle were analyzed. Integrating under the heat flow curve of the main peak afforded the enthalpy of the transition. Mettler Toledo STARe computer program was used to analyze the data.

### 3.4. Syntheses

*Dimethyl (S)-malate* (**3b)**. Compound **3b** was synthesized according to the literature procedure [34]. The data agreed with the literature values. ^1^H-NMR (300 MHz, CDCl_3_): *δ* in ppm 2.73–2.89 (m, 2H, -C*H_2_*-C=O), 3.32 (bs, 1H, -OH), 3.69 (s, 3H, -C*H_3_*-O-C(=O)-CH_2_-), 3.78 (s, 3H, -C*H_3_*-O-C(=O)-CH-), 4.47–4.51 (m, 1H, -C*H*-OH).

*Methyl (4S)-(+)-2,2-dimethyl-1,3-dioxolane-4-acetate* (**3**). Compound **3** was synthesized according to the literature procedure [34]. The data agreed with the literature values. ^1^H-NMR (300 MHz, CDCl_3_): *δ* in ppm 1.33 (s, 3H, -C-C*H_3_*-), 1.39 (s, 3H, -C-C*H_3_*-), 2.46–2.54 (m, 1H, -C*H'*H≡-C=O), 2.66–2.73 (m, 1H, -CH'*H*≡-C=O), 3.60–3.65 (m, 1H, -O- C*H'*H≡-), 3.68 (s, 3H, -O-C*H_3_*), 4.11–4.16 (m, 1H, -O- CH'*H*≡-), 4.45 (quintet, 1H, -O-C*H*-, *J* = 6.45 Hz).

*(S)-2-(2,2-Dimethyl-1,3-dioxolan-4-yl)-N-(prop-2-ynyl)acetamide* (**4**). A reaction mixture consisting of propargyl amine (2.95 g, 3.5 mL, 53.63 mmol, 1.0 equiv), methyl (4S)-(+)-2,2-dimethyl-1,3-dioxolane-4-acetate (**3**, 11.2 g, 64.36 mmol, 1.2 equiv), 1,2,4-triazole (740 mg, 10.73 mmol, 0.20 equiv.) and DBU (1.63 g, 1.6 mL, 10.73 mmol, 0.20 equiv.) was stirred at room temperature for 48 h and then applied directly to a silica gel chromatographic column (diethyl ether-TEA 98:2). Product **4** was then isolated as a colourless oil. Yield: 4.77 g (45.2%). R*_f_*: 0.5 (diethyl ether). ^1^H-NMR (300 MHz, CDCl_3_): *δ* in ppm 1.35 (s, 3H, CH_3_), 1.43 (s, 3H, CH_3_), 2.22 (t, 1H, -C≡C*H*, *J* = 2.5 Hz), 2.45–2.54 (m, 2H, -C*H_2_*-C=O), 3.59–3.64 (m, 1H, -O-C*H′*H″), 3.91–4.07 (m, 2H, -NH-C*H_2_*-C≡), 4.09–4.14 (m, 1H, -O-CH′*H″*), 4.36–4.45 (m, 1H, -C*H*-), 6.43 (bs, 1H, -N*H*-C=O-). ^13^C-NMR (75 MHz, CDCl_3_): *δ* in ppm 25.5 Η(-*C*H_3_), 26.8 (-*C*H_3_), 29.0 (-*C*H_2_-NH), 40.1 (-*C*H_2_-C=O), 68.9 (-O-*C*H_2_), 71.5 (≡*C*H), 72.2 (-*C*H), 79.4 (-*C*≡), 109.5 (O-*C*-O), 169.6 (-NH-*C*=O). High-resolution ESI-MS *m/z* calcd. for C_10_H_15_NO_3_Na 220.0944 [M+Na]^+^, found 220.0952.

*(S)-3,4-Dihydroxy-N-(prop-2-ynyl)butanamide* (**5**). A solution of compound **4** (4.2 g, 21.32 mmol) in methanol (100 mL) was treated with Dowex^®^ 50X2 (10 g) at room temperature until completion of the reaction (≈4 to 5 h) as evidenced by TLC (methanol-ethyl acetate: 1:9). After removal of the solvent under reduced pressure, the crude deprotected product applied directly to a silica gel chromatographic column (methanol-ethyl acetate 1:9). Product **5** was isolated as a white solid. Yield: 3.1 g (92.8%). R*_f_*: 0.33 (methanol-ethyl acetate 1:9). ^1^H-NMR (300 MHz, CD_3_OD): *δ* in ppm 2.26–2.45 (m, 2H,-C*H_2_*-C=O), 2.59 (t, 1H, -C≡C*H*, *J* = 2.5 Hz), 3.48 (d, 2H, *J* = 5.5, -C*H_2_*OH), 3.95–3.99 (m, 2H, NH-C*H_2_*-C≡), 4.00-4.09 (m, 1H, -C*H*-). ^13^C-NMR (75 MHz, CD_3_OD): *δ* in ppm 29.4 (-*C*H_2_-NH), 40.9 (-*C*H_2_-C=O), 66.8 (-*C*H_2-_OH), 70.4 (-*C*H-OH), 72.2 (≡*C*H), 80.5 (-*C*≡), 173.6 (-NH-*C*=O). High-resolution ESI-MS *m/z* calcd. for C_7_H_11_NO_3_Na 180.0631 [M+Na]^+^, found 180.0625.

*General procedure for synthesis of coupling products* **6a**, **6b** *and* **6c**. To a solution of compound **5** (1 equiv.) in anhydrous dichloromethane was added DCC (3 equiv.), DMAP (3 equiv.) and the appropriate fatty acid **5a** or **5b** or **5c** (3 equiv.), and the mixture was stirred overnight at room temperature. The reaction mixture was filtered and the filtrate was concentrated under reduced pressure. The crude product was purified by column chromatography on silica gel eluting with different ratios of ethylacetate/hexane to obtain pure product **5a**, **5b** and **5c**.

*(Z)-((S)-4-Oxo-4-(prop-2-ynylamino)butane-1,2-diyl) dioleate* (**6a**). Anhydrous dichloromethane (15 mL), compound **5** (60 mg, 0.382 mmol, 1.0 equiv.), DCC (236 mg, 1.146 mmol, 3.0 equiv.), DMAP (140 mg, 1.146 mmol, 3.0 equiv.), oleic acid (**5a**, 324 mg, 364 μL, 1.146 mmol, 3.0 equiv.). Product **6a** was isolated after purification on silica gel (ethyl acetate-hexane 1:3) as a white solid. Yield: 230 mg (87.7%). R*_f_*: 0.32 (ethyl acetate-hexane 1:3). ^1^H-NMR (300 MHz, CDCl_3_): *δ* in ppm 0.87 (t, 6H, 2 CH_3_, *J* = 6.6 Hz), 1.26–1.29 (m, 40H, 20CH_2_ of the dioleyl chain), 1.51–1.62 (m, 4H, 2C*H_2_*-CH_2_-C=O), 1.88–2.09 (m, 8H, 2C*H_2_*-CH=CH-C*H_2_*), 2.23 (t, 1H, -C≡C*H*, *J* = 2.5 Hz), 2.27–2.33 (m, 4H, 2C*H_2_*-C=O of the dioleyl chain), 2.53 (d, 2H, C*H_2_*-C=O, *J* = 6.4 Hz), 4.02–4.05 (m, 2H, NH-*H_2_*-C≡), 4.12–4.18 (m, 1H, -O-C*H′*H″), 4.31-4.36 (m, 1H, -O-CH′*H″*), 5.28–5.35 (m, 4H, 2C*H*=C*H* of the dioleyl chain), 5.37–5.42 (m, 1H, -C*H*-), 5.93 (bs, 1H, -N*H*-C=O-). ^13^C-NMR (75 MHz, CDCl_3_): *δ* in ppm 14.1 (2*C*H_3_), 22.7 (2*C*H_2_-CH_3_), 24.8 (2*C*H_2_-CH_2_-C=O), 27.1 (*C*H_2_-CH=CH-*C*H_2_), 27.2 (*C*H_2_-CH=CH-*C*H_2_), 29.0–29.7 (CH_2_ of the dioleyl chain and -*C*H_2_-NH), 31.9 (2*C*H_2_-CH_2_-CH_3_), 34.0 (*C*H_2_-C=O of chain), 34.2 (*C*H_2_-C=O of chain), 37.9 (-*C*H_2_-C=O), 64.2 (-*C*H_2-_O-), 68.4 (≡*C*H), 71.8 (-*C*H-O-), 79.1 (-*C*≡), 129.7 (-*C*H=*C*H-), 130.0 (-*C*H=*C*H-), 168.2 (-NH-*C*=O), 172.9 (-O-C=O), 173.3 (-O-C=O). High-resolution ESI-MS *m/z* calcd. for C_43_H_75_NO_5_Na 708.5537 [M+Na]^+^, found 708.5532.

*(S)-4-Oxo-4-(prop-2-ynylamino)butane-1,2-diyl didodecanoate* (**6b**). Anhydrous dichloromethane (20 mL), compound **5** (150 mg, 0.95 mmol, 1.0 equiv.), DCC (590 mg, 2.86 mmol, 3.0 equiv.), DMAP (349 mg, 2.86 mmol, 3.0 equiv.), lauric acid (**5b**, 573 mg, 2.86 mmol, 3.0 equiv.). Product **6b** was isolated after purification on silica gel (ethyl acetate-hexane 3:7) as a white solid. Yield: 465 mg (93.3%). R*_f_*: 0.38 (ethyl acetate-hexane 3:7). ^1^H-NMR (300 MHz, CDCl_3_): *δ* in ppm 0.87 (t, 6H, 2 CH_3_, *J* = 6.6 Hz), 1.25 (s, 32H, 16CH_2_ of the dilauryl chain), 1.51–1.69 (m, 4H, 2C*H_2_*-CH_2_-C=O), 2.23 (t, 1H, -C≡C*H*, *J* = 2.54 Hz), 2.27–2.33 (m, 4H, 2C*H_2_*-C=O of the dilauryl chain), 2.53 (d, 2H, C*H_2_*-C=O, *J* = 6.4 Hz), 4.03–4.05 (m, 2H, NH-C*H_2_*-C≡), 4.12–4.18 (m, 1H, -O-C*H′*H″), 4.31–4.36 (m, 1H, -O-CH′*H″*), 5.35–5.44 (m, 1H,-C*H*-), 5.92 (bs, 1H, -N*H*-C=O-). ^13^C-NMR (75 MHz, CDCl_3_): *δ* in ppm 14.1 (2*C*H_3_), 22.6 (2*C*H_2_-CH_3_), 24.8 (2*C*H_2_-CH_2_-C=O), 29.0–29.6 (CH_2_ of the dilauryl chain and -*C*H_2_-NH), 31.9 (2*C*H_2_-CH_2_-CH_3_), 34.0 (*C*H_2_-C=O of chain), 34.2 (*C*H_2_-C=O of chain), 37.8 (-*C*H_2_-C=O), 64.1 (-*C*H_2-_O-), 68.4 (≡*C*H), 71.7 (-*C*H-O-), 79.1 (-*C*≡), 168.3 (-NH-*C*=O), 172.9 (-O-C=O), 173.4 (-O-C=O). High-resolution ESI-MS *m/z* calcd. for C_31_H_55_NO_5_Na 544.3972 [M+Na]^+^, found 544.3978.

*(S)-4-Oxo-4-(prop-2-ynylamino)butane-1,2-diyl dioctanoate* (**6c**). Anhydrous dichloromethane (20 mL), compound **5** (130 mg, 0.83 mmol, 1.0 equiv.), DCC (512 mg, 2.49 mmol, 3.0 equiv.), DMAP (303 mg, 2.49 mmol, 3.0 equiv.), octanoic acid (**5c**, 359 mg, 395 μL, 2.49 mmol, 3.0 equiv.). Product **6c** was isolated after purification on silica gel (ethyl acetate-hexane 3:7) as a colourless oil (which may turn into a white solid upon standing). Yield: 310 mg (91.4%). R*_f_*: 0.6 (ethyl acetate-hexane 2:3). ^1^H-NMR (300 MHz, CDCl_3_): *δ* in ppm 0.85 (t, 6H, 2 CH_3_, *J* = 6.6 Hz), 1.25 (s, 16H, 8CH_2_ of the dioctanoyl chain), 1.56–1.60 (m, 4H, 2C*H_2_*-CH_2_-C=O), 2.22 (t, 1H, -C≡C*H*, *J* = 2.54 Hz), 2.25–2.32 (m, 4H, 2C*H_2_*-C=O of the dioctanoyl chain), 2.53 (d, 2H, C*H_2_*-C=O, *J* = 6.4 Hz), 4.00–4.03 (m, 2H, NH-C*H_2_*-C≡), 4.11–4.17 (m, 1H, -O-C*H′*H″), 4.29–4.35 (m, 1H, -O-CH′*H″*), 5.34–5.42 (m, 1H, -C*H*-), 6.12 (bs, 1H, -N*H*-C=O-). ^13^C-NMR (75 MHz, CDCl_3_): *δ* in ppm 14.0 (2*C*H_3_), 22.5 (2*C*H_2_-CH_3_), 24.8 (2*C*H_2_-CH_2_-C=O), 28.9–29.2 (CH_2_ of the dioctanoyl chain and -*C*H_2_-NH), 31.6 (2*C*H_2_-CH_2_-CH_3_), 34.0 (*C*H_2_-C=O of chain), 34.2 (*C*H_2_-C=O of chain), 37.7 (-*C*H_2_-C=O), 64.1 (-*C*H_2-_O-), 68.3 (≡*C*H), 71.7 (-*C*H-O-), 79.2 (-*C*≡), 168.3 (-NH-*C*=O), 172.9 (-O-C=O), 173.4 (-O-C=O). High-resolution ESI-MS *m/z* calcd. for C_23_H_39_NO_5_Na 432.2720 [M+Na]^+^, found 432.2722.

*General procedure for the synthesis of the first click products* **7a**, **7b** *and* **7c**. The above coupling products **6a** or **6b** or **6c**, sodium ascorbate and copper sulfate were added to 5'-azido-5'-deoxythymidine [37] in a water/THF (1:1) mixture. The reaction mixture was maintained at 65 °C under stirring for 10 h. After cooling to room temperature, the solvent was evaporated. The residual solid was dissolved in DCM and successively washed with water and brine. The organic layer was dried over Na_2_SO_4_ and evaporated under reduced pressure. The crude product was purified by column chromatography on silica gel eluted with different ratios of methanol/ethyl acetate to obtain purified compounds **7a**, **7b** or **7c**.

*(Z)-((S)-4-(1-(((2R,3S,5R)-3-Hydroxy-5-(5-methyl-2,4-dioxo-3,4-dihydropyrimidin-1(2H)yl) tetrahydrofuran -2-yl)methyl)-1H-1,2,3-triazol-4-ylamino)-4-oxobutane-1,2-diyl) dioleate* (**7a**). Coupling product **6a** (210 mg, 0.306 mmol, 1.0 equiv.), 5'-azido-5'-deoxythymidine (81.7 mg, 0.306 mmol, 1.0 equiv.), sodium ascorbate (31 mg, 0.153 mmol, 0.5 equiv.), copper sulfate (13 mg, 0.0765 mmol, 0.25 equiv.), water/THF (1:1) (20 mL). Product **7a** was isolated after purification on silica gel (methanol-ethyl acetate:2:98) as a white solid. Yield: 248 mg (85.2%). R*_f_*: 0.28 (methanol-ethyl acetate 2:98). ^1^H-NMR (300 MHz, CDCl_3_): *δ* in ppm 0.87 (t, 6H, 2 CH_3_ of the dioleyl chain, *J* = 6.7 Hz), 1.26–1.28 (m, 40H, 20CH_2_ of the dioleyl chain), 1.41–1.66 (m, 4H, 2C*H_2_*-CH_2_-C=O), 1.88 (s, 3H, CH_3_(base)), 1.96–2.09 (m, 8H, 2C*H_2_*-CH=CH-C*H_2_*), 2.23–2.42 (m, 6H, 2C*H_2_*-C=O of the dioleyl chain, 2H2′(sugar)), 2.47–2.60 (m, 2H, C*H_2_*-C=O), 3.38 (bs, 1H, -OH), 4.06–4.12 (m, 1H, NH-C*H′*H″-C=), 4.15–4.25 (m, 1H, -O-C*H′*H″), 4.28–4.36 (m, 1H, -O-CH′*H″*), 4.45–4.77 (m, 5H, NH-CH′*H″*-C=, H3′, H4′, 2H5′), 5.27–5.35 (m, 4H, 2C*H*=C*H* of the dioleyl chain), 5.37–5.46 (m, 1H, -C*H*-), 6.00 (t, 1H, *J* = 6.4 Hz, H1′), 6.98 (s, 1H, triazole), 7.17 (bs, 1H, -N*H*-C=O-), 7.77 (s, 1H, H-6(base)), 9.79 (s, 1H, -NH(base)). ^13^C-NMR (75 MHz, CDCl_3_): *δ* in ppm 12.4 (CH_3_(base)), 14.1 (2*C*H_3_ of the dioleyl chain), 22.6 (2*C*H_2_-CH_3_), 24.9 (2*C*H_2_-CH_2_-C=O), 27.2 (2*C*H_2_-CH=CH-*C*H_2_), 29.0–29.7 (CH_2_ of the dioleyl chain), 31.9 (2*C*H_2_-CH_2_-CH_3_), 34.0 (*C*H_2_-C=O of chain), 34.3 (*C*H_2_-C=O of chain), 34.7 (-*C*H_2_-NH), 37.6 (C2′), 38.6 (-*C*H_2_-C=O), 51.3 (C5′), 64.4 (-*C*H_2-_O-), 68.5 (C3′), 71.2 (-*C*H-O-), 83.8 (C4′), 87.1 (C1′), 111.3 (C5-thymine base), 124.5 (-CH triazole), 129.6 (-*C*H=*C*H-), 130.0 (-*C*H=*C*H-), 137.1 (C6-thymine base), 144.4 (=C- triazole), 150.3 (C=O(2) thymine base), 164.0 (C=O(4) thymine base), 169.3 (-NH-*C*=O), 173.2 (-O-C=O of the oleyl chain), 173.6 (-O-C=O of the oleyl chain). High-resolution ESI-MS *m/z* calcd. for C_53_H_88_N_6_O_9_Na 975.6505 [M+Na]^+^, found 975.6548.

*(S)-4-(1-(((2R,3S,5R)-3-Hydroxy-5-(5-methyl-2,4-dioxo-3,4-dihydropyrimidin-1(2H)-yl)tetrahydrofuran-2-yl)methyl)-1H-1,2,3-triazol-4-ylamino)-4-oxobutane-1,2-diyl didodecanoate* (**7b**). Coupling product **6b** (350 mg, 0.67 mmol, 1.0 equiv.), 5'-azido-5'-deoxythymidine (180 mg, 0.67 mmol, 1.0 equiv.), sodium ascorbate (66 mg, 0.34 mmol, 0.5 equiv.), copper sulfate (27 mg, 0.17 mmol, 0.25 equiv.), water/THF (1:1) (15 mL). Product **7b** was isolated after purification on silica gel (methanol-ethyl acetate 0:100 to 5:95) as a white solid. Yield: 490 mg (92.4%). R*_f_*: 0.25 (methanol-ethyl acetate 2.5:97.5). ^1^H-NMR (300 MHz, CDCl_3_:CD_3_OD(9.5:0.5)): *δ* in ppm 0.75 (t, 6H, 2 CH_3_ of the dilauryl chain, *J* = 6.6 Hz), 1.13 (s, 32H, 16CH_2_ of the dilauryl chain), 1.35–1.54 (m, 4H, 2C*H_2_*-CH_2_-C=O), 1.78 (s, 3H, CH_3_(base)), 2.06–2.23 (m, 6H, 2C*H_2_*-C=O of the dilauryl chain, 2H2′(sugar)), 2.31–2.45 (m, 2H, C*H_2_*-C=O), 3.97–4.04 (m, 2H,-O-C*H_2_*), 4.15–4.21 (m, 2H, 2H5′(base)), 4.26 (s, 2H, NH-C*H_2_*-C=), 4.5–4.62 (m, 2H, H3′(sugar), H4′(sugar)), 5.22–5.34 (m, 1H, -C*H*-), 5.98 (t, 1H, *J* = 6.7 Hz, H1′(sugar)), 6.90 (s, 1H, triazole), 7.65 (s, 1H, H-6(base)). ^13^C-NMR (75 MHz, CDCl_3_:CD_3_OD(9.5:0.5)): *δ* in ppm 12.1 (CH_3_(base)), 13.9 (2*C*H_3_ of the dilauryl chain), 22.5 (2*C*H_2_-CH_3_), 24.7 (2*C*H_2_-CH_2_-C=O), 28.9–29.4 (CH_2_ of the dilauryl chain), 31.7 (2*C*H_2_-CH_2_-CH_3_), 33.9 (*C*H_2_-C=O of chain), 34.1 (*C*H_2_-C=O of chain), 34.2 (-*C*H_2_-NH), 37.2 (C2′), 38.5 (-*C*H_2_-C=O), 51.1 (C5′), 64.2 (-*C*H_2-_O-), 68.3 (C3′), 70.6 (-*C*H-O-), 83.5 (C4′), 86.0 (C1′), 111.1 (C5-thymine base), 124.5 (-CH triazole), 136.4 (C6-thymine base), 144.3 (=C- triazole), 150.3 (C=O(2) thymine base), 164.2 (C=O(4) thymine base), 169.5 (-NH-*C*=O), 173.2 (-O-C=O of the lauryl chain), 173.6 (-O-C=O of oleyl chain). High-resolution ESI-MS *m/z* calcd. for C_41_H_68_N_6_O_9_Na 811.4939 [M+Na]^+^, found 811.4972.

*(S)-4-(1-(((2R,3S,5R)-3-Hydroxy-5-(5-methyl-2,4-dioxo-3,4-dihydropyrimidin-1(2H)-yl)tetrahydrofuran-2-yl)methyl)-1H-1,2,3-triazol-4-ylamino)-4-oxobutane-1,2-diyl dioctanoate* (**7c**). Coupling product **6c** (450 mg, 1.1 mmol, 1.0 equiv.), 5'-azido-5'-deoxythymidine (294 mg, 1.1 mmol, 1.0 equiv.), sodium ascorbate (44 mg, 0.22 mmol, 0.2 equiv.), copper sulfate (18 mg, 0.11 mmol, 0.1 equiv.), water/THF (1:1) (20 mL). Product **7c** was isolated after purification on silica gel (methanol-ethyl acetate 1:9) as a white solid. Yield: 670 mg (90.1%). R*_f_*: 0.33 (methanol-ethyl acetate 5:95). ^1^H-NMR (300 MHz, CD_3_OD): *δ* in ppm 0.90 (t, 6H, 2 CH_3_ of the dioctanoyl chain, *J* = 6.7 Hz), 1.29 (s, 16H, 8CH_2_ of the dioctanoyl chain), 1.48–1.67 (m, 4H, 2C*H_2_*-CH_2_-C=O), 1.90 (s, 3H, CH_3_(base)), 2.21–2.33 (m, 6H, 2C*H_2_*-C=O of the dioctanoyl chain, 2H2′ (sugar)), 2.43–2.60 (m, 2H, C*H_2_*-C=O), 4.03–4.18 (m, 2H, -O-C*H_2_*), 4.32–4.46 (m, 4H, 2H5′(base), NH-C*H_2_*-C=), 4.62–4.79 (m, 2H, H3′(sugar), H4′(sugar)), 5.37–5.49 (m, 1H, -C*H*-), 6.19 (t, 1H, *J* = 6.4 Hz, H1′), 7.32 (s, 1H, triazole), 7.88 (s, 1H, H-6(base)). ^13^C-NMR (75 MHz, CD_3_OD): *δ* in ppm 12.5 (CH_3_(base)), 14.5 (2*C*H_3_ of the dioctanoyl chain), 23.7 (2*C*H_2_-CH_3_), 26.0 (2*C*H_2_-CH_2_-C=O), 30.1-30.2 (CH_2_ of the dioctanoyl chain), 32.9 (2*C*H_2_-CH_2_-CH_3_), 34.9 (*C*H_2_-C=O of chain), 35.1 (*C*H_2_-C=O of chain), 35.6 (-*C*H_2_-NH), 38.2 (C2′), 39.6 (-*C*H_2_-C=O), 52.8 (C5′), 65.5 (-*C*H_2-_O-), 69.9 (C3′), 72.5 (-*C*H-O-), 85.6 (C4′), 87.0 (C1′), 111.9 (C5-thymine base), 125.4 (-CH triazole), 138.2 (C6-thymine base), 146.1 (=C- triazole), 152.1 (C=O(2) thymine base), 166.3 (C=O(4) thymine base), 171.7 (-NH-*C*=O), 174.4 (-O-C=O of the octanoyl chain), 174.9 (-O-C=O of octanoyl chain). High-resolution ESI-MS *m/z* calcd. for C_33_H_52_N_6_O_9_Na 699.3687 [M+Na]^+^, found 699.3683.

*General procedure for the synthesis of the N-propargyl derivatives of click products* **8a**, **8b** *and* **8c**. Anhydrous DMF was added under nitrogen to the click products **7a**, **7b** or **7c**, followed by K_2_CO_3_. Propargyl bromide was then added at room temperature, and the reaction mixture was stirred overnight. DMF was removed under reduced pressure, the residual solid was dissolved in ethyl acetate (or dichloromethane for **8a**) and was washed twice with water and once with brine. The organic phase was dried over Na_2_SO_4_ and evaporated under reduced pressure. The crude product was purified by column chromatography on silica gel eluted with different ratios of methanol-ethyl acetate to afford the purified click products **8a**, **8b** or **8c**.

*(Z)-((S)-4-(1-(((2R,3S,5R)-3-Hydroxy-5-(5-methyl-2,4-dioxo-3-(prop-2-ynyl)-3,4-dihydropyrimidin-1(2H)-yl) tetrahydrofuran-2-yl)methyl)-1H-1,2,3-triazol-4-ylamino)-4-oxobutane-1,2-diyl) dioleate* (**8a**). Click product **7a** (190 mg, 0.2 mmol, 1 equiv.), propargyl bromide (59.5 mg, 43.1 μL (80 wt.% solution in toluene), 0.4 mmol, 2 equiv.), K_2_CO_3_ (54 mg, 0.4 mmol, 2 equiv.), dry DMF (10 mL). Product **8a** was isolated after purification on silica gel (methanol-ethyl acetate 0:100 to 2:98) as a colourless oil. Yield: 185 mg (83.6%). R*_f_*: 0.41 (ethyl acetate). ^1^H-NMR (300 MHz, CDCl_3_): *δ* in ppm 0.87 (t, 6H, 2 CH_3_ of the dioleyl chain, *J* = 6.7 Hz), 1.26–1.28 (m, 40H, 20CH_2_ of the dioleyl chain), 1.44–1.69 (m, 4H, 2C*H_2_*-CH_2_-C=O), 1.95 (s, 3H, CH_3_(base)), 1.97–2.04 (m, 8H, 2C*H_2_*-CH=CH-C*H_2_*), 2.23–2.39 (m, 7H, 2C*H_2_*-C=O of the dioleyl chain, 2H2′(sugar), -C≡C*H*), 2.47–2.58 (m, 2H, C*H_2_*-C=O), 2.98 (bs, 1H, -OH), 4.05–4.12 (m, 1H, -O-C*H′*H″-C=), 4.21–4.31 (m, 2H, -O-CH′*H″*, NH-C*H′*H″-C=), 4.41–4.47 (m, 3H, NH-CH′*H″*-C=, C*H′*H″-C≡, H3′), 4.63–4.76 (m, 4H, H4′, 2H5′, -CH′*H″*-C≡), 5.27–5.35 (m, 4H, 2C*H*=C*H* of the dioleyl chain), 5.37–5.44 (m, 1H, -C*H*-), 6.14 (t, 1H, *J* = 6.7 Hz, H1′), 6.82 (bs, 1H, -N*H*-C=O-), 6.96 (s, 1H, triazole), 7.74 (s, 1H, H-6(base)). ^13^C-NMR (75 MHz, CDCl_3_): *δ* in ppm 13.1 (CH_3_(base)), 14.1 (2*C*H_3_ of the dioleyl chain), 22.6 (2*C*H_2_-CH_3_), 24.8 (2*C*H_2_-CH_2_-C=O), 27.2 (2*C*H_2_-CH=CH-*C*H_2_), 29.0–29.7 (CH_2_ of the dioleyl chain), 30.4 (-*C*H2-C≡), 31.9 (2*C*H_2_-CH_2_-CH_3_), 34.0 (*C*H_2_-C=O of chain), 34.2 (*C*H_2_-C=O of chain), 34.7 (-*C*H_2_-NH), 37.8 (C2′), 38.6 (-*C*H_2_-C=O), 51.4 (C5′), 64.3 (-*C*H_2-_O-), 68.4 (C3′), 70.9 (≡CH), 71.5 (-*C*H-O-), 78.1 (-C≡), 83.9 (C4′), 87.5 (C1′), 110.7 (C5-thymine base), 124.4 (-CH triazole), 129.6 (-*C*H=*C*H-), 130.0 (-*C*H=*C*H-), 135.0 (C6-thymine base), 144.3 (=C- triazole), 150.0 (C=O(2) thymine base), 162.2 (C=O(4) thymine base), 169.1 (-NH-*C*=O), 173.2 (-O-C=O of the oleyl chain), 173.6 (-O-C=O of oleyl chain). High-resolution ESI-MS *m/z* calcd. for C_56_H_90_N_6_O_9_Na 1013.6661 [M+Na]^+^, found 1013.6711.

*(S)-4-(1-(((2R,3S,5R)-3-Hydroxy-5-(5-methyl-2,4-dioxo-3-(prop-2-ynyl)-3,4-dihydropyrimidin-1(2H)-yl) tetrahydrofuran-2-yl)methyl)-1H-1,2,3-triazol-4-ylamino)-4-oxobutane-1,2-diyl didodecanoate* (**8b**). Click product **7b** (440 mg, 0.56 mmol, 1 equiv.), propargyl bromide (116 mg, 120 μL (80 wt.% solution in toluene), 1.12 mmol, 2 equiv.), K_2_CO_3_ (154 mg, 1.12 mmol, 2 equiv.), dry DMF (15 mL). Product **8b** was isolated after purification on silica gel (methanol-ethyl acetate 0:100 to 2:98) as a colourless oil. Yield: 385 mg (83.4%). R*_f_*: 0.30 (methanol-ethyl acetate 2:98). ^1^H-NMR (300 MHz, CDCl_3_): *δ* in ppm 0.86 (t, 6H, 2CH_3_ of the dilauryl chain, *J* = 6.6 Hz), 1.23 (s, 32H, 16CH_2_ of the dilauryl chain), 1.46–1.65 (m, 4H, 2C*H_2_*-CH_2_-C=O), 1.93 (s, 3H, CH_3_(base)), 2.11–2.42 (m, 7H, 2C*H_2_*-C=O of the dilauryl chain, 2H2′(sugar), -C≡C*H*), 2.47–2.56 (m, 2H, C*H_2_*-C=O), 4.05–4.11 (m, 1H, -O-C*H′*H″-C=), 4.19–4.30 (m, 2H, -O-CH′*H″*, NH-C*H′*H″-C=), 4.37–4.49 (m, 3H, NH-CH′*H″*-C=, C*H′*H″-C≡, H3′), 4.58–4.80 (m, 4H, H4′, 2H5′, -CH′*H″*-C≡), 5.35–5.40 (m, 1H, -C*H*-), 6.15 (t, 1H, *J* = 6.7 Hz, H1′), 6.96 (bs, 2H, -N*H*-C=O, triazole), 7.73 (s, 1H, H-6(base)). ^13^C-NMR (75 MHz, CDCl_3_): *δ* in ppm 13.1 (CH_3_(base)), 14.1 (2*C*H_3_ of the dilauryl chain), 22.6 (2*C*H_2_-CH_3_), 24.8 (2*C*H_2_-CH_2_-C=O), 29.0–29.6 (CH_2_ of the dilauryl chain), 30.4 (-*C*H2-C≡), 31.8 (2*C*H_2_-CH_2_-CH_3_), 34.0 (*C*H_2_-C=O of chain), 34.2 (*C*H_2_-C=O of chain), 34.7 (-*C*H_2_-NH), 37.7 (C2′), 38.5 (-*C*H_2_-C=O), 51.2 (C5′), 64.3 (-*C*H_2-_O-), 68.4 (C3′), 70.9 (≡CH), 71.3 (-*C*H-O-), 78.1 (-C≡), 83.9 (C4′), 87.2 (C1′), 110.7 (C5-thymine base), 124.3 (-CH triazole), 135.0 (C6-thymine base), 144.4 (=C- triazole), 150.0 (C=O(2) thymine base), 162.2 (C=O(4) thymine base), 169.1 (-NH-*C*=O), 173.2 (-O-C=O of the lauryl chain), 173.6 (-O-C=O of oleyl chain). High-resolution ESI-MS *m/z* calcd. for C_44_H_70_N_6_O_9_Na 849.5096 [M+Na]^+^, found 849.5111.

*(S)-4-(1-(((2R,3S,5R)-3-Hydroxy-5-(5-methyl-2,4-dioxo-3-(prop-2-ynyl)-3,4-dihydropyrimidin-1(2H)-yl)tetrahydrofuran-2-yl)methyl)-1H-1,2,3-triazol-4-ylamino)-4-oxobutane-1,2-diyl dioctanoate* (**8c**). Click product **7c** (565 mg, 0.84 mmol, 1 equiv), propargyl bromide (248 mg, 180 μL (80 wt.% solution in toluene), 1.67 mmol, 2 equiv.), K_2_CO_3_ (231 mg, 1.67 mmol, 2 equiv.), dry DMF (20 mL). Product **8c** was isolated after purification on silica gel (methanol-ethyl acetate 5:95) as a colourless oil. Yield: 540 mg (90.5%). R*_f_*: 0.45 (methanol-ethyl acetate 5:95). ^1^H-NMR (300 MHz, DMSO-d_6_): *δ* in ppm 0.84 (t, 6H, 2CH_3_ of dioctanoyl chain, *J* = 6.2 Hz), 1.23 (s, 16H, 16CH_2_ of the dioctanoyl chain), 1.37–1.57 (m, 4H, 2C*H_2_*-CH_2_-C=O), 1.87 (s, 3H, CH_3_(base)), 2.15–2.27 (m, 6H, 2C*H_2_*-C=O of the dioctanoyl chain, 2H2′(sugar)), 2.44 (d, 2H, *J* = 6.6 Hz, C*H_2_*-C=O), 3.10 (t, 1H, *J* = 2.26 Hz, -C≡C*H*), 3.99–4.11 (m, 2H, -O-C*H_2_*), 4.22–4.30 (m, 4H, 2H5′(base), NH-C*H_2_*-C=), 4.52 (s, 2H, -C*H_2_*-C≡), 4.56–4.73 (m, 2H, H3′(sugar), H4′(sugar)), 5.26–5.34 (m, 1H, -C*H*-), 5.56 (d, 1H, J = 4.14 Hz, -NH-C=O, ), 6.21 (t, 1H, *J* = 6.9 Hz, H1′), 7.49 (s, 1H, triazole), 8.49 (t, 1H, *J* = 5.47 Hz, H-6(base)). ^13^C-NMR (75 MHz, DMSO-d_6_): *δ* in ppm 12.7 (CH_3_(base)), 14.0 (2*C*H_3_ of the dioctanoyl chain), 22.1 (2*C*H_2_-CH_3_), 24.4–24.5 (2*C*H_2_-CH_2_-C=O), 28.4–28.5 (CH_2_ of the dioctanoyl chain), 30.1 (-*C*H2-C≡), 31.2 (2*C*H_2_-CH_2_-CH_3_), 33.4 (*C*H_2_-C=O of chain), 33.6 (*C*H_2_-C=O of chain), 34.2 (-*C*H_2_-NH), 36.4 (C2′), 38.0 (-*C*H_2_-C=O), 51.2 (C5′), 64.1 (-*C*H_2-_O-), 68.3 (C3′), 70.8 (≡CH) 73.1 (-*C*H-O-), 79.1 (-C≡), 84.3 (C4′), 85.3 (C1′), 109.1 (C5-thymine base), 123.6 (-CH triazole), 135.3 (C6-thymine base), 144.7 (=C- triazole), 149.8 (C=O(2) thymine base), 161.8 (C=O(4) thymine base), 168.3 (-NH-*C*=O), 172.1 (-O-C=O of the octanoyl chain), 172.6 (-O-C=O of the octanoyl chain). High-resolution ESI-MS *m/z* calcd. for C_36_H_54_N_6_O_9_Na 737.3844 [M+Na]^+^, found 737.3823.

*General procedure for the synthesis of GNLs* **9a**, **9b** *and* **9c**. The above N-propargyl derivative **8a**, **8b** or **8c**, sodium ascorbate and copper sulfate were added to 1-azido-*β*-d-glucopyranoside in water/THF (1:1) mixture. The reaction mixture was maintained at 65 °C under stirring for 10 h, then cooled to room temperature and the solvent was evaporated. The residual solid was dissolved in DCM and successively washed by water and brine. The organic layer was dried over Na_2_SO_4_ and evaporated under reduced pressure. The crude product was purified by column chromatography on silica gel eluted with different ratios of methanol/dichloromethane to obtain pure click product **9a**, **9b** or **9c**.

*(Z)-4-(1-(((2R,3S,5R)-3-Hydroxy-5-(5-methyl-2,4-dioxo-3-((1-((2R,3R,4S,5S,6R)-3,4,5-trihydroxy-6-(hydroxymethyl)tetrahydro-2H-pyran-2-yl)-1H-1,2,3-triazol-4-yl)methyl)-3,4-dihydropyrimidin-1(2H)-yl)tetrahydrofuran-2-yl)methyl)-1H-1,2,3-triazol-4-ylamino)-4-oxobutane-1,2-diyl dioleate* (**9a**). *N*-propargyl derivative **8a** (160 mg, 0.16 mmol, 1.0 equiv), 1-azido-*β*-d-glucopyranoside (33 mg, 0.16 mmol, 1.0 equiv.), sodium ascorbate (16 mg, 0.2 mmol, 0.5 equiv.), copper sulfate (6.5 mg, 0.1 mmol, 0.25 equiv.), water-THF (1:1) (15 mL). Product **9a** was isolated after purification on silica gel (methanol-dichloromethane 15:85) as a white solid. Yield: 170 mg (88.1%). R*_f_*: 0.4 (methanol-dichloromethane 15:85). ^1^H-NMR (300 MHz, DMSO-*d*_6_): *δ* in ppm 0.84 (t, 6H, 2CH_3_ of the dioleyl chain, *J* = 6.0 Hz), 1.23 (s, 40H, 20CH_2_ of the dioleyl chain), 1.38–1.57 (m, 4H, 2C*H_2_*-CH_2_-C=O), 1.88 (s, 3H, CH_3_(base)), 1.90–2.07 (m, 8H, 2C*H_2_*-CH=CH-C*H_2_*), 2.15–2.30 (m, 6H, 2C*H_2_*-C=O of the dioleyl chain, 2H2′(sugar)), 2.44 (d, 2H, *J* = 6.2 Hz, C*H_2_*-C=O), 3.17–3.23 (m, 1H, -O-CH- (glucose-C5)), 3.32–3.45 (m, 4H, -C*H_2_*-OH(glucose-C6), -CH(glucose-C4), -OH(glucose (C6)), 3.65–3.77 (m, 4H, -CH(glucose-C3), H3′, -OH(C3′-sugar), -OH(glucose-C3)), 4.00-4.14 (m, 3H, -O-C*H_2_*-, -OH(glucose-C4)), 4.26–4.28 (m, 5H, -OH(glucose-C2), 2H5′, -C*H_2_*-NH-C=O), 4.56–4.72 (m, 2H, H4′, -CH(glucose-C2)), 5.05 (s, 2H, N-C*H_2_*-triazole), 5.25–5.35 (m, 5H, -C*H*-, 2C*H*=C*H* of the dioleyl chain 5.48 (d, 1H, -N-C*H*-O (glucose-C1), *J* = 9.2 Hz), 6.22 (t, 1H, H1′, *J* = 6.7 Hz), 7.49 (s, 1H, triazole-between nucleoside and glucose), 7.92 (s, 1H, triazole-between alkyl chain and nucleoside), 8.11 (s, 1H, H-6(base), 8.49 (bs, 1H, -N*H*-C=O-). ^13^C-NMR (75 MHz, DMSO-*d*_6_): *δ* in ppm 12.8 (CH_3_(base)), 14.0 (2*C*H_3_ of the dioleyl chain), 22.2 (2*C*H_2_-CH_3_), 24.4 (*C*H_2_-CH_2_-C=O), 24.5 (*C*H_2_-CH_2_-C=O), 26.6 (*C*H_2_-CH=CH-*C*H_2_), 26.7 (*C*H_2_-CH=CH-*C*H_2_), 28.5–29.2 (CH_2_ of the dioleyl chain), 31.4 (2*C*H_2_-CH_2_-CH_3_), 33.4 (*C*H_2_-C=O of chain), 33.6 (*C*H_2_-C=O of chain), 34.2 (-*C*H_2_-NH), 36.2 (N-*C*H_2_-triazole), 36.4 (C2′), 38.1 (-*C*H_2_-C=O), 51.2 (C5′), 60.7 (-*C*H_2_-OH (glucose-C6)), 64.2 (-*C*H_2-_O-), 68.3 (C3′), 69.6 (-CH(glucose-C2)), 70.8 (-CH(glucose-C4)), 71.9 (-*C*H-O-), 77.0 (-CH(glucose-C3)), 80.0 (-CH(glucose-C5)), 84.2 (-CH(glucose-C1)), 85.2 (C4′), 87.4 (C1′), 109.2 (C5-thymine base), 122.5 (-CH triazole between thymidine and alkyl chain), 123.6 (-CH triazole between glucose and thymidine), 129.6 (2-*C*H=*C*H-), 135.1 (C6-thymine base), 142.6 (=C- triazole triazole between glucose and thymidine), 144.7 (=C- triazole between thymidine and alkyl chain), 150.3 (C=O(2) thymine base), 162.3 (C=O(4) thymine base), 168.3 (-NH-*C*=O), 172.0 (-O-C=O of the oleyl chain), 172.5 (-O-C=O of oleyl chain). High-resolution FD-MS *m/z* calcd. for C_62_H_102_N_9_O_14_ 1196.7546 [M]^+^, found 1196.7449.

*4-(1-(((2R,3S,5R)-3-Hydroxy-5-(5-methyl-2,4-dioxo-3-((1-((2R,3R,4S,5S,6R)-3,4,5-trihydroxy-6-(hydroxymethyl)tetrahydro-2H-pyran-2-yl)-1H-1,2,3-triazol-4-yl)methyl)-3,4-dihydropyrimidin-1(2H)-yl)tetrahydrofuran-2-yl)methyl)-1H-1,2,3-triazol-4-ylamino)-4-oxobutane-1,2-diyl didodecanoate* (**9b**). *N*-propargyl derivative **8b** (335 mg, 0.4 mmol, 1.0 equiv), 1-azido-*β*-d-glucopyranoside (83 mg, 0.4 mmol, 1.0 equiv.), sodium ascorbate (40 mg, 0.2 mmol, 0.5 equiv.), copper sulfate (16.2 mg, 0.1 mmol, 0.25 equiv.), water/THF (1:1) (20 mL). Product **9b** was isolated after purification on silica gel (methanol-dichloromethane 15:85) as a white solid. Yield: 374 mg (89.6%). R*_f_*: 0.52 (methanol-dichloromethane 15:85). ^1^H-NMR (300 MHz, DMSO-*d*_6_): *δ* in ppm 0.85 (t, 6H, 2CH_3_ of the dilauryl chain, *J* = 6.2 Hz), 1.23 (s, 32H, 16CH_2_ of the dilauryl chain), 1.46–1.49 (m, 4H, 2C*H_2_*-CH_2_-C=O), 1.88 (s, 3H, CH_3_(base)), 2.07–2.27 (m, 6H, 2C*H_2_*-C=O of the dilauryl chain, 2H2′(sugar)), 2.45 (d, 2H, *J* = 6.6 Hz, C*H_2_*-C=O), 3.18–3.24 (m, 1H, -O-CH- (glucose-C5)), 3.33–3.46 (m, 4H, -C*H_2_*-OH(glucose-C6), -CH(glucose-C4), -OH(glucose (C6)), 3.65–3.77 (m, 3H, -CH(glucose-C3), H3′,-OH(C3′-sugar)), 4.00–4.11 (m, 3H, -O-C*H_2_*-, -OH (glucose-C3)), 4.26–4.28 (m, 4H, -OH(glucose-C4), H5′, -C*H_2_*-NH-C=O), 4.57–4.73 (m, 3H, H4′, H5″, -CH(glucose-C2)), 5.05 (s, 2H, N-C*H_2_*-triazole), 5.29–5.36 (m, 2H, -C*H*-, -OH (glucose-C2)), 5.48 (d, 1H, -N-C*H*-O (glucose-C1), *J* = 9.2 Hz), 6.23 (t, 1H, H1′, *J* = 6.8 Hz), 7.49 (s, 1H, triazole-between nucleoside and glucose), 7.92 (s, 1H, triazole-between alkyl chain and nucleoside), 8.10 (s, 1H, H-6(base), 8.48 (t,1H, -N*H*-C=O-,*J* = 5.4 Hz). ^13^C-NMR (75 MHz, DMSO-*d*_6_): *δ* in ppm 12.7 (CH_3_(base)), 13.9 (2*C*H_3_ of the dilauryl chain), 22.1 (2*C*H_2_-CH_3_), 24.4 (*C*H_2_-CH_2_-C=O), 24.5 (*C*H_2_-CH_2_-C=O), 28.4-29.1 (CH_2_ of the dilauryl chain), 31.3 (2*C*H_2_-CH_2_-CH_3_), 33.4 (*C*H_2_-C=O of chain), 33.6 (*C*H_2_-C=O of chain), 34.1 (-*C*H_2_-NH), 36.1 (N-*C*H2-triazole), 36.4 (C2′), 38.1 (-*C*H_2_-C=O), 51.1 (C5′), 60.7 (-*C*H_2_-OH (glucose-C6)), 64.1 (-*C*H_2-_O-), 68.3 (C3′), 69.5 (-CH(glucose-C2)), 70.7 (-CH(glucose-C4)), 71.9 (-*C*H-O-), 77.0 (-CH(glucose-C3)), 80.0 (-CH(glucose-C5)), 84.2 (-CH(glucose-C1)), 85.1 (C4′), 87.4 (C1′), 109.1 (C5-thymine base), 122.5 (-CH triazole between thymidine and alkyl chain), 123.5 (-CH triazole between glucose and thymidine), 135.1 (C6-thymine base), 142.6 (=C- triazole triazole between glucose and thymidine), 144.6 (=C- triazole between thymidine and alkyl chain), 150.3 (C=O(2) thymine base), 162.3 (C=O(4) thymine base), 168.3 (-NH-*C*=O), 172.0 (-O-C=O of the lauryl chain), 172.5 (-O-C=O of the lauryl chain). High-resolution ESI-MS *m/z* calcd. for C_50_H_81_N_9_O_14_Na 1054.5795 [M+Na]^+^, found 1054.5854.

*4-(1-(((2R,3S,5R)-3-Hydroxy-5-(5-methyl-2,4-dioxo-3-((1-((2R,3R,4S,5S,6R)-3,4,5-trihydroxy-6-(hydroxymethyl)tetrahydro-2H-pyran-2-yl)-1H-1,2,3-triazol-4-yl)methyl)-3,4-dihydropyrimidin-1(2H)-yl)tetrahydrofuran-2-yl)methyl)-1H-1,2,3-triazol-4-ylamino)-4-oxobutane-1,2-diyl dioctanoate* (**9c**). *N*-propargyl derivative **8c** (490 mg, 0.7 mmol, 1.0 equiv.), 1-azido-*β*-d-glucopyranoside (141 mg, 0.7 mmol, 1.0 equiv.), sodium ascorbate (28 mg, 0.14 mmol, 0.2 equiv.), copper sulfate (11 mg, 0.07 mmol, 0.1 equiv.), water/THF (1:1) (20 mL). Product **9c** was isolated after purification on silica gel (methanol-dichloromethane 15:85) as a white solid. Yield: 530 mg (84.1%). R*_f_*: 0.46 (methanol-dichloromethane 15:85). ^1^H-NMR (300 MHz, DMSO-*d*_6_): *δ* in ppm 0.85 (t, 6H, 2CH_3_ of the dioctanoyl chain, *J* = 6.2 Hz), 1.23 (s, 32H, 16CH_2_ of the dioctanoyl chain), 1.36–1.60 (m, 4H, 2C*H_2_*-CH_2_-C=O), 1.88 (s, 3H, CH_3_(base)), 2.16–2.28 (m, 6H, 2C*H_2_*-C=O of the dioctanoyl chain, 2H2′(sugar)), 2.45 (d, 2H, *J* = 6.8 Hz, C*H_2_*-C=O), 3.17–3.25 (m, 1H, -O-CH- (glucose-C5)), 3.32–3.47 (m, 3H, -C*H_2_*-OH(glucose-C6), -CH(glucose-C4), 3.64–3.78 (m, 2H, -CH(glucose-C3, H3′), 4.01–4.12 (m, 2H, -O-C*H_2_*-), 4.23–4.28 (m, 4H, -OH(glucose-C4), H5′, -C*H_2_*-NH-C=O,), 4.57–4.73 (m, 3H, H4′, H5″,-CH(glucose-C2)), 5.05 (s, 2H, N-C*H_2_*-triazole), 5.14 (d, 1H, -OH(glucose (C6), *J* = 5.3 Hz), 5.24 (d, 1H, -OH (glucose-C3), *J* = 4.7 Hz), 5.29–5.31 (m, 1H, -C*H*-), 5.34 (d, 1H, -OH (glucose-C2), *J* = 6.0 Hz) 5.48 (d, 1H, -N-C*H*-O (glucose-C1), *J* = 9.2 Hz), 5.53 (d, 1H, -OH(C3′-sugar), *J* = 4.2 Hz) 6.23 (t, 1H, H1′, *J* = 6.8 Hz), 7.49 (s, 1H, triazole-between nucleoside and glucose), 7.91 (s, 1H, triazole-between alkyl chain and nucleoside), 8.10 (s, 1H, H-6(base), 8.48 (t, 1H, -N*H*-C=O-, *J* = 5.4 Hz). ^13^C-NMR (75 MHz, DMSO-*d*_6_): *δ* in ppm 12.7 (CH_3_(base)), 13.9 (2*C*H_3_ of the dioctanoyl chain), 22.1 (2*C*H_2_-CH_3_), 24.4 (*C*H_2_-CH_2_-C=O), 24.5 (*C*H_2_-CH_2_-C=O), 28.3–28.4 (CH_2_ of the dioctanoyl chain), 31.1 (2*C*H_2_-CH_2_-CH_3_), 33.4 (*C*H_2_-C=O of chain), 33.6 (*C*H_2_-C=O of chain), 34.1 (-*C*H_2_-NH), 36.1 (N-*C*H2-triazole), 36.4 (C2′), 38.1 (-*C*H_2_-C=O), 51.1 (C5′), 60.7 (-*C*H_2_-OH (glucose-C6)), 64.1 (-*C*H_2-_O-), 68.3 (C3′), 69.5 (-CH(glucose-C2)), 70.7 (-CH(glucose-C4)), 71.9 (-*C*H-O-), 77.0 (-CH(glucose-C3)), 80.0 (-CH(glucose-C5)), 84.2 (-CH(glucose-C1)), 85.2 (C4′), 87.4 (C1′), 109.1 (C5-thymine base), 122.5 (-CH triazole between glucose and thymidine), 123.6 (-CH triazole between thymidine and alkyl chain), 135.1 (C6-thymine base), 142.6 (=C- triazole triazole between glucose and thymidine), 144.7 (=C- triazole between thymidine and alkyl chain), 150.3 (C=O(2) thymine base), 162.3 (C=O(4) thymine base), 168.3 (-NH-*C*=O), 172.1 (-O-C=O of the dioctanoyl chain), 172.6 (-O-C=O of the dioctanoyl chain). High-resolution ESI-MS *m/z* calcd. for C_42_H_65_N_9_O_14_Na 942.4543 [M+Na]^+^, found 942.4562.

*1-((2R,4S,5R)-5-((tert-Butyldimethylsilyloxy)methyl)-4-hydroxytetrahydrofuran-2-yl)-5-methylpyrimidine-2,4(1H,3H)-dione* (**11**). To a solution of thymidine **10** (5 g, 20.6 mmol, 1.0 equiv) in pyridine (125 mL) was added DMAP (0.126 g, 1.03 mmol, 0.05 equiv.) and TBDMS-Cl (3.48 g, 23.1 mmol, 1.1 equiv.) sequentially. The reaction mixture was stirred overnight at room temperature. After removal of the solvent under reduced pressure, the crude reaction mixture was dissolved in DCM and successively washed with water, aqueous NaHCO_3_ solution (5%) and brine. The organic layer was dried over Na_2_SO_4_ and evaporated under reduced pressure. The crude product was purified by column chromatography on silica gel (ethyl acetate-hexane-TEA (79:20:1)) to give pure **11** as a white solid. Yield: 5.4 g (73.5%). R*_f_*: 0.45 (ethyl acetate-hexane-TEA 79:20:1). The data agreed with the literature values [41,42]. ^1^H-NMR (300 MHz, CDCl_3_): *δ* in ppm 0.1 (s, 6H, -Si(CH_3_)_2_), 0.91 (s, 9H, -Si-C(CH_3_)_2_), 1.90 (s, 3H, CH_3_(base)), 2.04-2.12 (m, 1H, H2'(sugar)), 2.35-2.42 (m, 1H, H2ʺ(sugar)), 3.12 (bs, 1H,-OH(sugar)), 3.80–3.92 (m, 2H, 2H5'(sugar)), 4.02–4.11 (m, 1H, H3'(sugar)), 4.44 (bs, 1H, H4'(sugar)), 6.37–6.42 (m, 1H, 1H'(sugar)), 7.53 (s, 1H, H-6(base)), 9.39 (s, 1H, -NH(base)).

*1-((2R,4S,5R)-5-((tert-Butyldimethylsilyloxy)methyl)-4-(prop-2-ynyloxy)tetrahydrofuran-2-yl)-5-methylpyrimidine- 2,4(1H,3H)-dione* (**12**). Anhydrous THF (100 mL) was added under nitrogen to TBDMS protected thymidine **11** (5 g, 14.03 mmol, 1 equiv.). The mixture was cooled at 0 °C and NaH (1.4 g (60% in oil), 35.06 mmol, 2.5 equiv.) was added by small portion. Propargyl bromide (4.69 g, 3.4 mL (80 wt.% solution in toluene), 31.55 mmol, 2.25 equiv) was then added at room temperature. The reaction mixture was maintained at 50 °C under stirring for 24 h. The reaction was stopped by addition of methanol (20 mL). The solvent was removed under reduced pressure. The residual mixture was dissolved in dichloromethane and successively washed by water and brine. The organic layer then dried over Na_2_SO_4_ and evaporated under reduced pressure. The crude product was purified by column chromatography on silica gel (ethyl acetate-hexane-TEA 39:60:1 to 49:50:1). Product **12** was isolated as a colourless oil. Yield: 4.95 g (89.5%). R*_f_*: 0.44 (ethyl acetate-hexane-TEA (49:50:1)). The data agreed with the literature values [39]. ^1^H-NMR (300 MHz, CDCl_3_): *δ* in ppm 0.09 (s, 6H, -Si(CH_3_)_2_), 0.89 (s, 9H, -Si-C(CH_3_)_2_), 1.88 (s, 3H, CH_3_(base)), 1.92–2.01 (m, 1H, H2'(sugar)), 2.40–2.47 (m, 2H, H2ʺ(sugar), -C≡C*H*), 3.75–3.90 (m, 2H, 2H5'), 4.07–4.24 (m, 3H, H3'(sugar), O-C*H_2_*-C≡), 4.31–4.33 (m, 1H, H4'(sugar)), 6.25–6.29 (m, 1H, 1H'(sugar)), 7.48 (s, 1H, H-6(base)), 9.76 (s, 1H, -NH(base)). ^13^C-NMR (75 MHz, CDCl_3_): *δ* in ppm −5.56 (-Si-*C*H_3_), −5.5 (-Si-*C*H_3_), 12.4 (CH_3_(base)), 18.2 (-*C*-(CH_3_)_3_), 25.8 (-C-(*C*H_3_)_3_), 37.5 (C2′), 56.2 (-O-*C*H_2_-C≡), 63.4 (C5'), 75.0 (≡*C*H), 78.4 (C3'), 79.0 (-C≡), 84.7 (C4'), 84.8 (C1'), 110.6 (C5-thymine base), 135.2 (C6-thymine base), 150.5 (C=O(2) thymine base), 164.1 (C=O(4) thymine base). High-resolution ESI-MS *m/z* calcd. for C_19_H_30_N_2_O_5_NaSi 417.1816 [M+Na]^+^, found. 417.1834.

*1-((2R,4S,5R)-5-(Hydroxymethyl)-4-(prop-2-ynyloxy)tetrahydrofuran-2-yl)-5-methylpyrimidine-2,4(1H,3H)-dione* (**13**). To a mixture of compound **12** (4.85 g, 12.3 mmol, 1.0 equiv.) in THF (40 mL) was added 1M solution of TBAF (14.7 mL, 14.75 mmol, 1.2 equiv.) in THF and the resulting mixture was stirred at room temperature for 1h. After removal of the solvent under reduced pressure, the crude deprotected product was applied directly to a silica gel chromatographic column (methanol-ethyl acetate 0:100 to 5:95). Product **13** was isolated as a white solid. Yield: 3.2 g (93.0%). R*_f_*: 0.55 (ethyl acetate). ^1^H-NMR (300 MHz, DMSO-*d*_6_): *δ* in ppm 1.77 (s, 3H, CH_3_(base)), 2.04–2.33 (m, 2H, H2'(sugar)), 3.47 (s, -C≡C*H*), 3.58 (bs 2H, 2H5'), 3.93 (bs, 1H, H3'(sugar)), 4.21 (bs, 3H, - OH, O-C*H_2_*-C≡), 5.15(s, 1H, H4'(sugar)), 6.10 (bs, 1H, 1H'(sugar)), 7.70 (s, 1H, H-6(base)), 11.34 (s, 1H, -NH(base)). ^13^C-NMR (75 MHz, DMSO-*d*_6_): *δ* in ppm 12.4 (CH_3_(base)), 36.1 (C2′), 55.9 (-O-*C*H_2_-C≡), 61.6 (C5'), 77.4 (≡*C*H), 78.8 (C3'), 80.3 (-C≡), 83.9 (C4'), 84.5 (C1'), 109.7 (C5-thymine base), 136.1 (C6-thymine base), 150.6 (C=O(2) thymine base), 163.9 (C=O(4) thymine base). High-resolution ESI-MS *m/z* calcd. for C_13_H_16_N_2_O_5_Na 303.0951 [M+Na]^+^, found. 303.0945.

*(2R,3R,4S,5R,6R)-2-(Acetoxymethyl)-6-(4-(((2R,3S,5R)-2-(hydroxymethyl)-5-(5-methyl-2,4-dioxo-3,4-dihydropyrimidin-1(2H)-yl)tetrahydrofuran-3-yloxy)methyl)-1H-1,2,3-triazol-1-yl)tetrahydro-2H-pyran-3,4,5-triyl triacetate* (**14**). 1-Azido-2,3,4,6-tetra-*O*-acetyl-*β*-d-glucopyranoside (2.5 g, 6.69 mmol, 1 equiv.), sodium ascorbate (265.5 mg, 1.34 mmol, 0.2 equiv.) and copper sulfate (106.8 mg, 0.66 mmol, 0.1 equiv.) were added to compound **13** (1.87 g, 6.69 mmol, 1 equiv.) in water/THF (1:1) mixture (100 mL). The reaction mixture was maintained at 65 °C under stirring for 10 h. The mixture was cooled down to room temperature. After removal of the solvent under reduced pressure, the crude product was applied directly to a silica gel chromatographic column (methanol-ethyl acetate 0:100 to 5:95). Product **14** was isolated as a white solid (foam). Yield: 4.24 g (97.1%). R*_f_*: 0.25 (ethyl acetate). ^1^H-NMR (300 MHz, CDCl_3_): *δ* in ppm 1.88 (s, 3H, CH_3_(base)), 1.89–2.08 (m, 12H, 4CH_3_-C=O(glucose)), 2.24–2.33 (m, 1H, H2'(sugar)), 2.37–2.45 (m, 1H, H2ʺ(sugar), 3.31 (bs, 1H, -OH), 3.73–4.17 (m, 5H, 2H5'(sugar), H3'(sugar), O-C*H_2_*-triazole), 4.25–4.34 (m, 2H, H4'(sugar), -O-C*H*(glucose-C5)), 4.68 (q, 2H, -C*H_2_*-OAc(glucose), *J* = 12.7 Hz), 5.25 (t, 1H, -CH(glucose-C4), *J* = 9.5 Hz), 5.34-5.48 (m, 2H, 2-CH(glucose-C2, C3)), 5.88 (d, 1H, -N-C*H*-O-(glucose), *J* = 8.85 Hz), 6.15 (t, 1H, 1H'(sugar), *J* = 6.8 Hz), 7.46 (s, 1H, triazole), 7.86 (s, 1H, H-6(base)), 9.10 (s, 1H, -NH(base)). ^13^C-NMR (75 MHz, CDCl_3_): *δ* in ppm 12.4 (CH_3_(base)), 20.1–20.6 (4 -CH_3_ (acetyl)), 36.1 (C2′), 61.4 (-O-*C*H_2_-triazole), 62.0 (C5'), 62.2 (-*C*H_2_-OAc), 67.5 (-CH (glucose-C3)), 70.5 (-CH (glucose-C2)), 72.3 (-CH (glucose-C4)), 74.9 (-CH (glucose-C5)), 78.0 (-CH (glucose-C1)), 84.9 (C3'), 85.5 (C4'), 86.0 (C1'), 110.8 (C5-thymine base), 121.4 (-CH triazole), 136.7 (C6-thymine base), 145.2 (=C- triazole), 150.5 (C=O(2) thymine base), 164.1 (C=O(4) thymine base), 169.3–169.9 (3 C=O(acetyl at C2, C3, C4)), 170.5 (-CH_2_-*C*=O(acetyl at C6)). High-resolution ESI-MS *m/z* calcd. for C_27_H_35_N_5_O_14_Na 676.2072 [M+Na]^+^, found. 676.2089.

*(2R,3R,4S,5R,6R)-2-(Acetoxymethyl)-6-(4-(((2R,3S,5R)-2-(azidomethyl)-5-(5-methyl-2,4-dioxo-3,4-dihydropyrimidin-1(2H)-yl)tetrahydrofuran-3-yloxy)methyl)-1H-1,2,3-triazol-1-yl)tetrahydro-2H-pyran-3,4,5-triyl triacetate* (**15**). Anhydrous pyridine (80 mL) was added to click product **14** (4 g,6.12 mmol, 1 equiv.). The mixture was cooled to 0 °C and methanesulfonyl chloride (0.92 g, 0.62 mL, 7.95 mmol, 1.3 equiv.) was added dropwise. The mixture was stirred at room temperature for 4 h. The solvent was evaporated under reduced pressure and the residual compound was used directly in the following step without further purification. To the above crude product, DMF (80 mL) and sodium azide (1.99 g, 30.6 mmol, 5 equiv) were added. The reaction mixture was maintained at 80 °C under stirring for 4 h. DMF was removed under reduced pressure. The residual solid was dissolved in ethyl acetate and washed successively twice with aqueous 5% NaHCO_3_ solution and once with brine. The organic phase was dried on Na_2_SO_4_ and evaporated under reduced pressure. The crude product was purified by column chromatography on silica gel (ethyl acetate). Product **15** was isolated as a white solid (foam). Yield: 3.85 g (92.7%). R*_f_*: 0.45 (ethyl acetate). ^1^H NMR (300 MHz, CDCl_3_): *δ* in ppm 1.88 (s, 3H, CH_3_(base)), 1.92–2.08 (m, 12H, 4CH_3_-C=O(glucose)), 2.11–2.15 (m, 1H, H2'(sugar)), 2.38–2.46 (m, 1H, H2ʺ(sugar), 3.54–3.73 (m, 2H, 2H5'(sugar), 3.99–4.19 (m, 4H, H3'(sugar), O-C*H_2_*-triazol, -O-C*H*(glucose-C5)), 4.28–4.34 (m, 1H, H4'(sugar), 4.66 (q, 2H, -C*H_2_*-OAc(glucose), *J* = 12.2 Hz), 5.24 (t, 1H, -CH(glucose-C4), *J* = 9.5 Hz), 5.35–5.48 (m, 2H, 2-CH(glucose-C2, C3)), 5.88 (d, 1H, -N-C*H*-O-(glucose), *J* = 8.86 Hz), 6.23–6.28 (m, 1H, 1H'(sugar)), 7.32 (s, 1H, triazole), 7.83 (s, 1H, H-6(base)), 8.95 (s, 1H, -NH(base)). ^13^C-NMR (75 MHz, CDCl_3_): *δ* in ppm 12.5 (CH_3_(base)), 20.0-20.6 (4 -CH_3_ (acetyl)), 36.9 (C2′), 52.3 (C5'), 61.4 (-O-*C*H_2_-triazole), 62.5 (-*C*H_2_-OAc), 67.5 (-CH (glucose-C3)), 70.4 (-CH (glucose-C2)), 72.2 (-CH (glucose-C4)), 74.9 (-CH (glucose-C5)), 78.5 (-CH (glucose-C1)), 82.3 (C3'), 84.8 (C4'), 85.6 (C1'), 111.3 (C5-thymine base), 121.2 (-CH triazole), 135.3 (C6-thymine base), 145.0 (=C- triazole), 150.3 (C=O(2) thymine base), 163.8 (C=O(4) thymine base), 168.9–169.8 (3 C=O(acetyl at C2, C3, C4)), 170.4 (-CH_2_-*C*=O(acetyl at C6)). High-resolution ESI-MS *m/z* calcd. for C_27_H_34_N_8_O_13_Na 701.2137 [M+Na]^+^, found. 701.2139.

*(2R,3R,4S,5R,6R)-2-(Acetoxymethyl)-6-(4-(((2R,3S,5R)-2-((4-((4,4,5,5,6,6,7,7,8,8,9,9,10,10,11,11,11-heptadecafluoroundecanamido)methyl)-1H-1,2,3-triazol-1-yl)methyl)-5-(5-methyl-2,4-dioxo-3,4-dihydropyrimidin-1(2H)-yl)tetrahydrofuran-3-yloxy)methyl)-1H-1,2,3-triazol-1-yl)tetrahydro-2H-pyran-3,4,5-triyl triacetate* (**16**). *N*-propargyl-1*H*,1*H*,2*H*,2*H*-perfluoroundecanoyl amide [29] (480 mg, 0.91 mmol, 1 equiv.), sodium ascorbate (36 mg, 0.18 mmol, 0.2 equiv.) and copper sulfate (14.5 mg, 0.091 mmol, 0.1 equiv.) were added to azido derivative **15** (616 mg, 0.91 mmol, 1 equiv.) in water/THF (1:1) mixture (50 mL). The reaction mixture was maintained at 65 °C under stirring for 10 h. The mixture was cooled down to room temperature. After removal of the solvent under reduced pressure, the crude product applied directly to a silica gel chromatographic column (methanol-ethyl acetate 0:100 to 5:95). Product **16** was isolated as a white sticky foam. Yield: 1.03 g (94.4%). R*_f_*: 0.2 (ethyl acetate). ^1^H-NMR (300 MHz, CDCl_3_): *δ* in ppm 1.69 (s, 3H, CH_3_(base)), 1.80, 2.00 and 2.11 (s, 12H, 4CH_3_-C=O(glucose)), 2.13–2.73 (m, 6H, 2H2'(sugar), CF_2_-C*H_2_*-C*H_2_*-C=O), 4.07–4.45 (m, 6H, H5≡(sugar), -C*H_2_*-OAc(glucose-C6), -O-C*H*(glucose-C5), triazole-CH'*H*≡-NH-C=O, H3'(sugar)), 4.61–4.70 (m, 3H, O-C*H_2_*-triazole, H5'(sugar)), 4.80–4.89 (m, 2H, H4'(sugar), triazole-C*H'*Hʺ-NH-C=O), 5.40–5.51 (m, 2H, 2-CH(glucose-C3, C4)), 5.69–5.78 (m, 1H, -CH(glucose-C2)), 5.82–5.90 (m, 1H-N-C*H*-O-(glucose-C1)), 6.22–6.27 (m, 1H, 1H'(sugar)), 6.54 (s, 1H, triazole-between nucleoside and glucose), 7.35 (bs, 1H, -N*H*-C=O-), 7.64 (s, 1H, triazole-between F-chain and nucleoside) 8.35 (s, 1H, H-6(base)), 9.27 (s, 1H, -NH(base)). ^13^C-NMR (75 MHz, CDCl_3_): *δ* in ppm 11.9 (CH_3_(base)), 20.0–20.6 (4 -CH_3_ (acetyl)), 26.5 (-CH_2_-CH_2_-chain) 34.2 (triazole-*C*H_2_-NH) 35.2 (C2′), 49.8 (C5'), 61.5 (-O-*C*H_2_-triazole), 63.2 (-*C*H_2_-OAc), 67.8 (-CH (glucose-C3)), 70.8 (-CH (glucose-C2)), 72.2 (-CH (glucose-C4)), 74.2 (-CH (glucose-C5)), 75.1 (-CH (glucose-C1)), 81.0 (C3'), 84.6 (C4'), 85.9 (C1'), 111.2 (C5-thymine base), 121.9 (-CH triazole-between F-chain and nucleoside), 124.3 (-CH triazole-between nucleoside and glucose) 136.3 (C6-thymine base), 144.4 (=C- triazole-between nucleoside and glucose), 144.5 (=C- triazole-between F-chain and nucleoside), 150.3 (C=O(2) thymine base), 163.7 (C=O(4) thymine base), 169.1–170.2 (3 C=O(acetyl at C2, C3, C4)), 170.3 (-CH_2_-*C*=O(acetyl at C6)), 170.6 (-NH-*C*=O). High-resolution FD-MS *m/z* calcd. for C_41_H_42_F_17_N_9_O_14_ 1207.2580, found. 1207.2582.

*4,4,5,5,6,6,7,7,8,8,9,9,10,10,11,11,11-Heptadecafluoro-N-((1-(((2R,3S,5R)-5-(5-methyl-2,4-dioxo-3,4-dihydropyrimidin-1(2H)-yl)-3-((1-((2R,3R,4S,5S,6R)-3,4,5-trihydroxy-6-(hydroxymethyl)tetrahydro-2H-pyran-2-yl)-1H-1,2,3-triazol-4-yl)methoxy)tetrahydrofuran-2-yl)methyl)-1H-1,2,3-triazol-4-yl)methyl) undecanamide* (**17**). To a solution of double click product **16** (950 mg, 0.79 mmol, 1.0 equiv.) in methanol (40 mL) was added sodium methoxide powder (850 mg, 15.74 mmol, 20 equiv.) and the resulting mixture was stirred at room temperature for 2 h. After removal of the solvent under reduced pressure, the crude deprotected product was applied directly to a silica gel chromatographic column (methanol-dichloromethane 1:4). Product **17** was isolated as a white solid. Yield: 762 m g (93.2%). R*_f_*: 0.35 (methanol-dichloromethane 1:4). ^1^H-NMR (300 MHz, DMSO-*d*_6_): *δ* in ppm 1.80 (s, 3H, CH_3_(base)), 2.20–2.33 (m, 2H, 2H2'(sugar)), 2.43–2.56 (m, 4H, CF_2_-C*H_2_*-C*H_2_*-C=O), 3.15–3.26 (m, 1H, -O-CH- (glucose-C5)), 3.40–3.45 (m, 3H, -C*H_2_*-OH(glucose-C6), -CH(glucose-C4), 3.63–3.80 (m, 2H, -CH(glucose-C3), H3′), 4.20–4.40 (m, 4H, -OH (glucose-C4), H5′, -C*H_2_*-NH-C=O,), 4.58 (s, 2H, -O-C*H_2_*-triazole), 4.63–4.76 (m, 3H, H4′, H5″, -CH(glucose-C2)), 5.19 (d, 1H, -OH(glucose (C6), *J* = 5.5 Hz), 5.33 (d, 1H, -OH (glucose-C3), *J* = 4.7 Hz), 5.44 (d, 1H, -OH (glucose-C2), *J* = 6.0 Hz), 5.53 (d, 1H, -N-C*H*-O (glucose-C1), *J* = 9.4 Hz), 6.12 (t, 1H, 1H'(sugar), *J* = 7.3 Hz), 7.45 (s, 1H, triazole-between nucleoside and glucose), 7.98 (s, 1H, triazole-between F-chain and nucleoside) 8.33 (s, 1H, H-6(base)), 8.57 (t, 1H, -N*H*-C=O-, *J* = 5.46 Hz), 11.37 (s, 1H, -NH(base)). ^13^C-NMR (75 MHz, DMSO-*d*_6_): *δ* in ppm 12.0 (CH_3_(base)), 25.7–25.8 (-CH_2_-CH_2_-chain) 34.4 (triazole-*C*H_2_-NH) 35.1 (C2′), 51.4 (C5'), 60.8 (-O-*C*H_2_-triazole), 62.1 (-*C*H_2_-OH (glucose-C6)), 69.6 (-CH(glucose-C2)), 72.1 (-CH(glucose-C4)), 77.0 (-CH(glucose-C3)), 79.1 (C3′), 80.0 (-CH(glucose-C5)), 81.6 (-CH(glucose-C1)), 84.5 (C4′), 87.5 (C1′), 109.9 (C5-thymine base), 123.3 (-CH triazole between thymidine and alkyl chain), 123.6 (-CH triazole between glucose and thymidine), 136.1 (C6-thymine base), 143.5 (=C- triazole triazole between glucose and thymidine), 144.7 (=C- triazole between thymidine and alkyl chain), 150.4 (C=O(2) thymine base), 163.7 (C=O(4) thymine base), 169.3 (-NH-*C*=O). MS *m/z* calcd. for C_33_H_34_F_17_N_9_O_10_Na 1062.6, found. 1062.3.

## 4. Conclusions

The objectives of the present investigation were to expand the current repertoire of glycosyl-nucleolipids (GNLs) and study the effect of the structural modifications on their physicochemical properties. In this contribution, two synthetic routes to a series of i) double chain hydroxybutanamide based GNLs and ii) second generation GNF are described. Thermal experiments show that hydroxybutanamide amphiphiles exhibit endothermic phase transitions at temperatures of 51.9 ± 1.8 °C, 51.7 ± 1.8 °C, 56.1 ± 2.4 °C and −16.3 ± 2.4 °C for C_8_, C_12_, C_18_ and oleyl double chains analogues, respectively. In the case of GNFs, surface tension measurements show critical aggregation concentrations (CACs) of 11 µM for both 1st GNF and 2nd GNF, indicating that the polar head structure has a poor impact on the CAC values. The 2nd GNF features a better surface activity (γ_lim_) compared to the 1st GNF. The synthetic strategies developed in this report will be used in the future for the design of new glycosyl-nucleolipids that self-assemble into soft materials for biomedical and tissue engineering applications.

## Supplementary Materials

Supplementary materials can be accessed at: http://www.mdpi.com/1420-3049/18/10/12241/s1.
