# Supplementary material for: Glycosyl-Nucleolipids as New Bioinspired Amphiphiles
Source: Molecules. 2013 Sep 30;18(10):12241–63. doi: 10.3390/molecules181012241 (PMC6270249; doi:10.3390/molecules181012241)
Supplement: Supplementary file 1 [file molecules-18-12241-s001.pdf]

Supplementary Materials

I. NMR Spectra

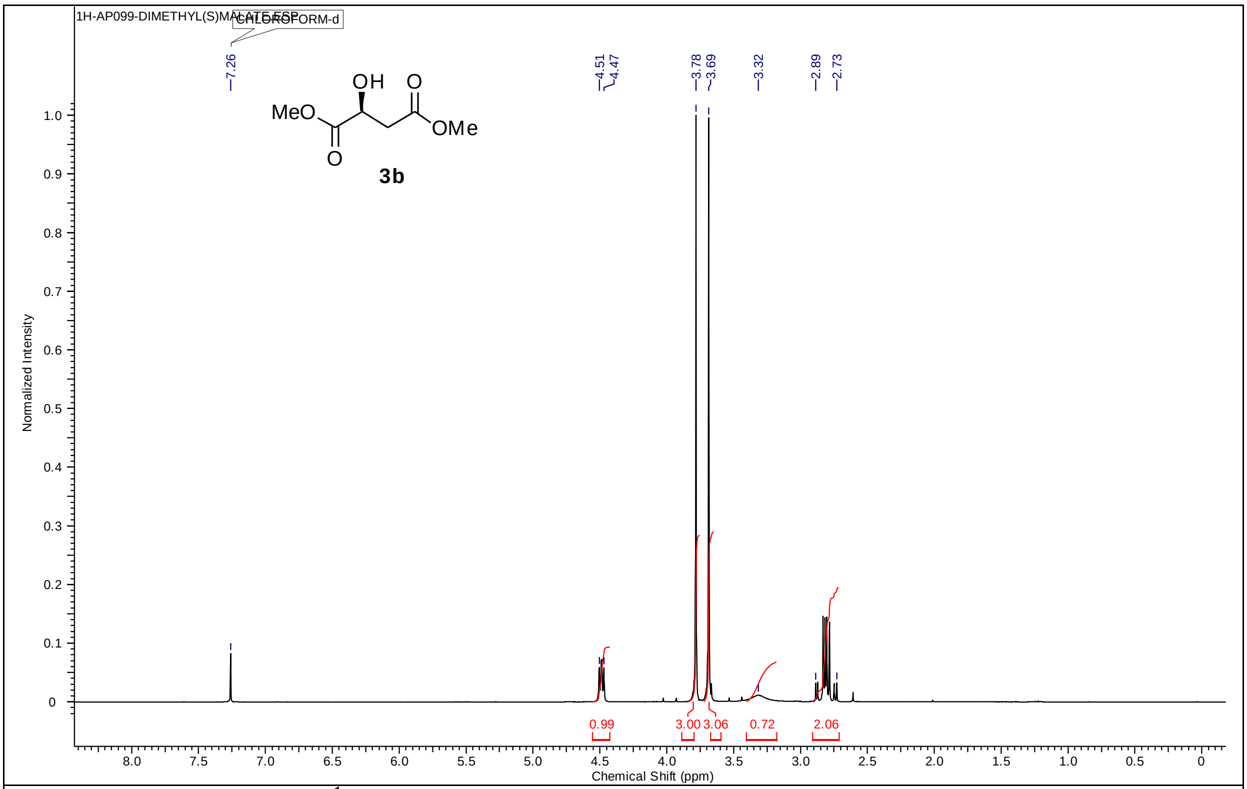

^{1}H-NMR spectrum of dimethyl (*S*)-malate **3b**

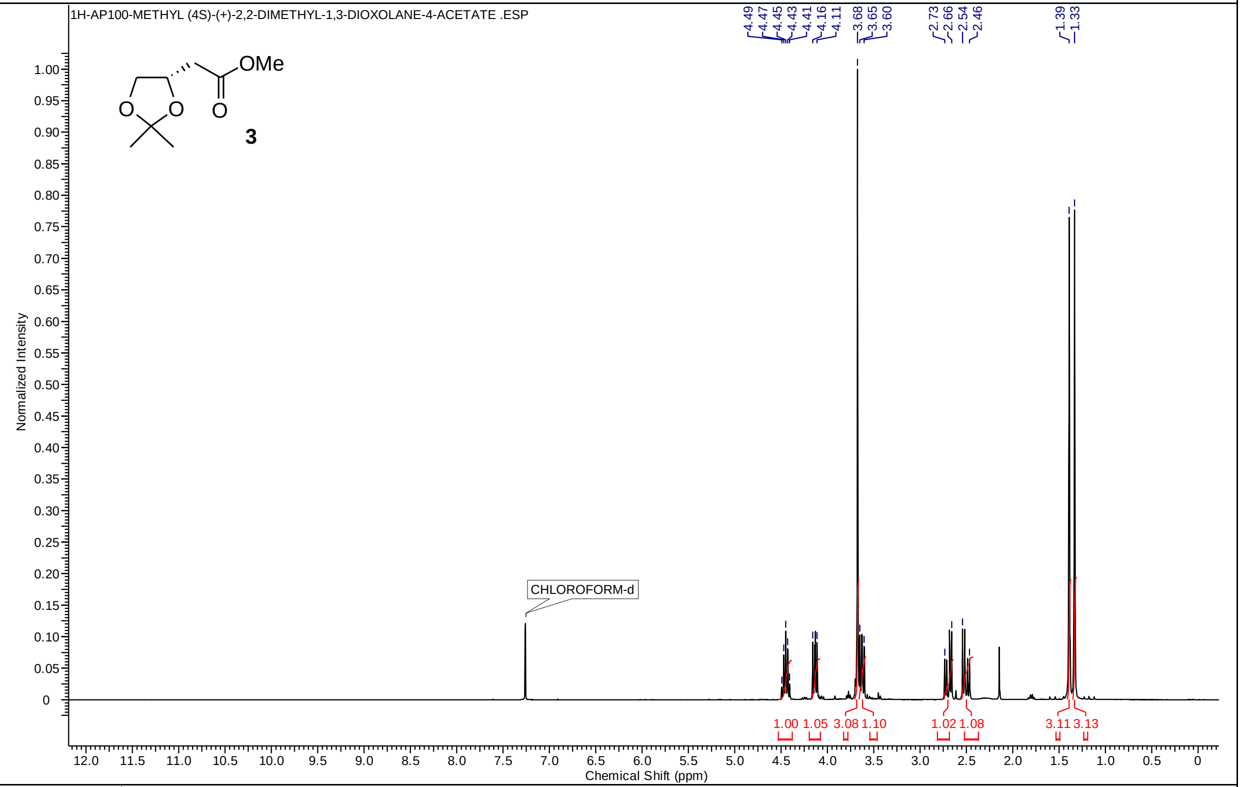

^{1}H-NMR spectrum of Methyl (4S)-(+)-2,2-dimethyl-1,3-dioxolane-4-acetate **3**

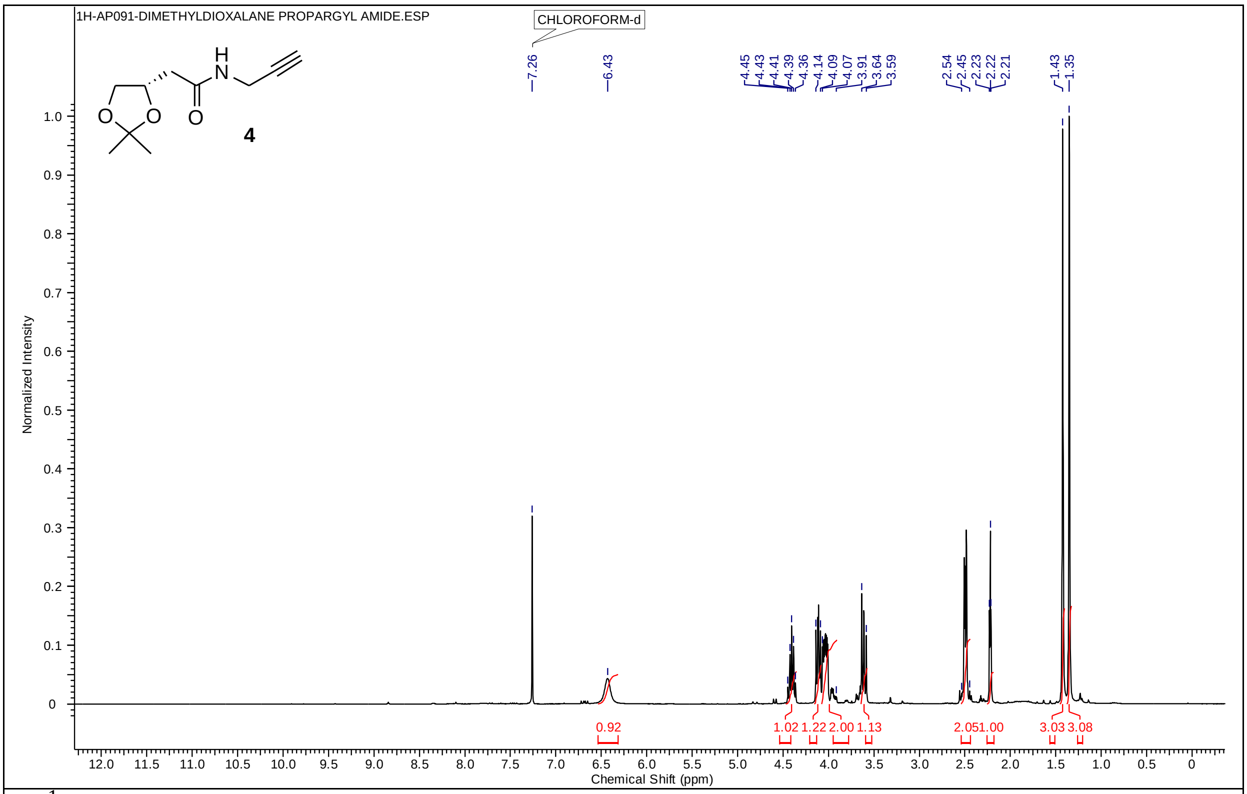

^{1}H-NMR spectrum of (*S*)-2-(2,2-dimethyl-1,3-dioxolan-4-yl)-*N*-(prop-2-ynyl)acetamide **4**

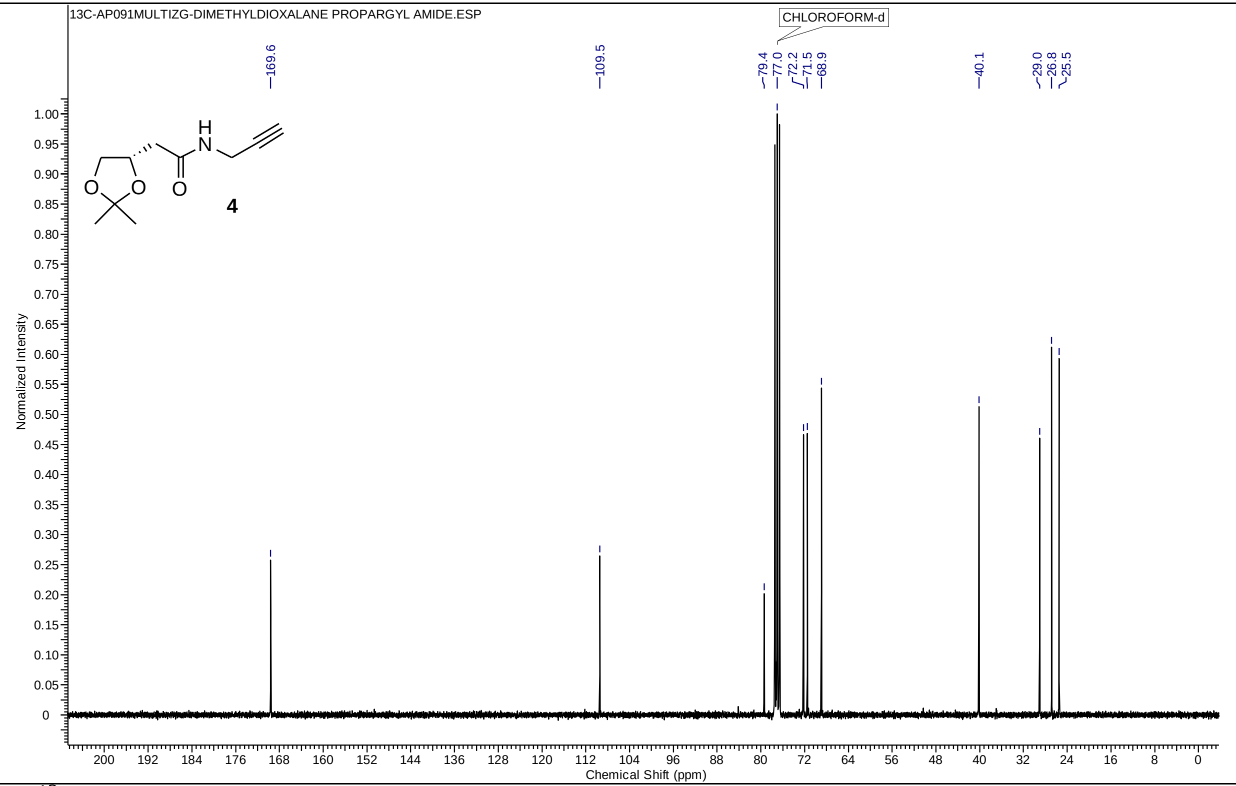

^{13}C-NMR spectrum of (*S*)-2-(2,2-dimethyl-1,3-dioxolan-4-yl)-*N*-(prop-2-ynyl)acetamide **4**

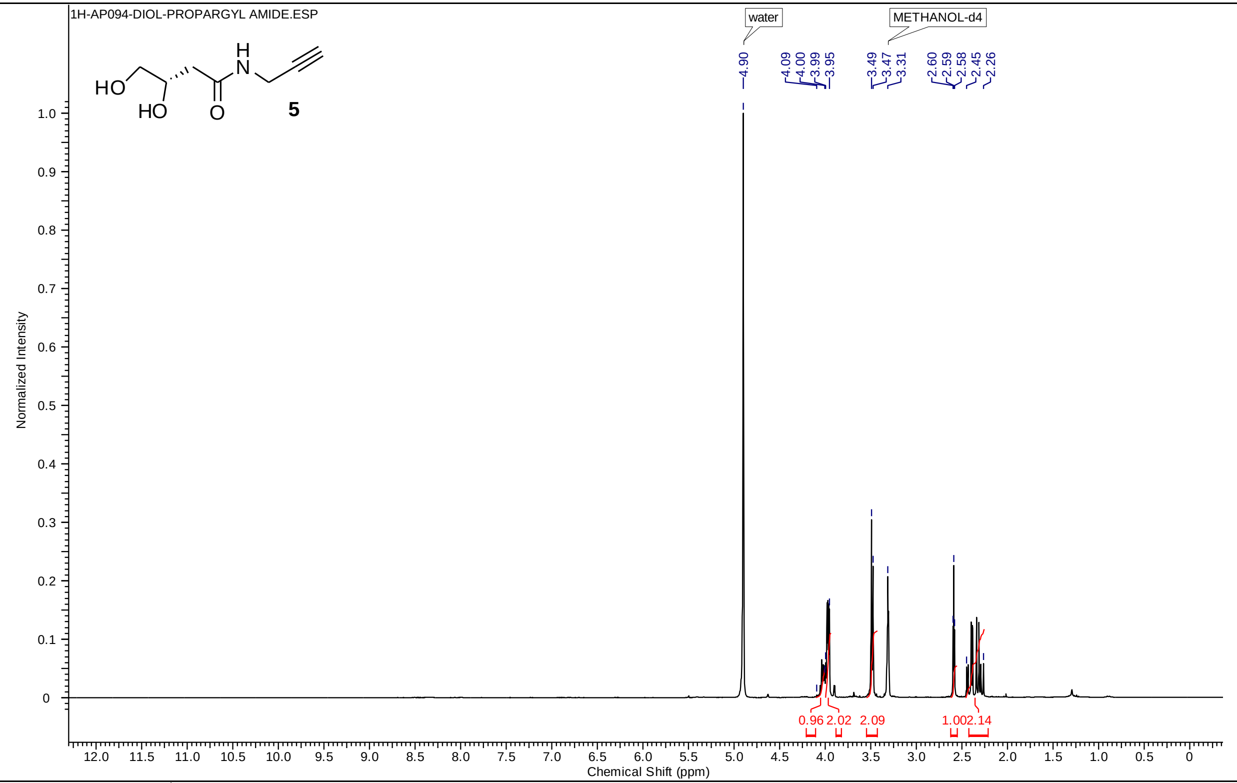

^{1}H-NMR spectrum of (*S*)-3,4-dihydroxy-*N*-(prop-2-ynyl)butanamide **5**

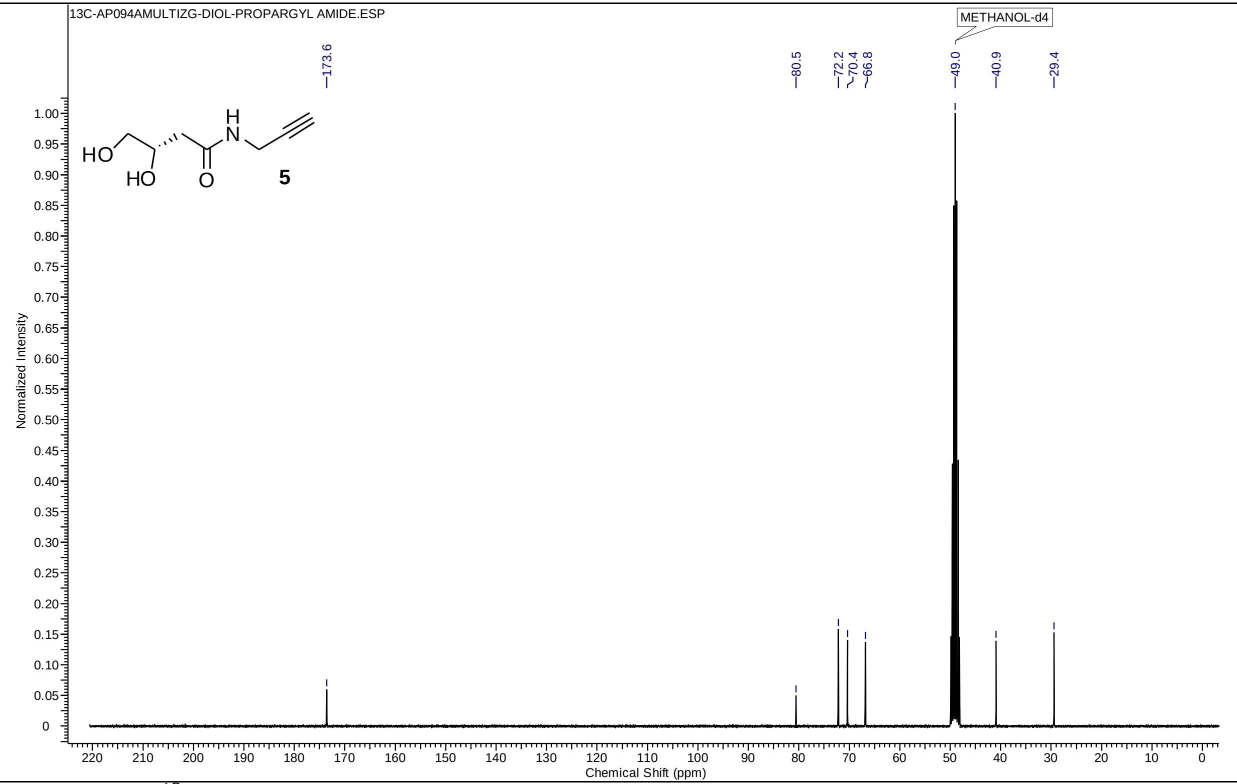

^{13}C-NMR spectrum of (*S*)-3,4-dihydroxy-*N*-(prop-2-ynyl)butanamide **5**

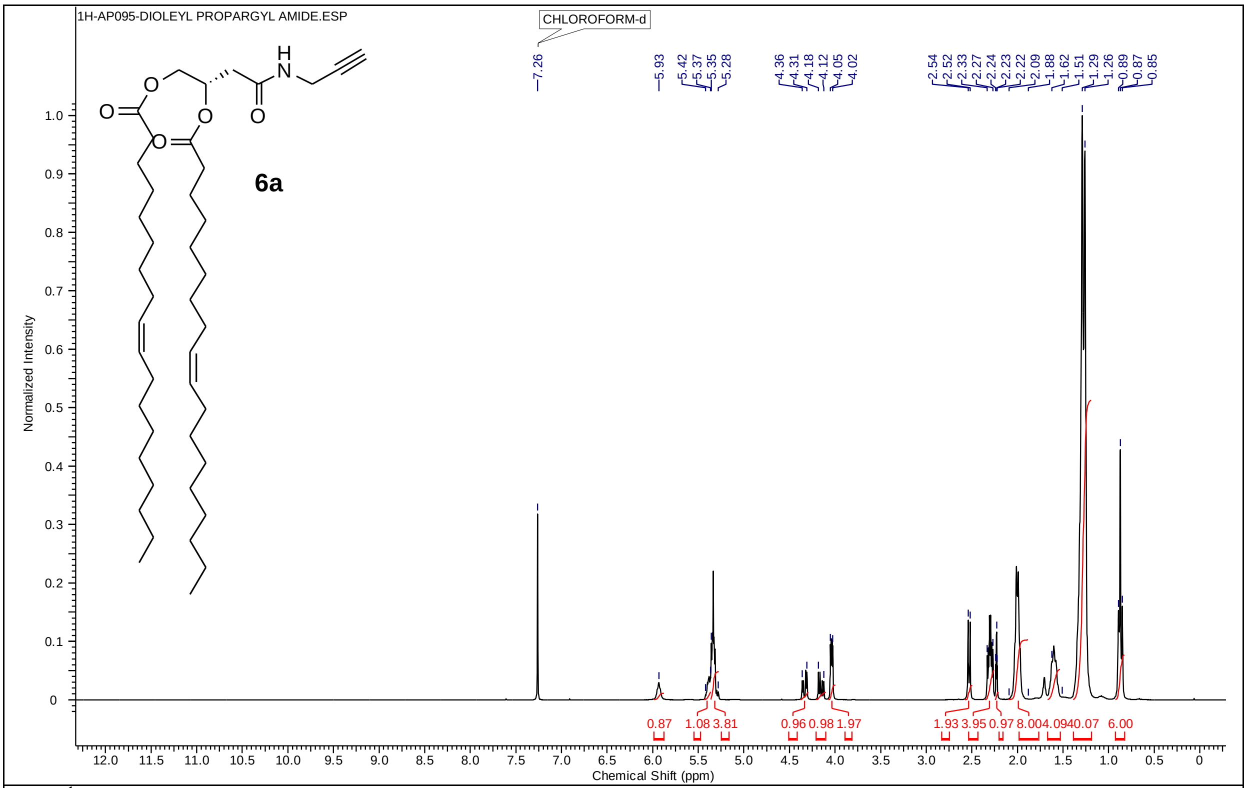

^{1}H-NMR spectrum of (*Z*)-((*S*)-4-oxo-4-(prop-2-ynylamino)butane-1,2-diyl) dioleate **6a**

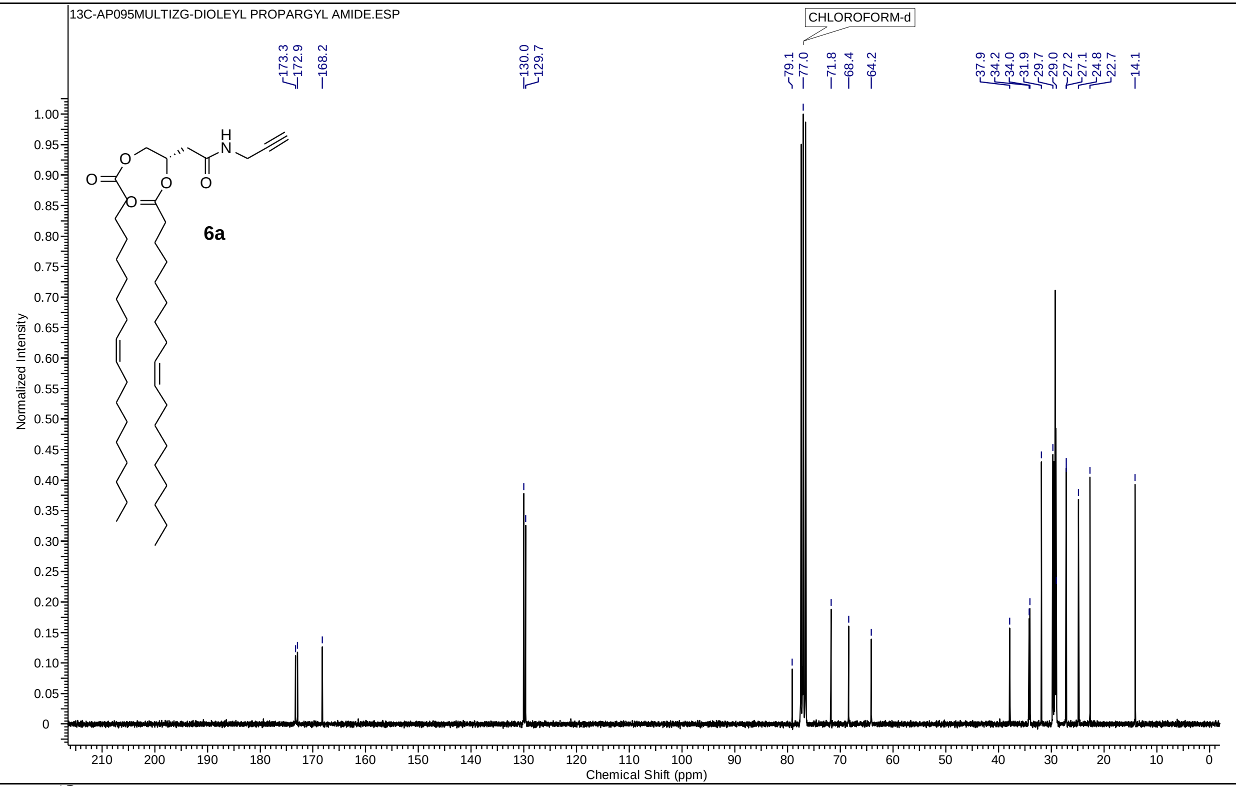

^{13}C-NMR spectrum of (*Z*)-((*S*)-4-oxo-4-(prop-2-ynylamino)butane-1,2-diyl) dioleate **6a**

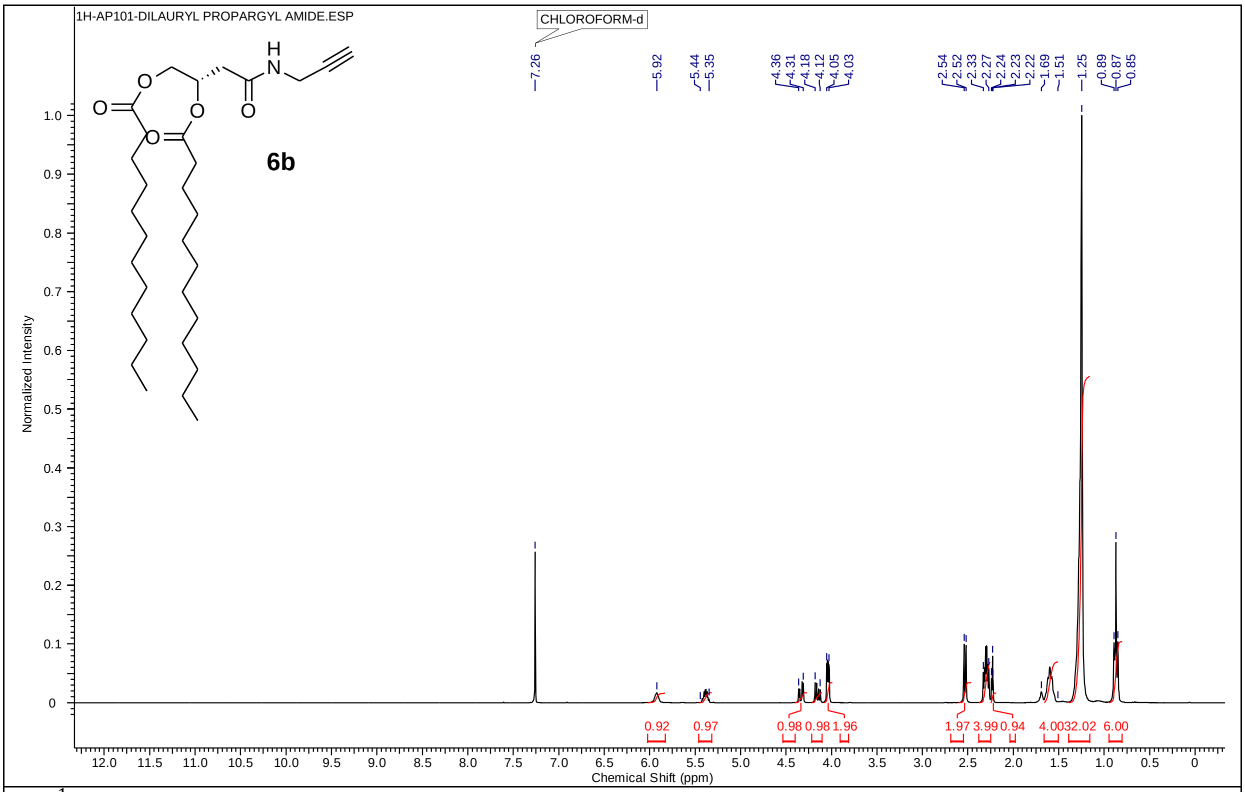

^{1}H-NMR spectrum of (*S*)-4-oxo-4-(prop-2-ynylamino)butane-1,2-diyl didodecanoate **6b**

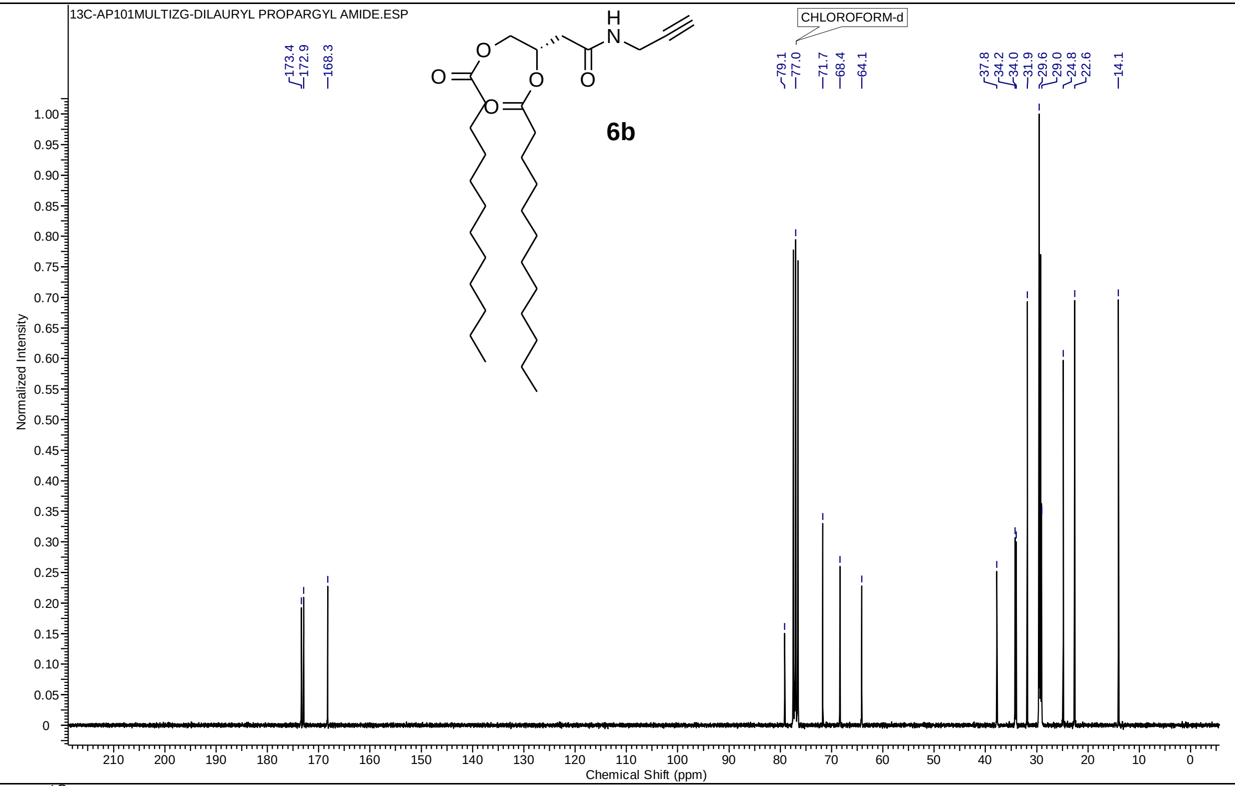

^{13}C-NMR spectrum of (*S*)-4-oxo-4-(prop-2-ynylamino)butane-1,2-diyl didodecanoate **6b**

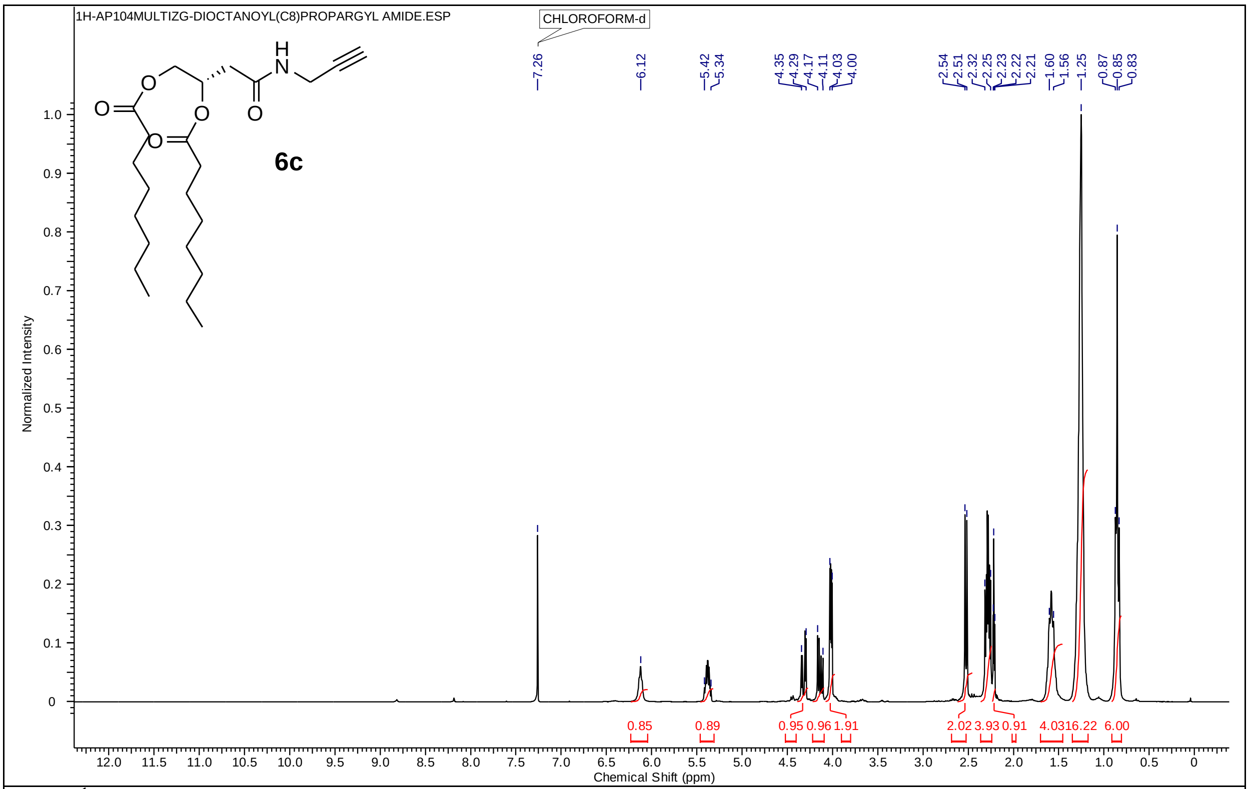

^{1}H-NMR spectrum of (*S*)-4-oxo-4-(prop-2-ynylamino)butane-1,2-diyl dioctanoate **6c**

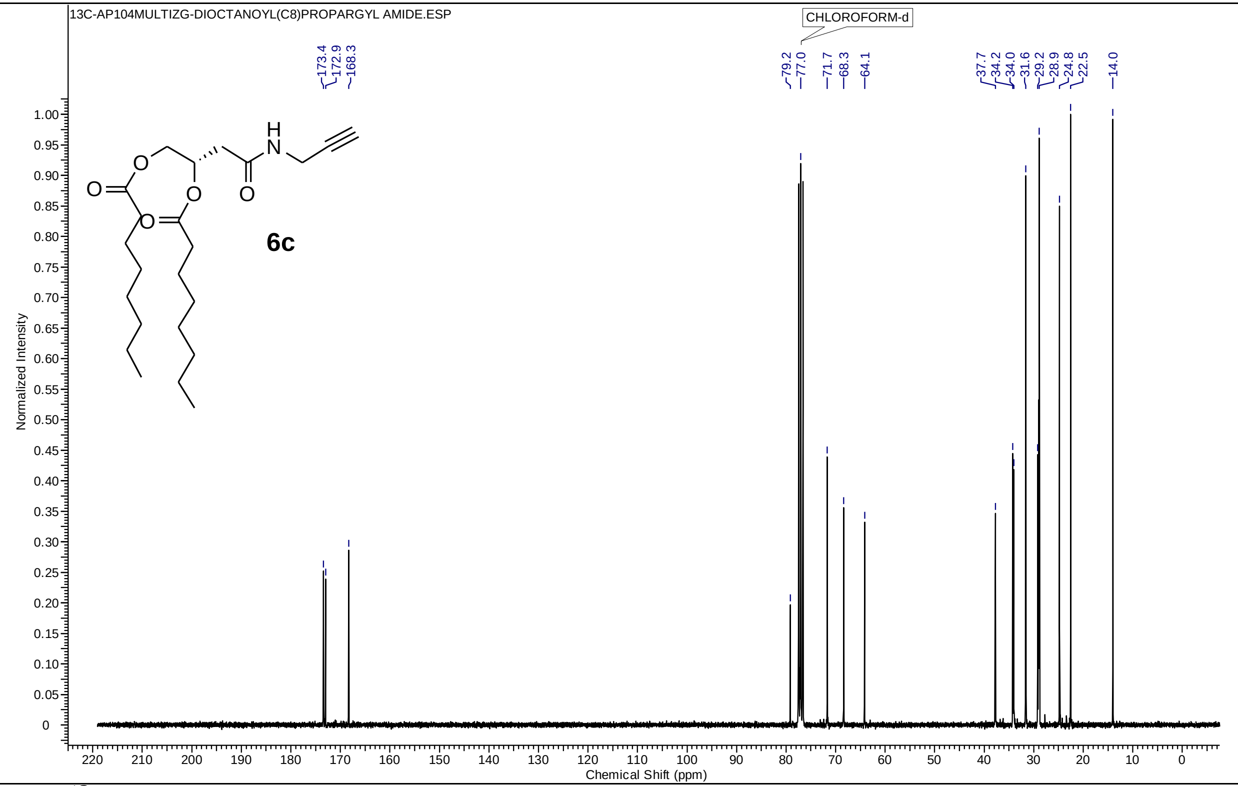

^{13}C-NMR spectrum of (*S*)-4-oxo-4-(prop-2-ynylamino)butane-1,2-diyl dioctanoate **6c**

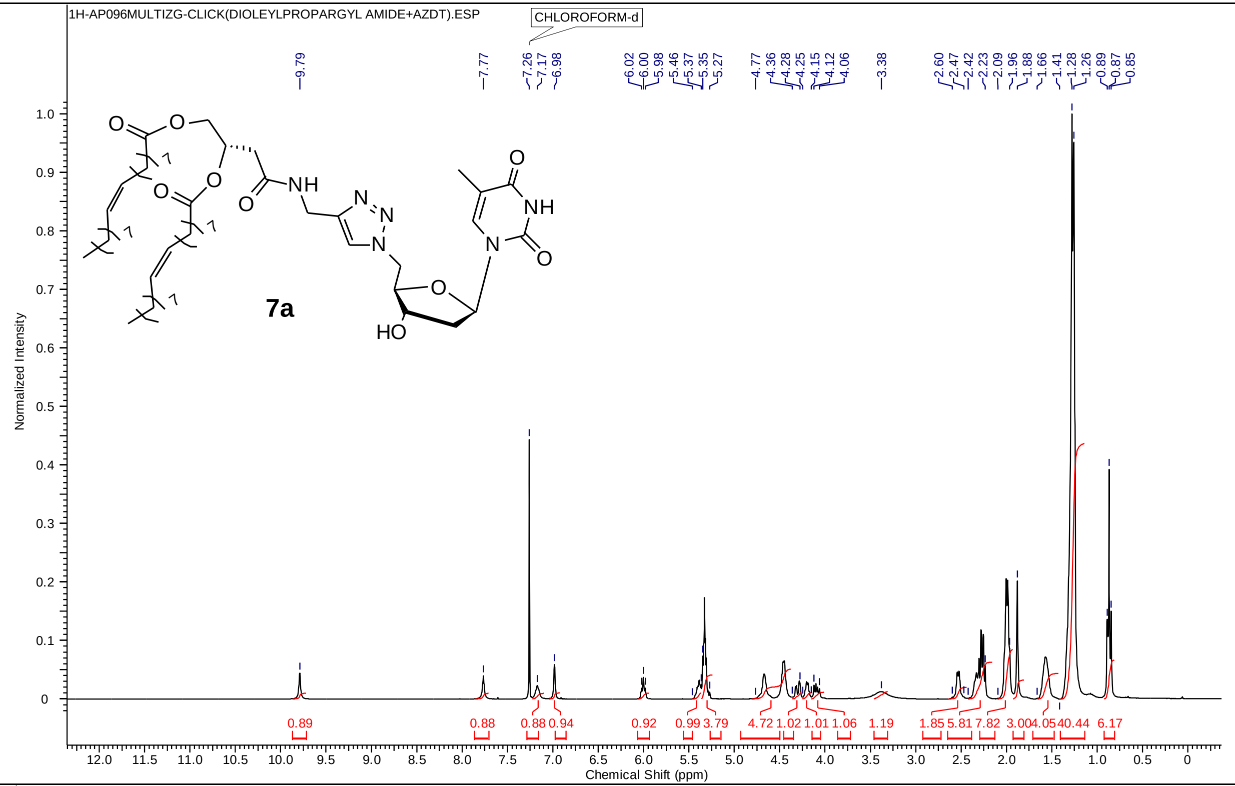

^{1}H-NMR spectrum of (*Z*)-((*S*)-4-(1-(((2*R*,3*S*,5*R*)-3-hydroxy-5-(5-methyl-2,4-dioxo-3,4-dihydropyrimidin-1(2*H*)-yl)tetrahydrofuran-2-yl)methyl)-1*H*-1,2,3-triazol-4-ylamino)-4-oxobutane-1,2-diyl) dioleate **7a**

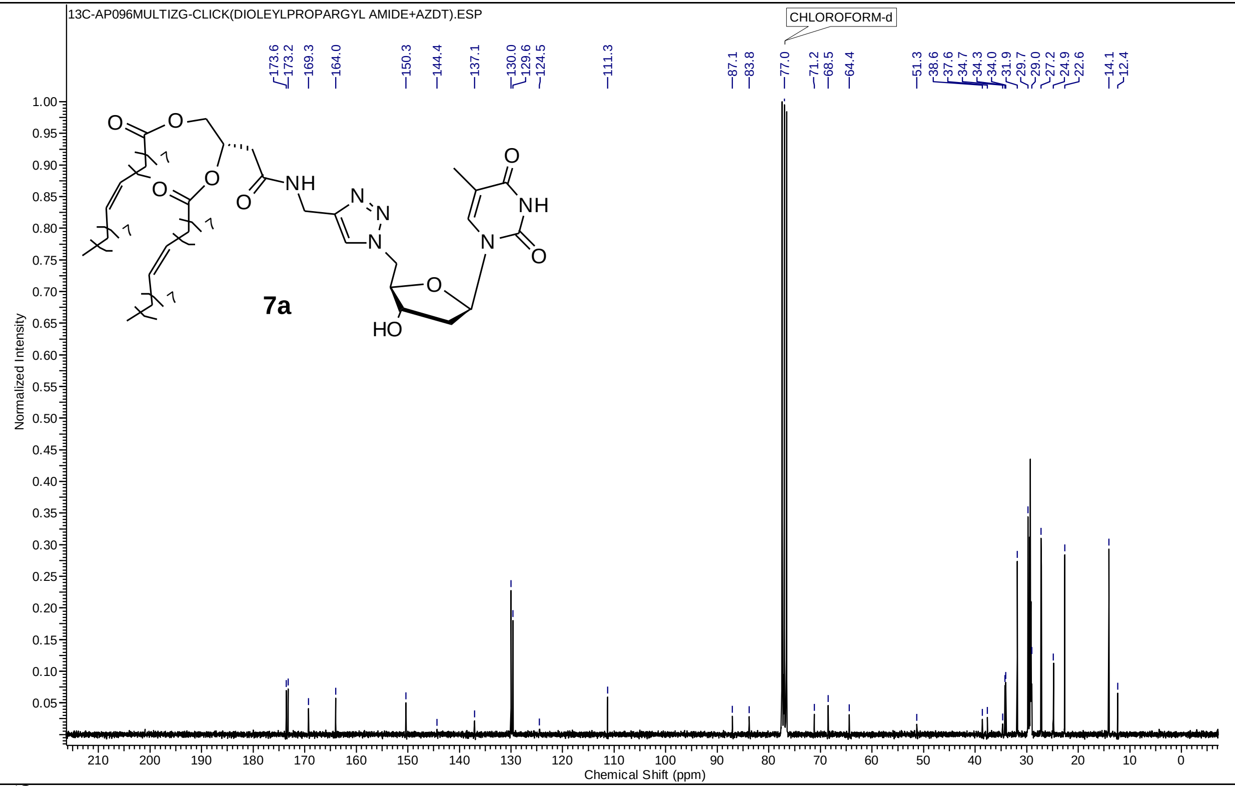

^{13}C-NMR spectrum of (*Z*)-((*S*)-4-(1-(((2*R*,3*S*,5*R*)-3-hydroxy-5-(5-methyl-2,4-dioxo-3,4-dihydropyrimidin-1(2*H*)-yl)tetrahydrofuran-2-yl)methyl)-1*H*-1,2,3-triazol-4-ylamino)-4-oxobutane-1,2-diyl) dioleate **7a**

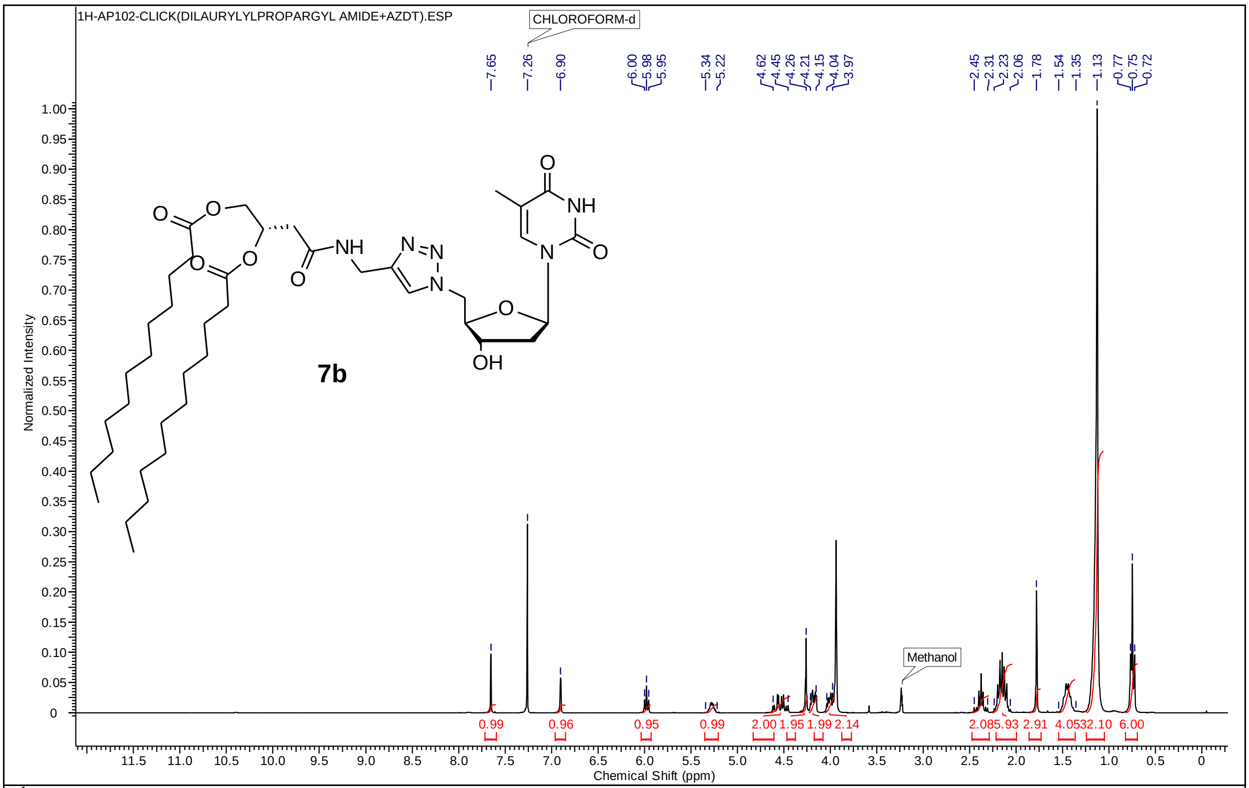

^{1}H-NMR spectrum of (*S*)-4-(1-(((2*R*,3*S*,5*R*)-3-hydroxy-5-(5-methyl-2,4-dioxo-3,4-dihydropyrimidin-1(2*H*)-yl)tetrahydrofuran-2-yl)methyl)-1*H*-1,2,3-triazol-4-ylamino)-4-oxobutane-1,2-diyl didodecanoate **7b**

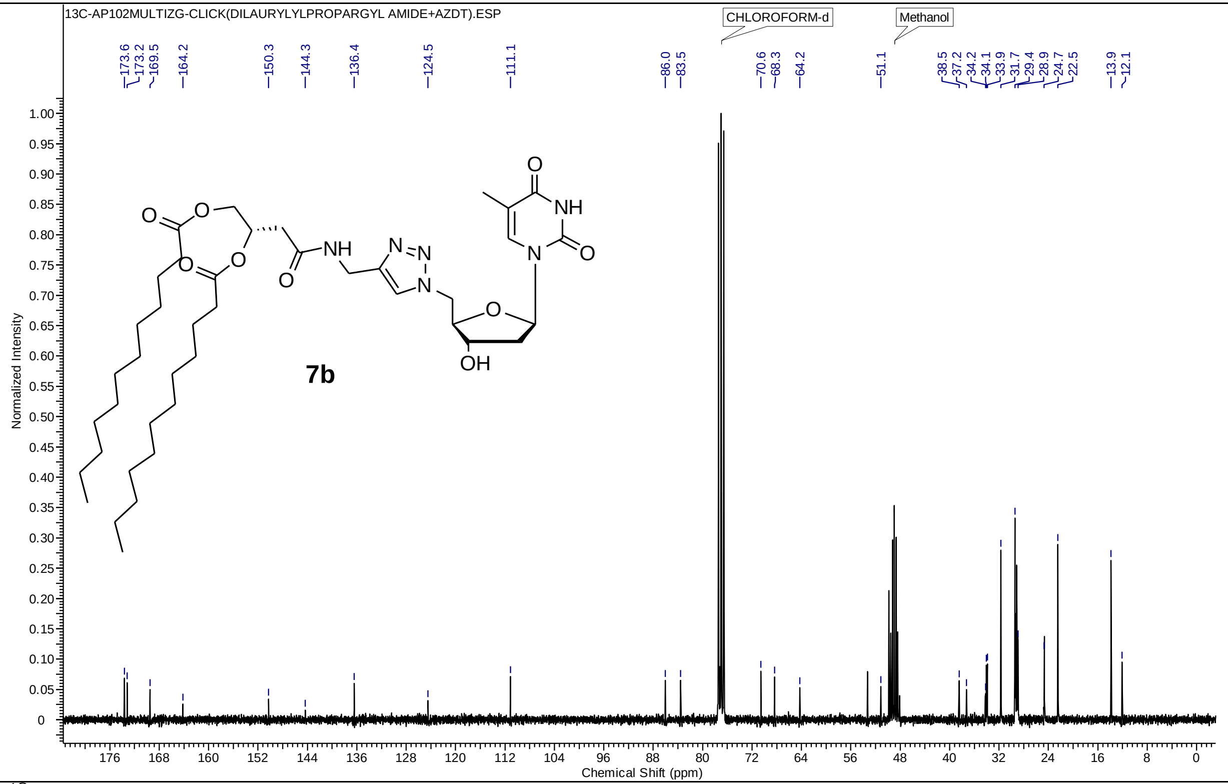

^{13}C-NMR spectrum of (*S*)-4-(1-(((2*R*,3*S*,5*R*)-3-hydroxy-5-(5-methyl-2,4-dioxo-3,4-dihydropyrimidin-1(2*H*)-yl)tetrahydrofuran-2-yl)methyl)-1*H*-1,2,3-triazol-4-ylamino)-4-oxobutane-1,2-diyl didodecanoate **7b**

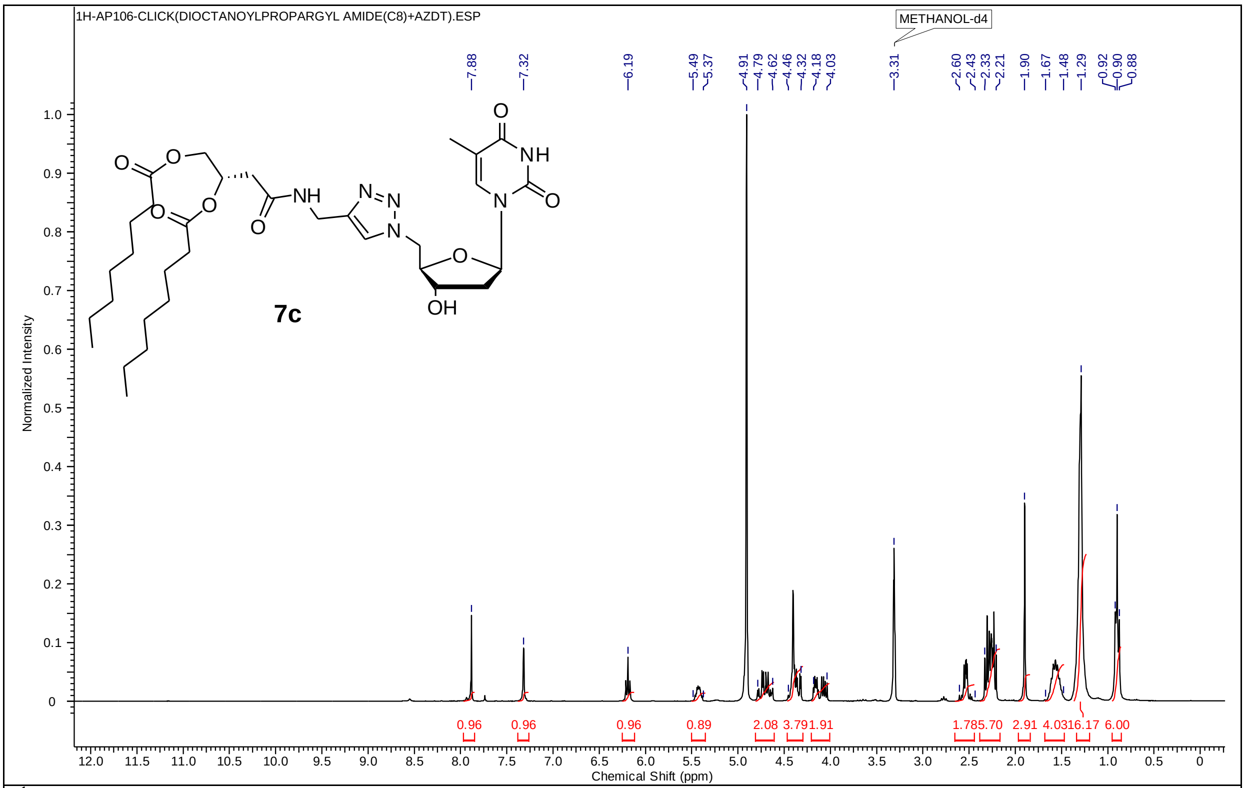

^{1}H-NMR spectrum of (*S*)-4-(1-(((2*R*,3*S*,5*R*)-3-hydroxy-5-(5-methyl-2,4-dioxo-3,4-dihydropyrimidin-1(2*H*)-yl)tetrahydrofuran-2-yl)methyl)-1*H*-1,2,3-triazol-4-ylamino)-4-oxobutane-1,2-diyl dioctanoate **7c**

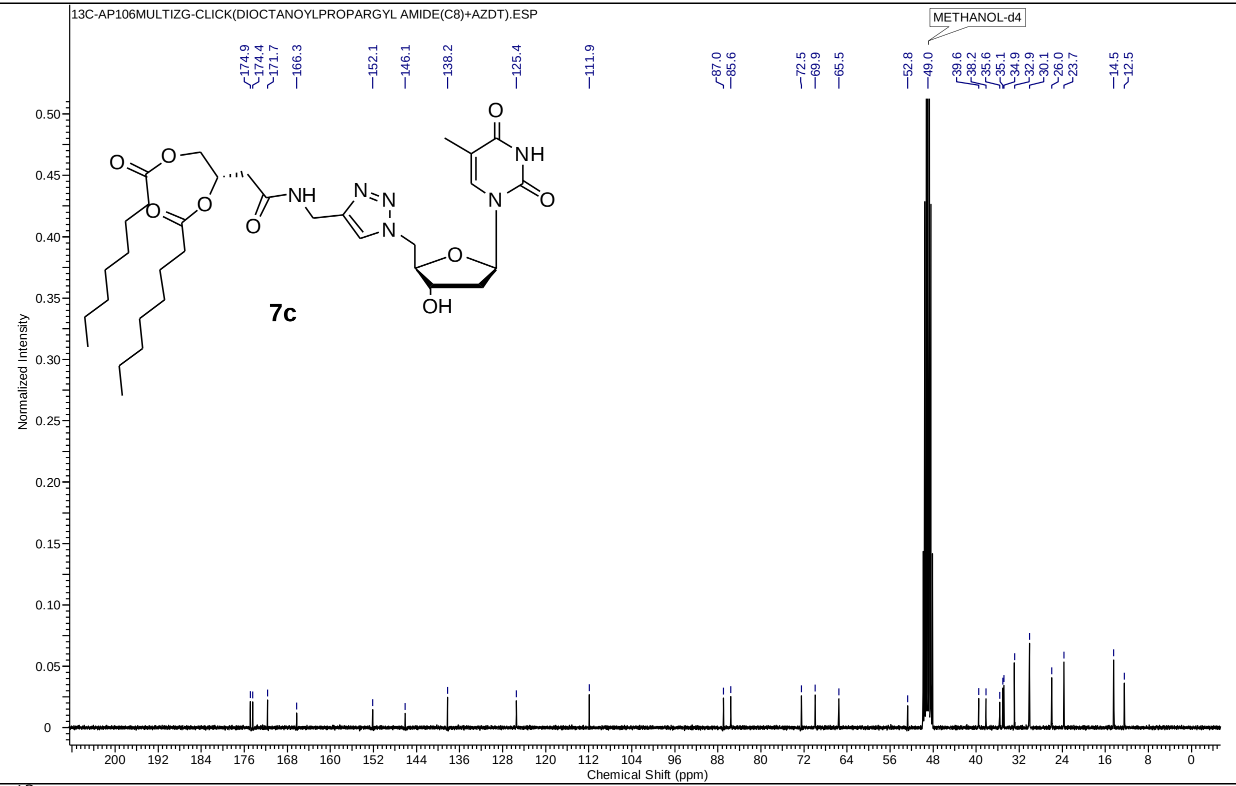

^{13}C-NMR spectrum of (*S*)-4-(1-(((2*R*,3*S*,5*R*)-3-hydroxy-5-(5-methyl-2,4-dioxo-3,4-dihydropyrimidin-1(2*H*)-yl)tetrahydrofuran-2-yl)methyl)-1*H*-1,2,3-triazol-4-ylamino)-4-oxobutane-1,2-diyl dioctanoate **7c**

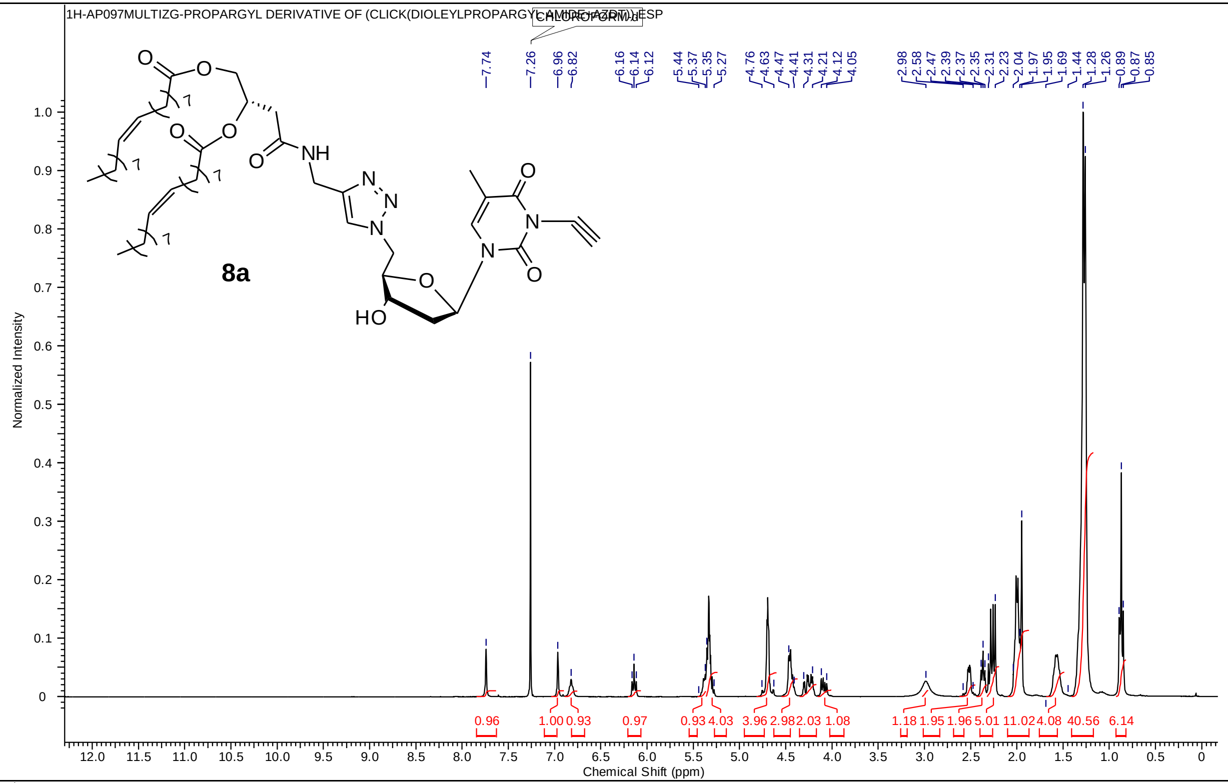

^{1}H-NMR spectrum of (*Z*)-((*S*)-4-(1-(((2*R*,3*S*,5*R*)-3-hydroxy-5-(5-methyl-2,4-dioxo-3-(prop-2-ynyl)-3,4-dihydropyrimidin-1(2*H*)-yl)tetrahydrofuran-2-yl)methyl)-1*H*-1,2,3-triazol-4-ylamino)-4-oxobutane-1,2-diyl) dioleate **8a**

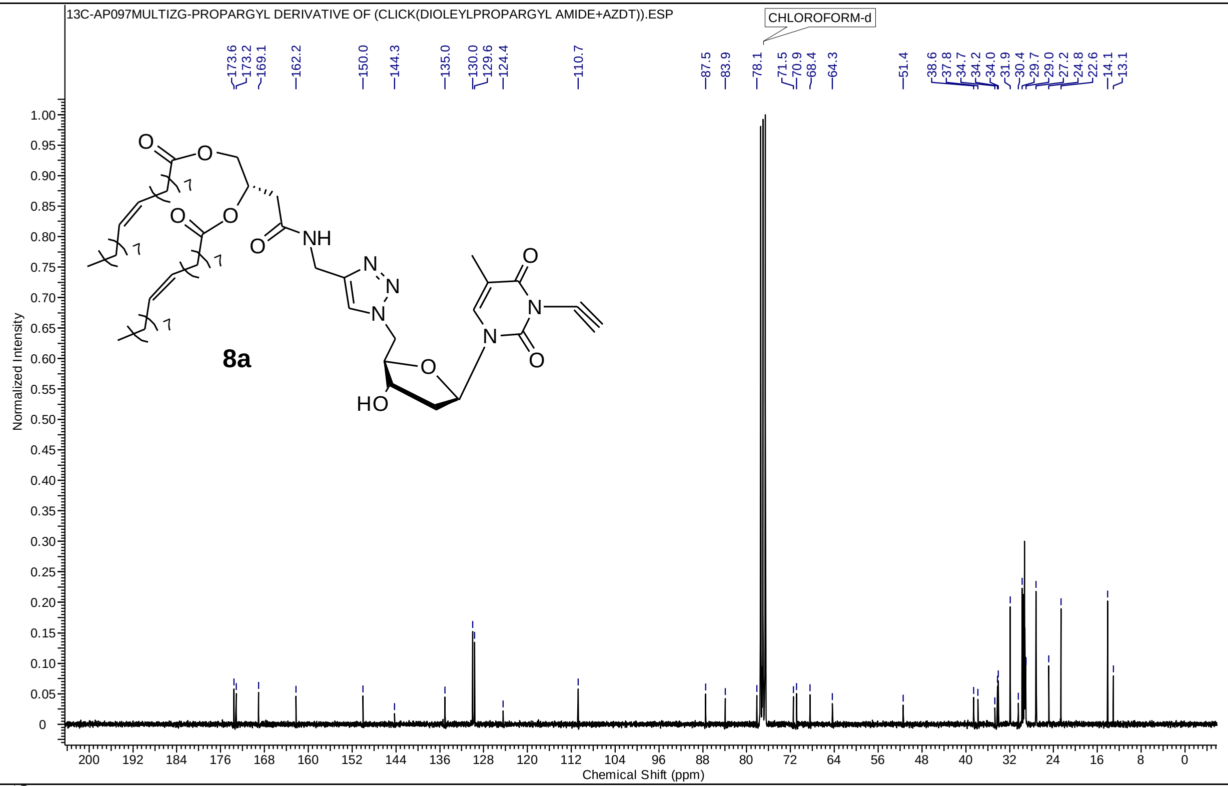

^{13}C-NMR spectrum of (*Z*)-((*S*)-4-(1-(((2*R*,3*S*,5*R*)-3-hydroxy-5-(5-methyl-2,4-dioxo-3-(prop-2-ynyl)-3,4-dihydropyrimidin-1(2*H*)-yl)tetrahydrofuran-2-yl)methyl)-1*H*-1,2,3-triazol-4-ylamino)-4-oxobutane-1,2-diyl) dioleate **8a**

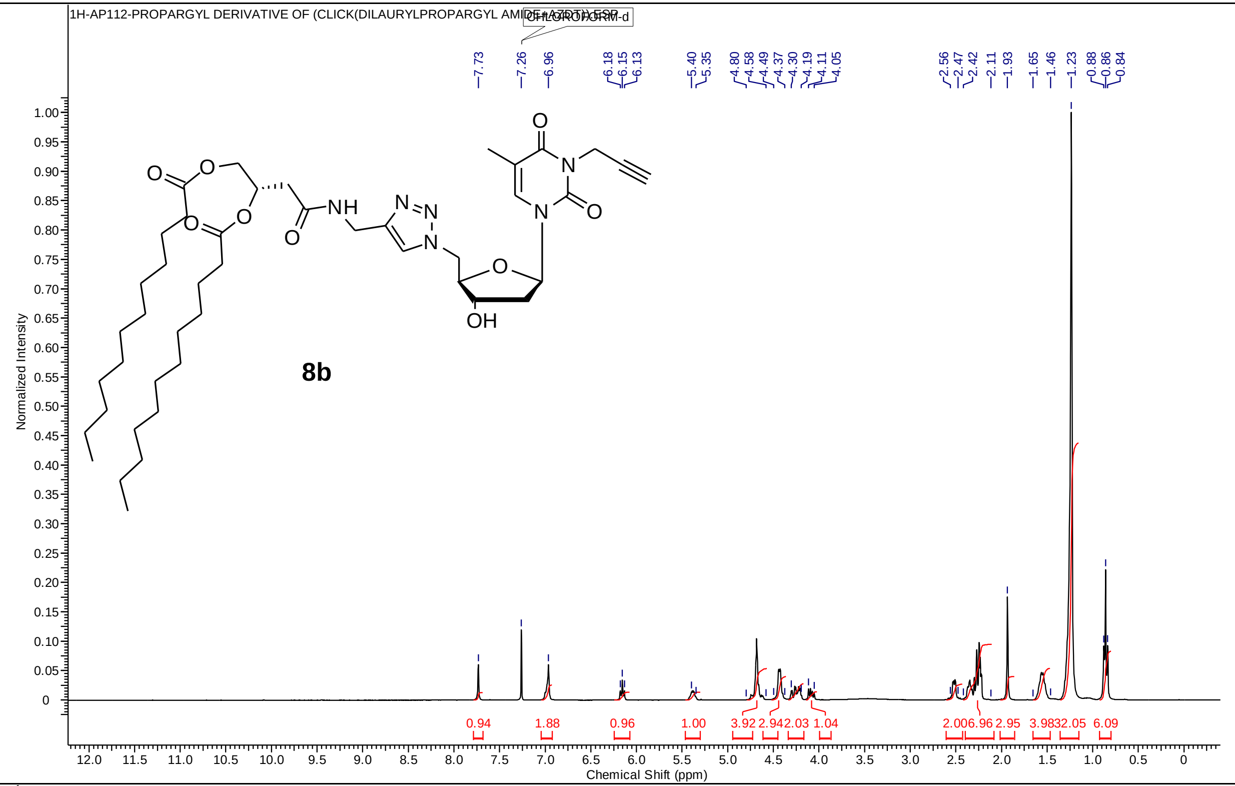

^{1}H-NMR spectrum of (*S*)-4-(1-(((2*R*,3*S*,5*R*)-3-hydroxy-5-(5-methyl-2,4-dioxo-3-(prop-2-ynyl)-3,4-dihydropyrimidin-1(2*H*)-yl)tetrahydrofuran-2-yl)methyl)-1*H*-1,2,3-triazol-4-ylamino)-4-oxobutane-1,2-diyl didodecanoate **8b**

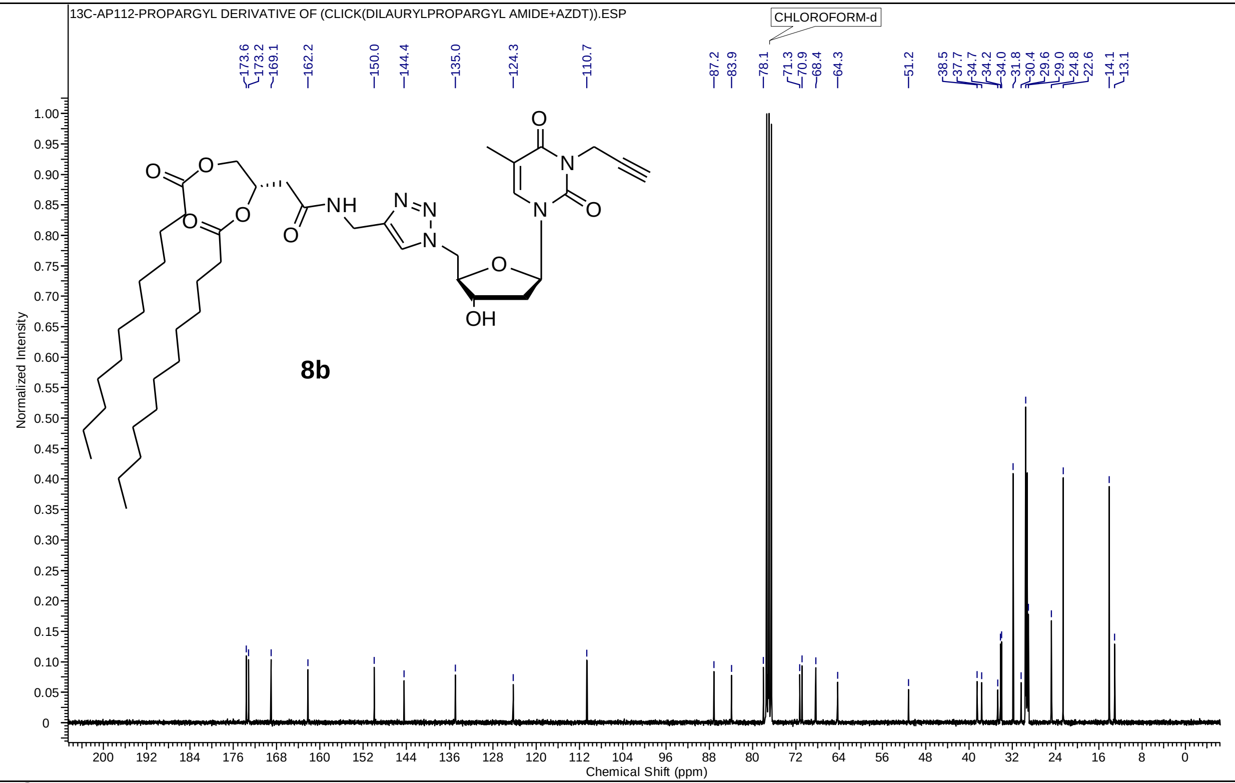

^{13}C-NMR spectrum of (*S*)-4-(1-(((2*R*,3*S*,5*R*)-3-hydroxy-5-(5-methyl-2,4-dioxo-3-(prop-2-ynyl)-3,4-dihydropyrimidin-1(2*H*)-yl)tetrahydrofuran-2-yl)methyl)-1*H*-1,2,3-triazol-4-ylamino)-4-oxobutane-1,2-diyl didodecanoate **8b**

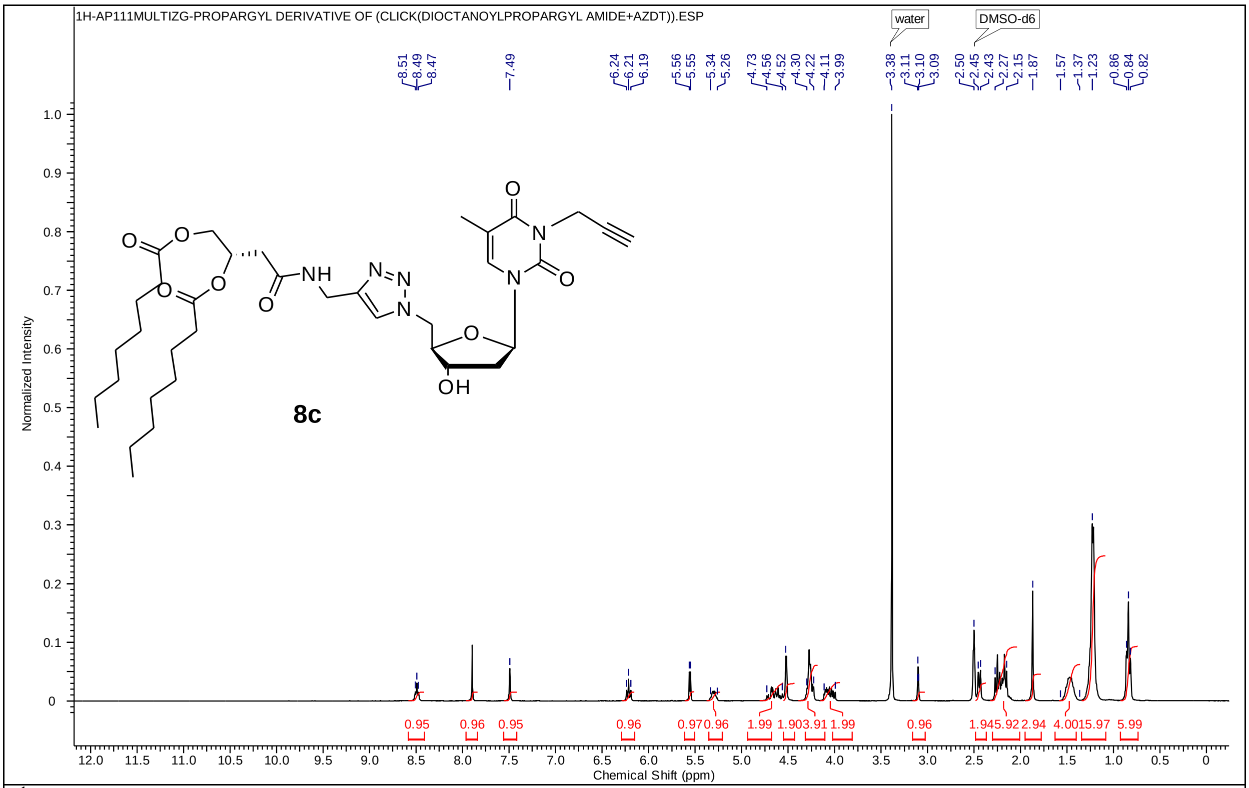

^{1}H-NMR spectrum of (*S*)-4-(1-(((2*R*,3*S*,5*R*)-3-hydroxy-5-(5-methyl-2,4-dioxo-3-(prop-2-ynyl)-3,4-dihydropyrimidin-1(2*H*)-yl)tetrahydrofuran-2-yl)methyl)-1*H*-1,2,3-triazol-4-ylamino)-4-oxobutane-1,2-diyl dioctanoate **8c**

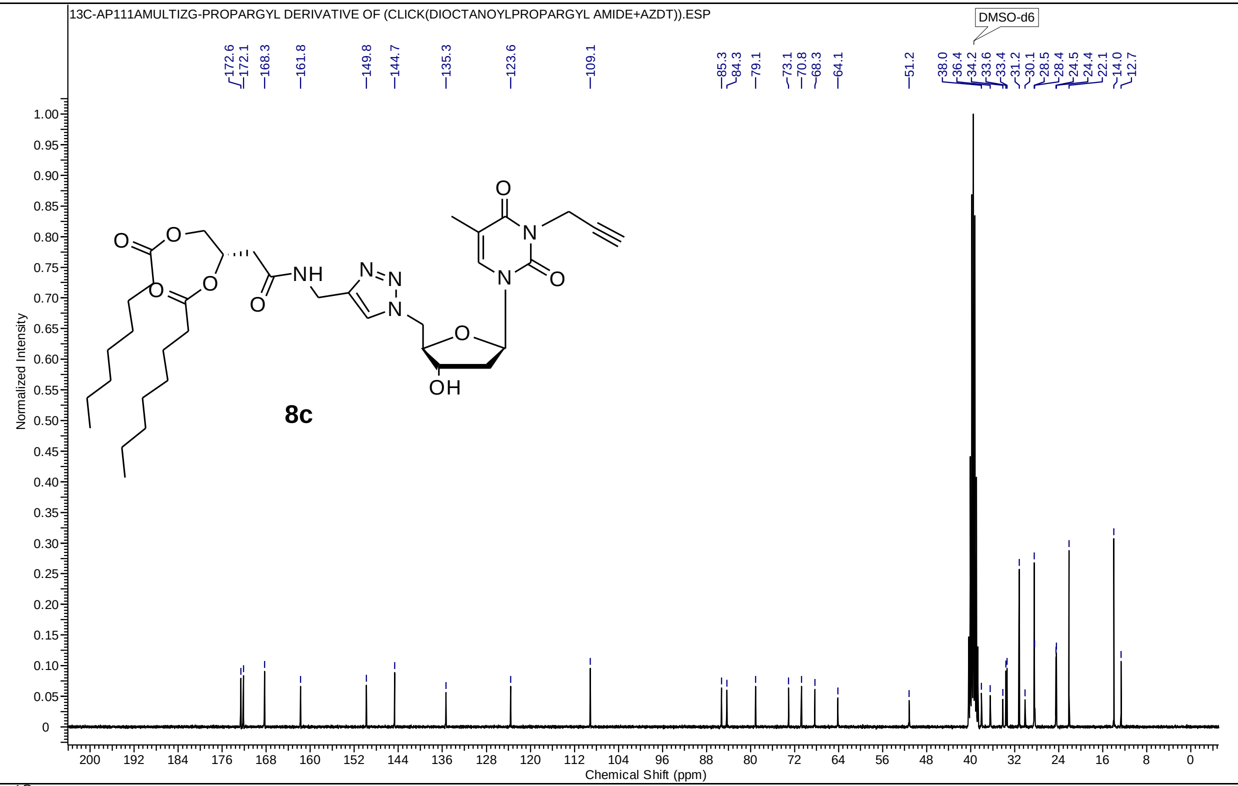

^{13}C-NMR spectrum of (*S*)-4-(1-(((2*R*,3*S*,5*R*)-3-hydroxy-5-(5-methyl-2,4-dioxo-3-(prop-2-ynyl)-3,4-dihydropyrimidin-1(2*H*)-yl)tetrahydrofuran-2-yl)methyl)-1*H*-1,2,3-triazol-4-ylamino)-4-oxobutane-1,2-diyl dioctanoate **8c**

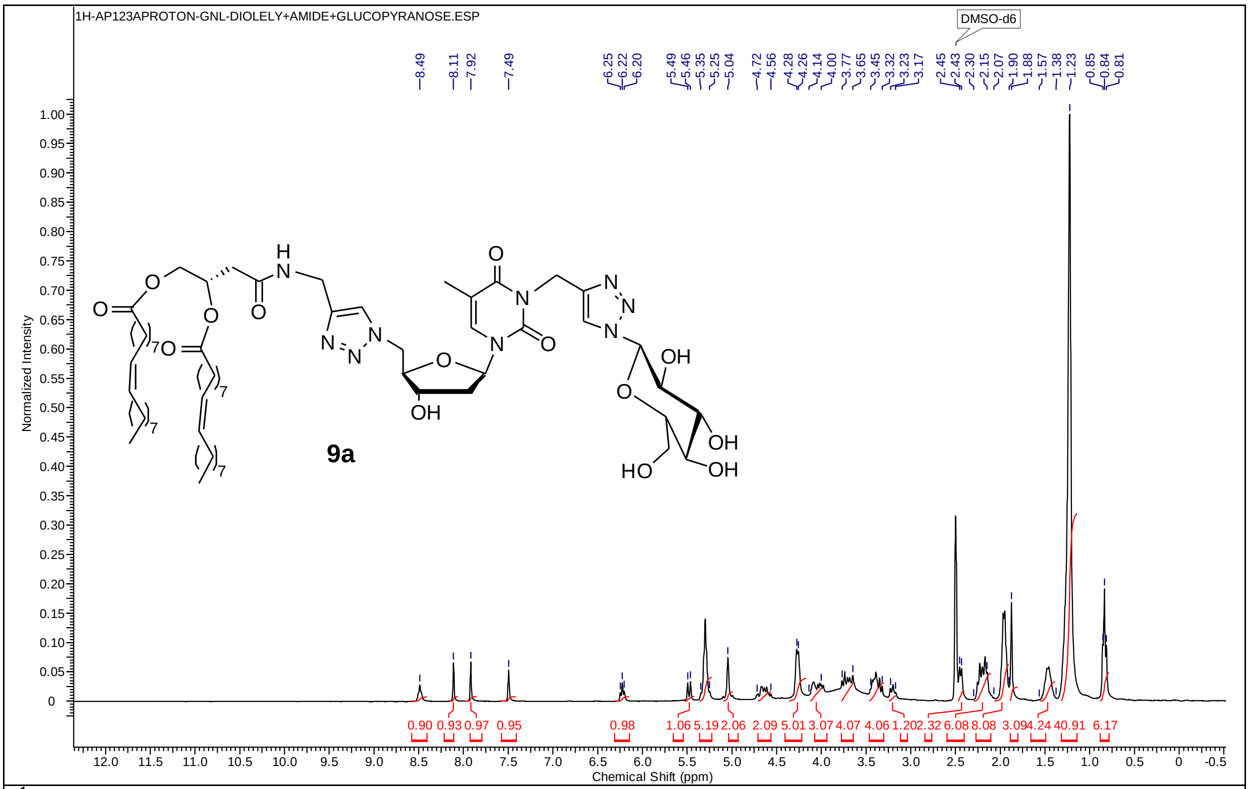

^{1}H-NMR spectrum of (*Z*)-4-(1-(((2*R*,3*S*,5*R*)-3-hydroxy-5-(5-methyl-2,4-dioxo-3-((1-((2*R*,3*R*,4*S*,5*S*,6*R*)-3,4,5-trihydroxy-6-(hydroxymethyl)tetrahydro-2*H*-pyran-2-yl)-1*H*-1,2,3-triazol-4-yl)methyl)-3,4-dihydropyrimidin-1(2*H*)-yl)tetrahydrofuran-2-yl)methyl)-1*H*-1,2,3-triazol-4-ylamino)-4-oxobutane-1,2-diyl dioleate **9a**

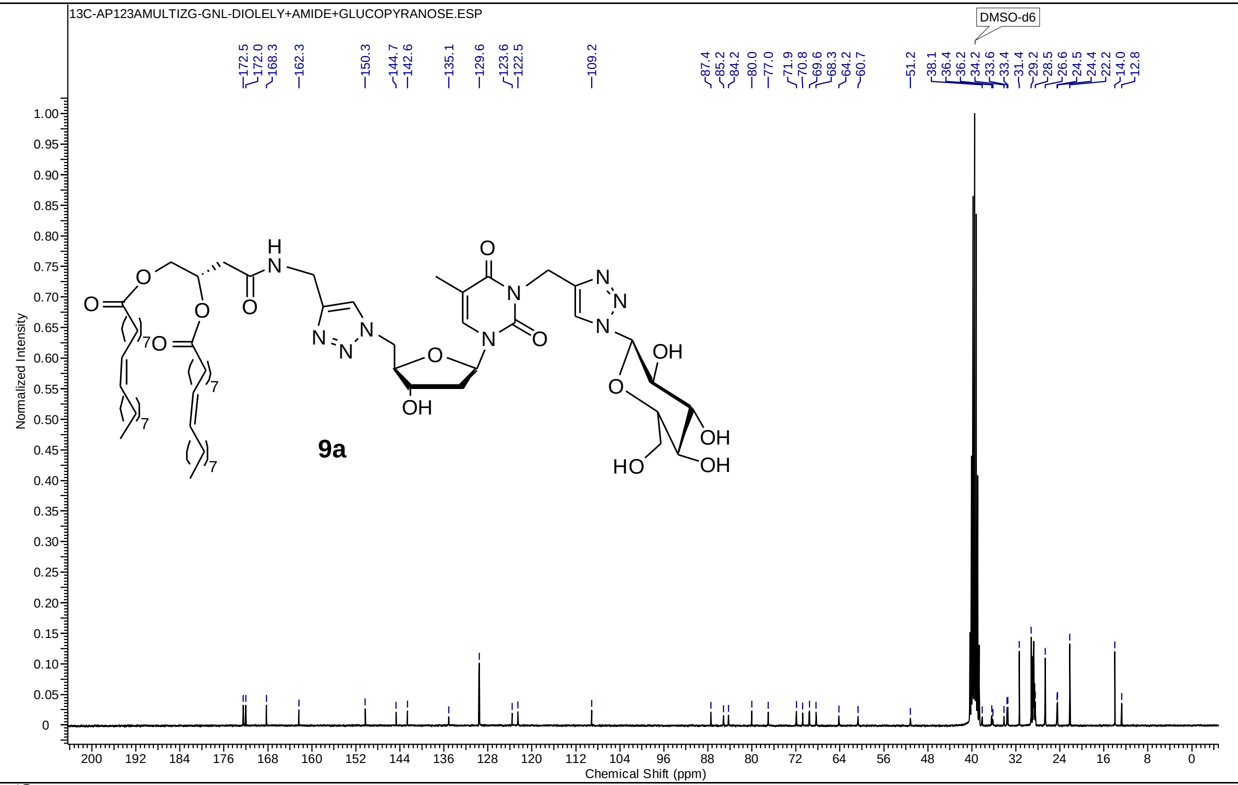

^{13}C-NMR spectrum of (*Z*)-4-(1-(((2*R*,3*S*,5*R*)-3-hydroxy-5-(5-methyl-2,4-dioxo-3-((1-((2*R*,3*R*,4*S*,5*S*,6*R*)-3,4,5-trihydroxy-6-(hydroxymethyl)tetrahydro-2*H*-pyran-2-yl)-1*H*-1,2,3-triazol-4-yl)methyl)-3,4-dihydropyrimidin-1(2*H*)-yl)tetrahydrofuran-2-yl)methyl)-1*H*-1,2,3-triazol-4-ylamino)-4-oxobutane-1,2-diyl dioleate **9a**

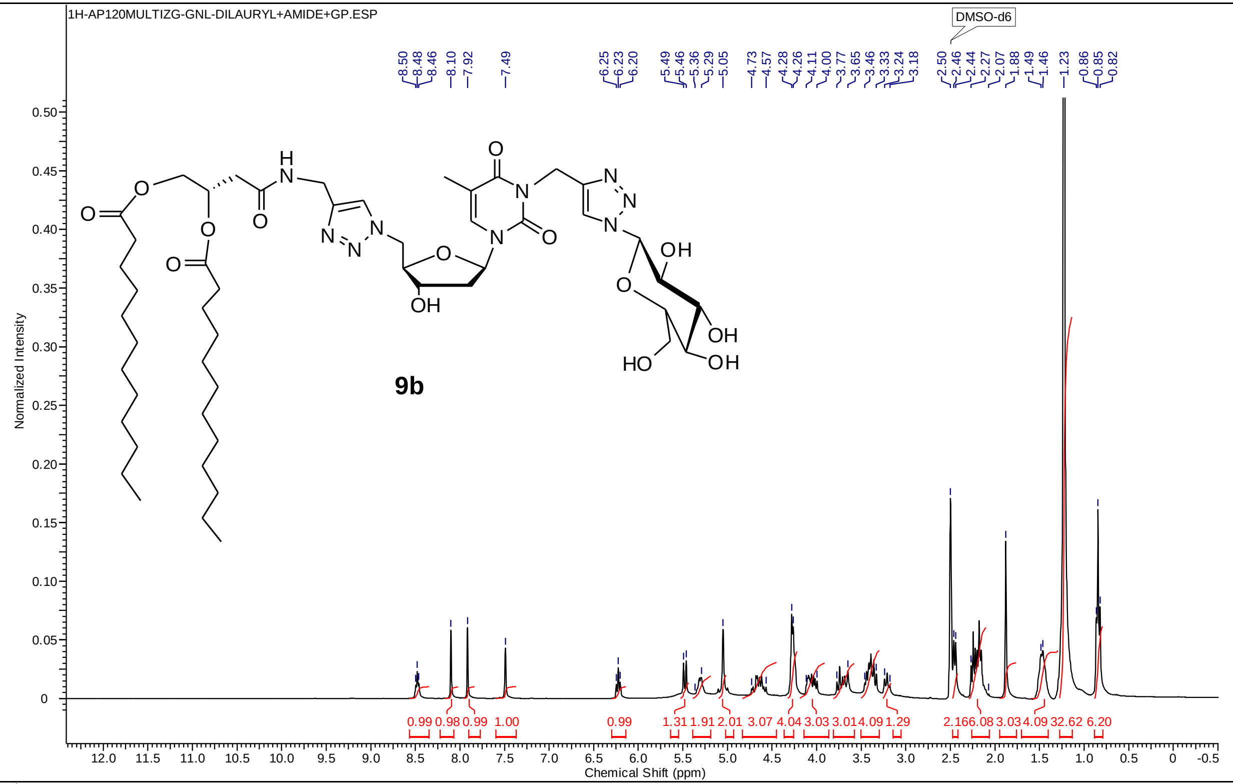

^{1}H-NMR spectrum of 4-(1-(((2*R*,3*S*,5*R*)-3-hydroxy-5-(5-methyl-2,4-dioxo-3-((1-((2*R*,3*R*,4*S*,5*S*,6*R*)-3,4,5-trihydroxy-6-(hydroxymethyl)tetrahydro-2*H*-pyran-2-yl)-1H-1,2,3-triazol-4-yl)methyl)-3,4-dihydropyrimidin-1(2*H*)-yl)tetrahydrofuran-2-yl)methyl)-1*H*-1,2,3-triazol-4-ylamino)-4-oxobutane-1,2-diyl didodecanoate **9b**

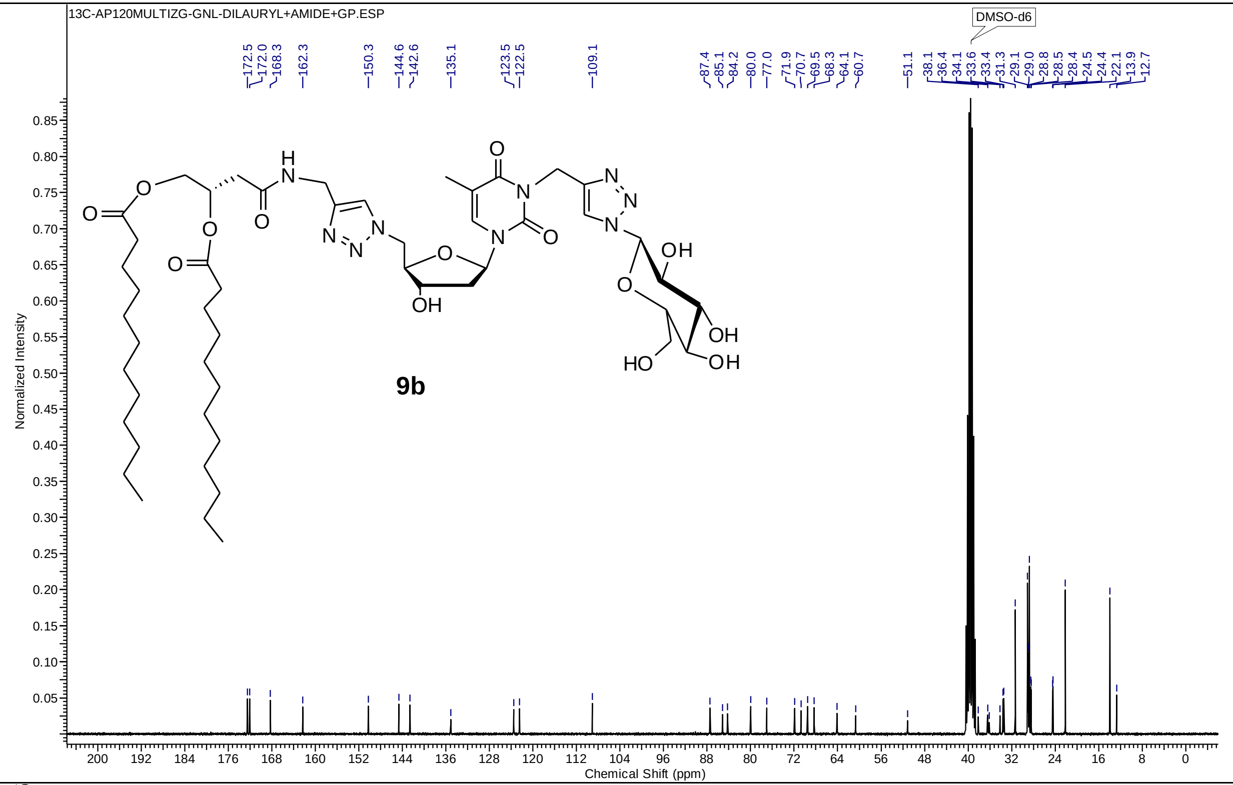

^{13}C-NMR spectrum of 4-(1-(((2*R*,3*S*,5*R*)-3-hydroxy-5-(5-methyl-2,4-dioxo-3-((1-((2*R*,3*R*,4*S*,5*S*,6*R*)-3,4,5-trihydroxy-6-(hydroxymethyl)tetrahydro-2*H*-pyran-2-yl)-1H-1,2,3-triazol-4-yl)methyl)-3,4-dihydropyrimidin-1(2*H*)-yl)tetrahydrofuran-2-yl)methyl)-1*H*-1,2,3-triazol-4-ylamino)-4-oxobutane-1,2-diyl didodecanoate **9b**

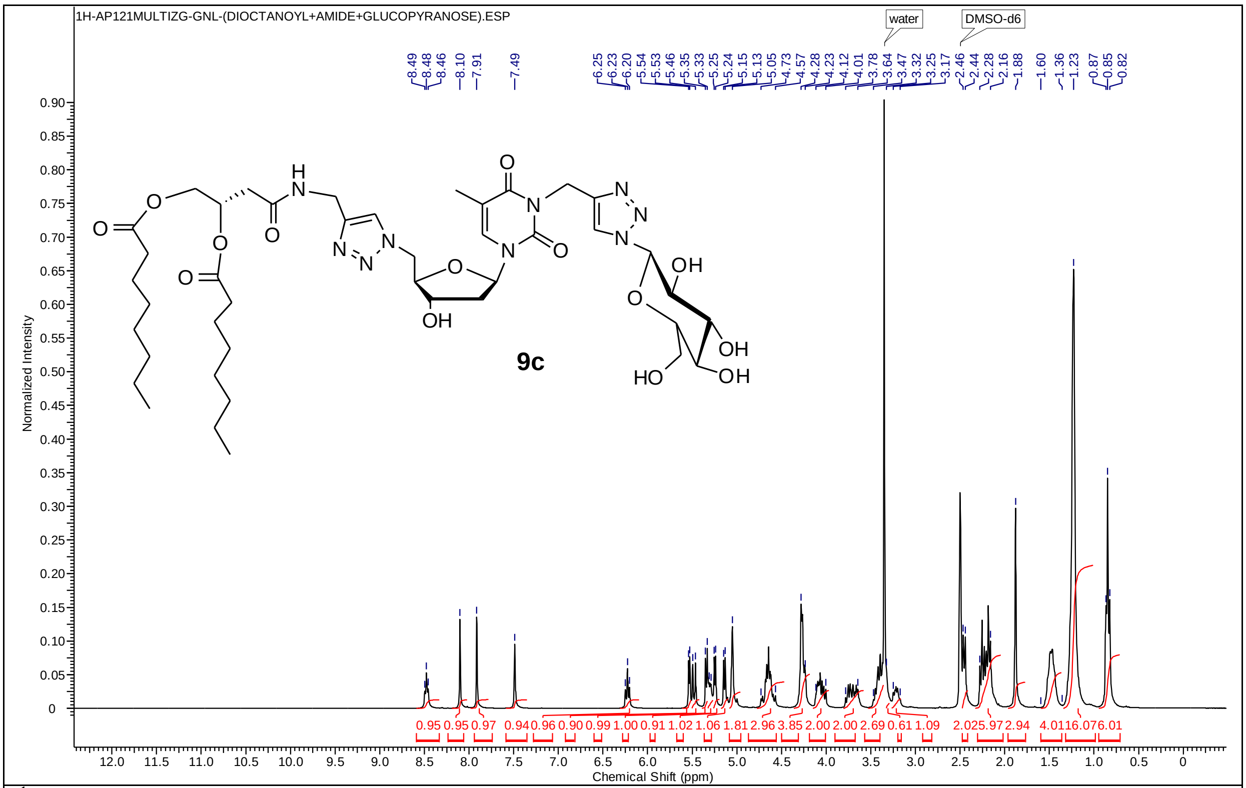

^{1}H-NMR spectrum of 4-(1-(((2*R*,3*S*,5*R*)-3-hydroxy-5-(5-methyl-2,4-dioxo-3-((1-((2*R*,3*R*,4*S*,5*S*,6*R*)-3,4,5-trihydroxy-6-(hydroxymethyl)tetrahydro-2H-pyran-2-yl)-1*H*-1,2,3-triazol-4-yl)methyl)-3,4-dihydropyrimidin-1(2*H*)-yl)tetrahydrofuran-2-yl)methyl)-1*H*-1,2,3-triazol-4-ylamino)-4-oxobutane-1,2-diyl dioctanoate **9c**

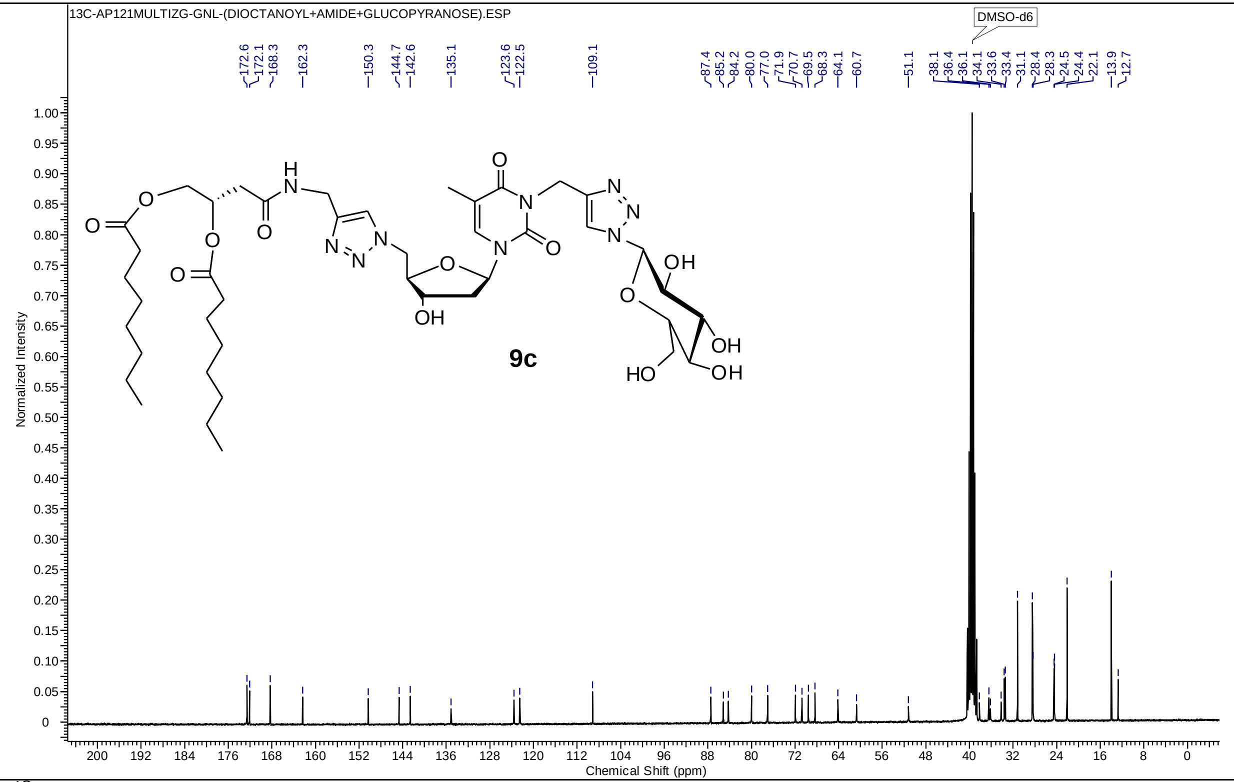

^{13}C-NMR spectrum of 4-(1-(((2*R*,3*S*,5*R*)-3-hydroxy-5-(5-methyl-2,4-dioxo-3-((1-((2*R*,3*R*,4*S*,5*S*,6*R*)-3,4,5-trihydroxy-6-(hydroxymethyl)tetrahydro-2H-pyran-2-yl)-1*H*-1,2,3-triazol-4-yl)methyl)-3,4-dihydropyrimidin-1(2*H*)-yl)tetrahydrofuran-2-yl)methyl)-1*H*-1,2,3-triazol-4-ylamino)-4-oxobutane-1,2-diyl dioctanoate **9c**

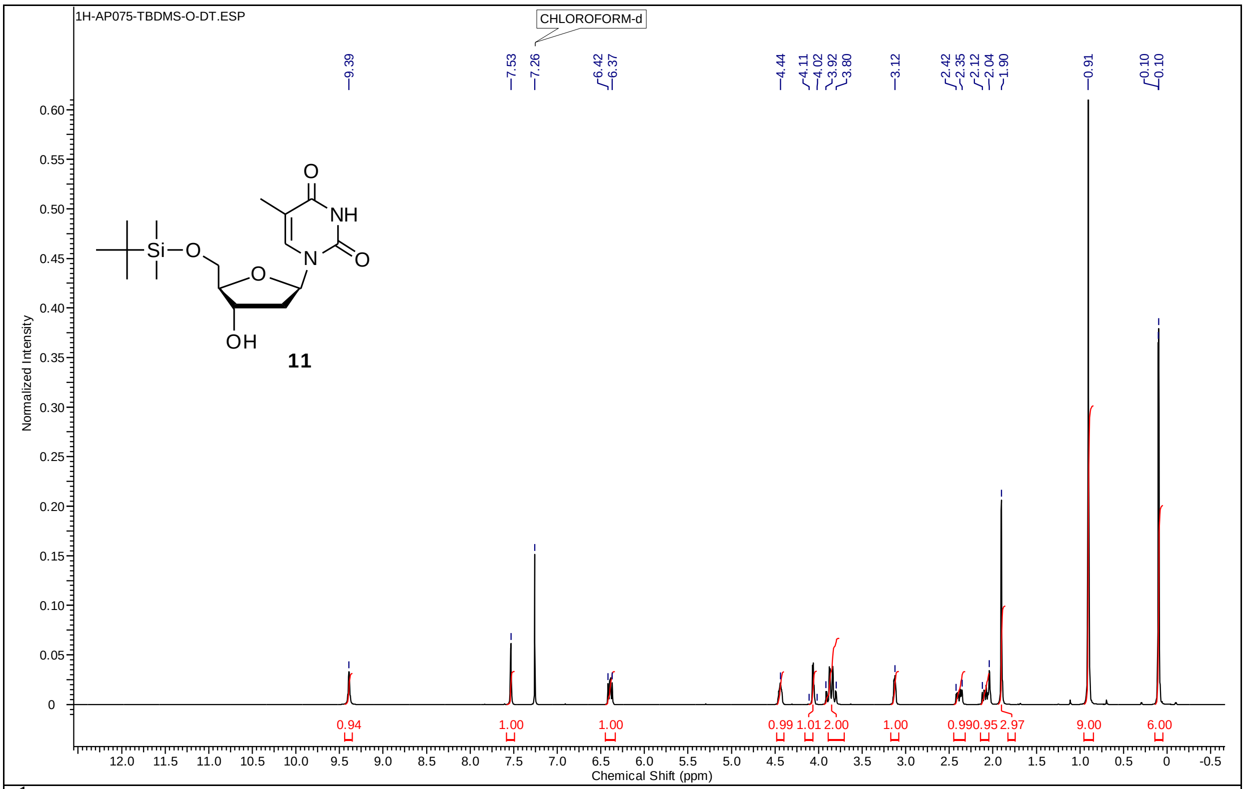

^{1}H-NMR spectrum of 1-((2*R*,4*S*,5*R*)-5-((tert-butyldimethylsilyloxy)methyl)-4-hydroxytetrahydrofuran-2-yl)-5-methylpyrimidine-2,4(1*H*,3*H*)-dione **11**

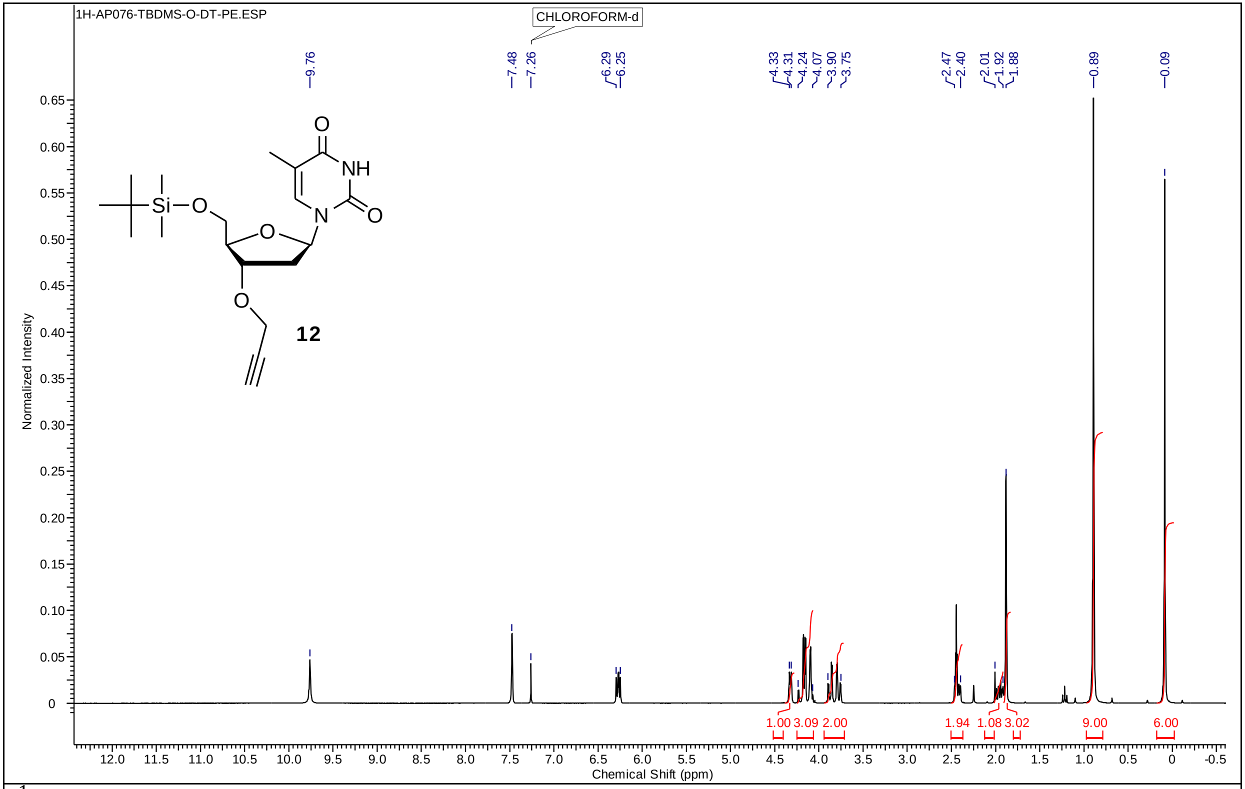

^{1}H-NMR spectrum of 1-((2*R*,4*S*,5*R*)-5-((tert-butyldimethylsilyloxy)methyl)-4-(prop-2-ynyloxy)tetrahydrofuran-2-yl)-5-methylpyrimidine-2,4(1*H*,3*H*)-dione **12**

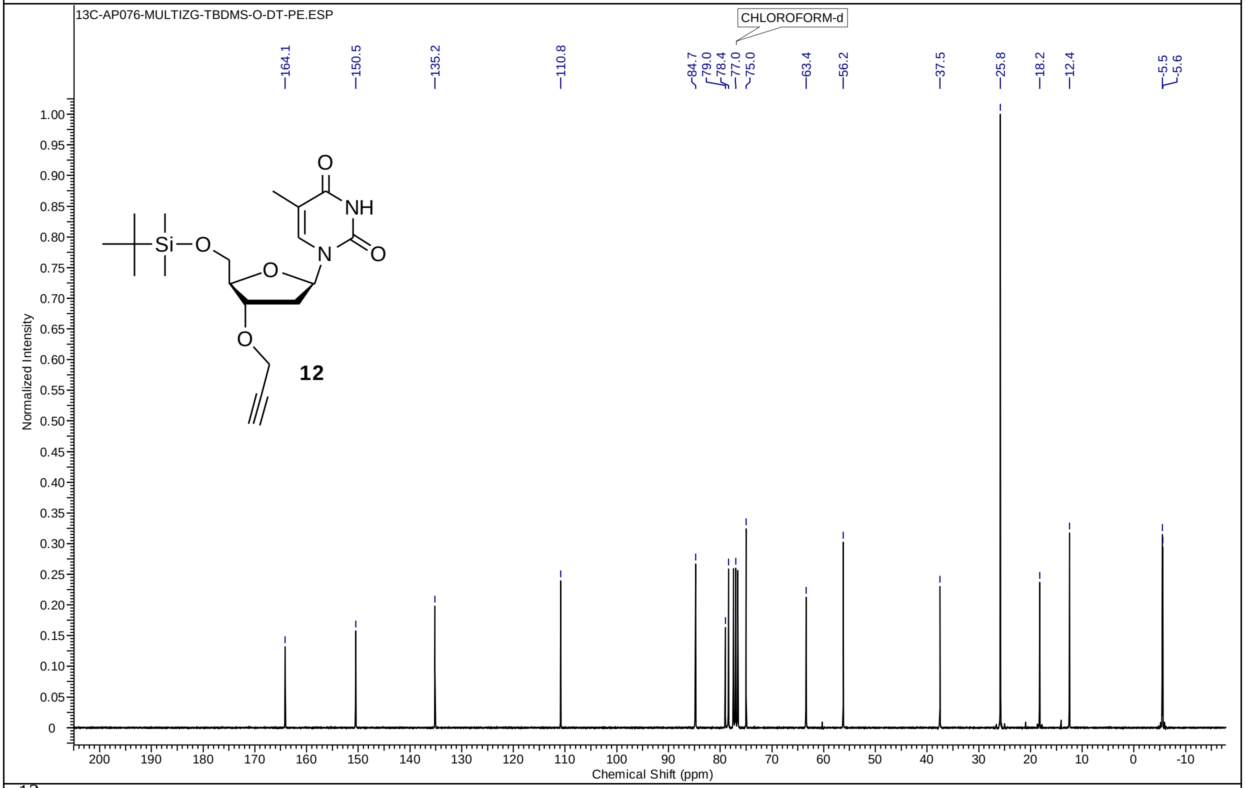

^{13}C-NMR spectrum of 1-((2*R*,4*S*,5*R*)-5-((tert-butyldimethylsilyloxy)methyl)-4-(prop-2-ynyloxy)tetrahydrofuran-2-yl)-5-methylpyrimidine-2,4(1*H*,3*H*)-dione **12**

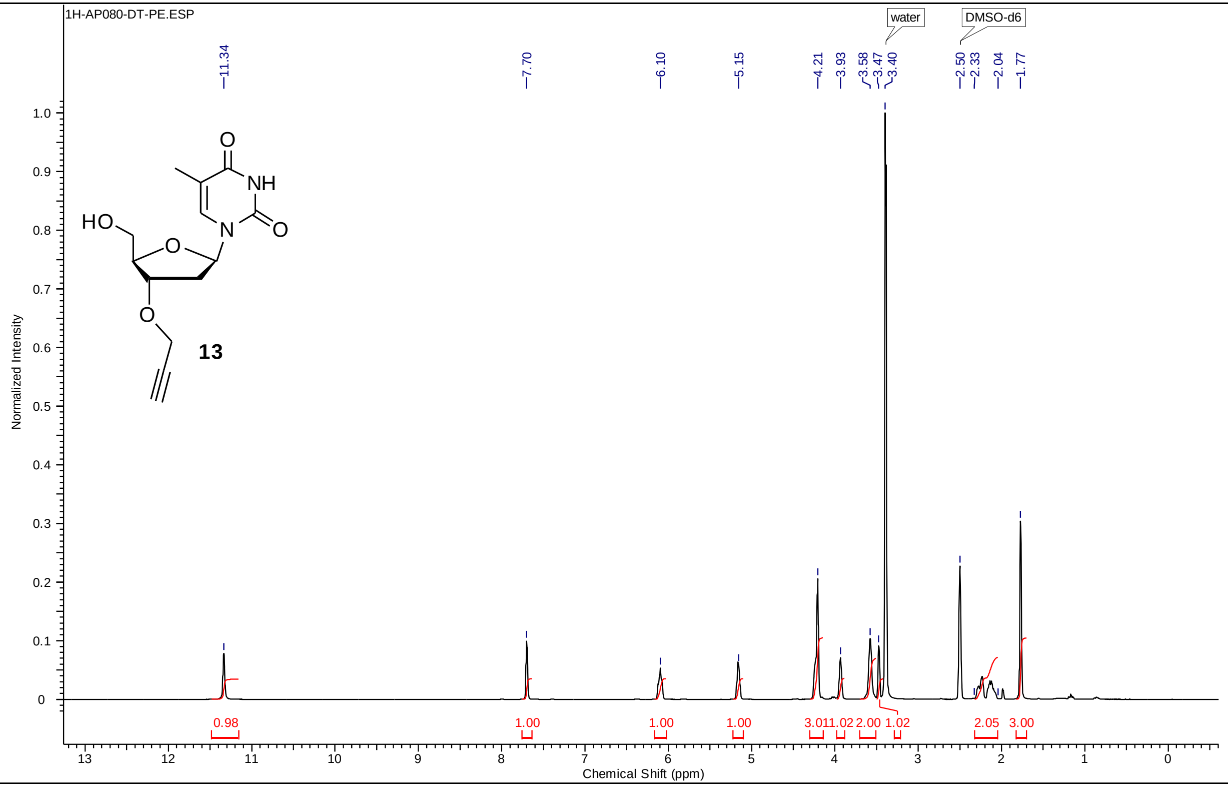

^{1}H-NMR spectrum of 1-((2*R*,4*S*,5*R*)-5-(hydroxymethyl)-4-(prop-2-ynyloxy)tetrahydrofuran-2-yl)-5-methylpyrimidine-2,4(1*H*,3*H*)-dione **13**

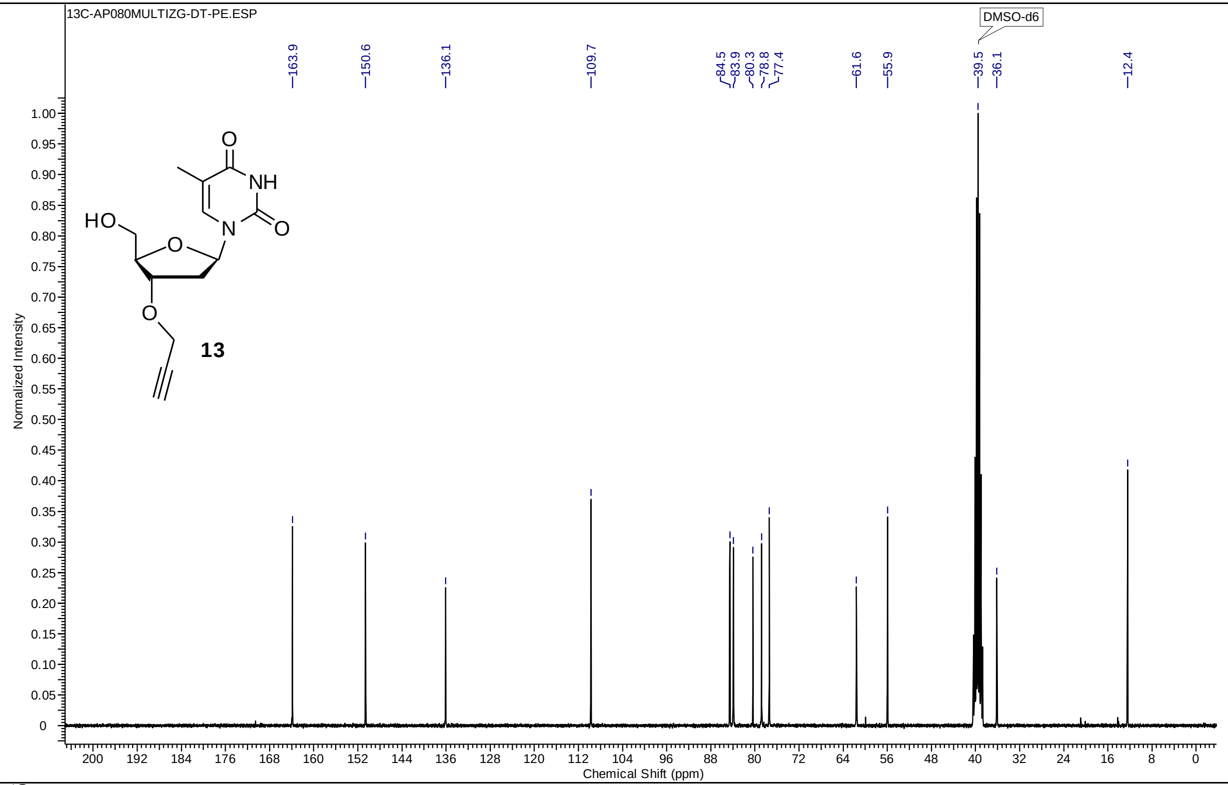

^{13}C-NMR spectrum of 1-((2*R*,4*S*,5*R*)-5-(hydroxymethyl)-4-(prop-2-ynyloxy)tetrahydrofuran-2-yl)-5-methylpyrimidine-2,4(1*H*,3*H*)-dione **13**

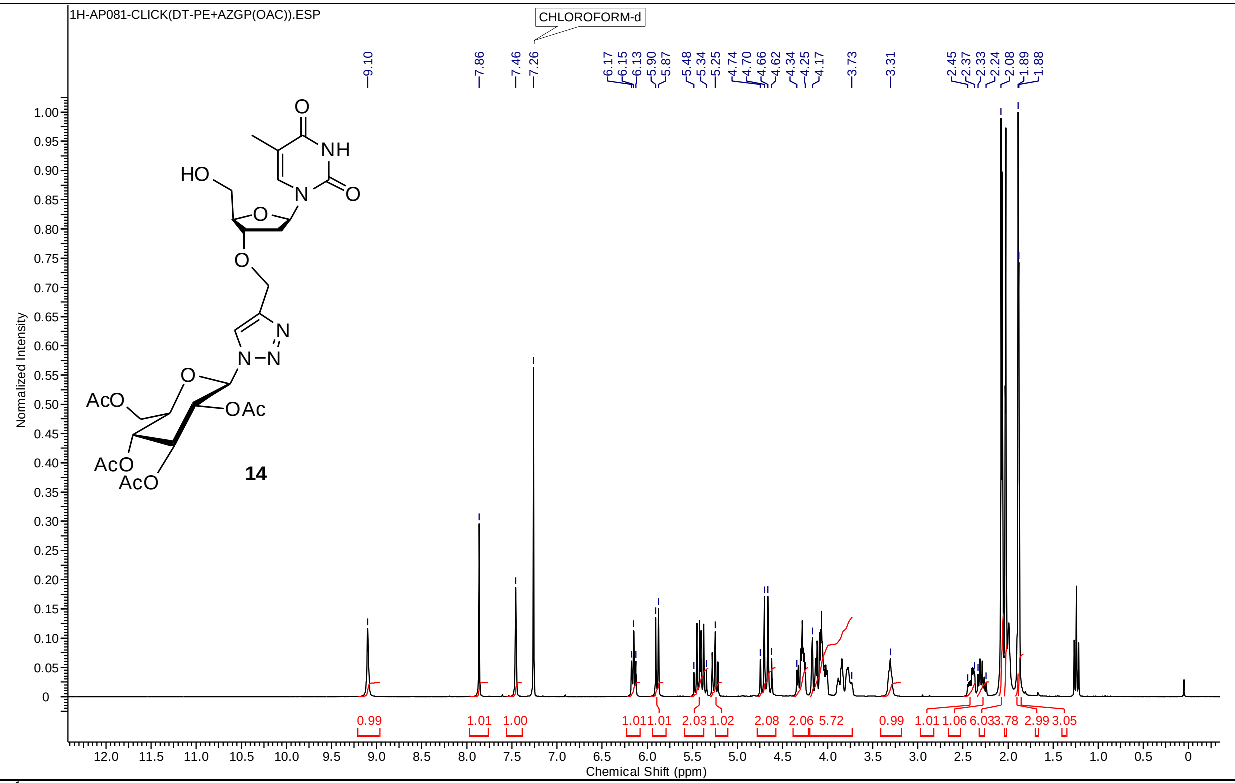

^{1}H-NMR spectrum of (2*R*,3*R*,4*S*,5*R*,6*R*)-2-(acetoxymethyl)-6-(4-(((2*R*,3*S*,5*R*)-2-(hydroxymethyl)-5-(5-methyl-2,4-dioxo-3,4-dihydropyrimidin-1(2H)-yl)tetrahydrofuran-3-yloxy)methyl)-1*H*-1,2,3-triazol-1-yl)tetrahydro-2*H*-pyran-3,4,5-triyl triacetate **14**

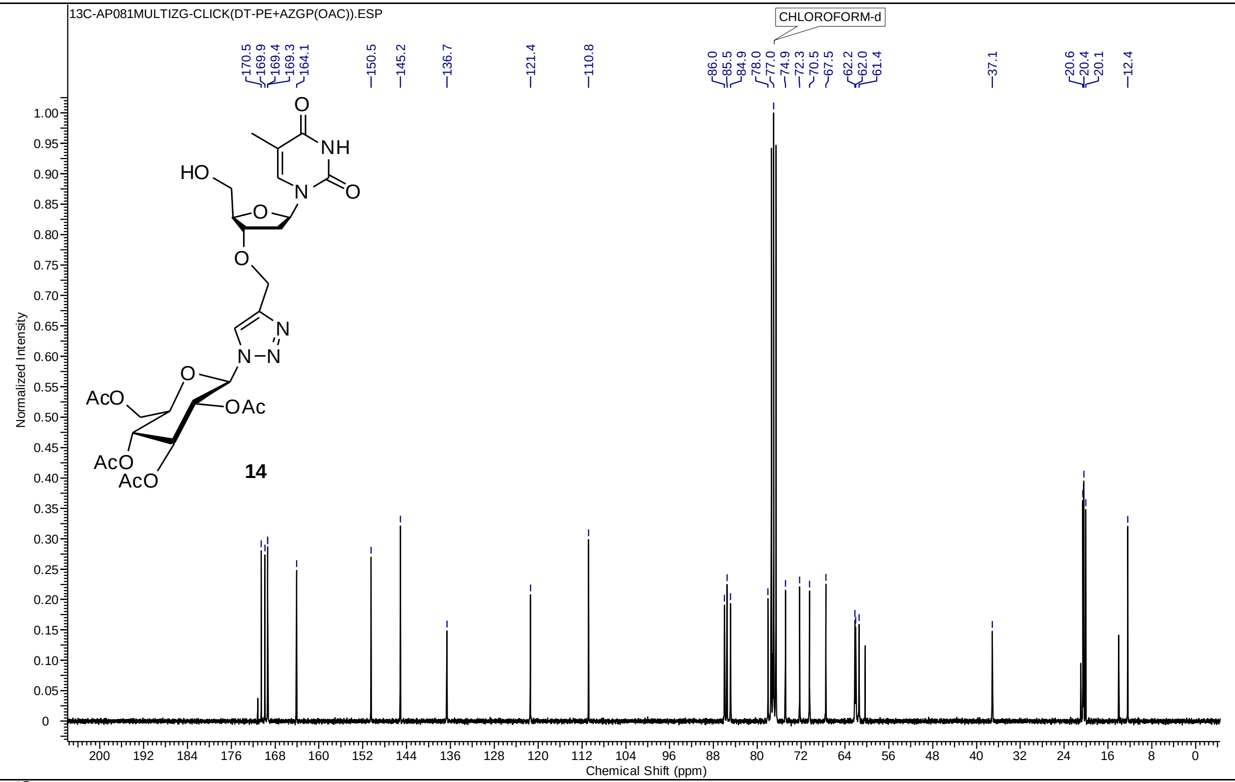

^{13}C-NMR spectrum of (2*R*,3*R*,4*S*,5*R*,6*R*)-2-(acetoxymethyl)-6-(4-(((2*R*,3*S*,5*R*)-2-(hydroxymethyl)-5-(5-methyl-2,4-dioxo-3,4-dihydropyrimidin-1(2H)-yl)tetrahydrofuran-3-yloxy)methyl)-1*H*-1,2,3-triazol-1-yl)tetrahydro-2*H*-pyran-3,4,5-triyl triacetate **14**

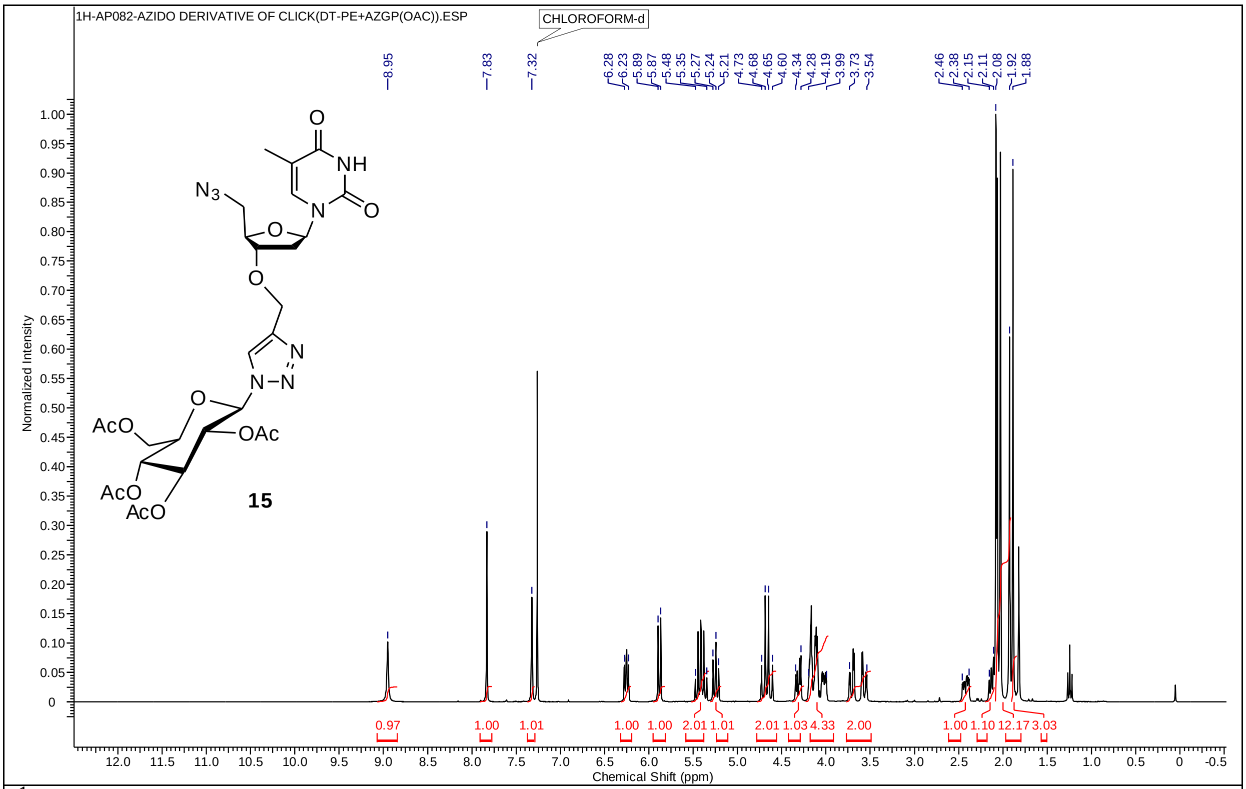

^{1}H-NMR spectrum of (2*R*,3*R*,4*S*,5*R*,6*R*)-2-(acetoxymethyl)-6-(4-(((2*R*,3*S*,5*R*)-2-(azidomethyl)-5-(5-methyl-2,4-dioxo-3,4-dihydropyrimidin-1(2*H*)-yl)tetrahydrofuran-3-yloxy)methyl)-1*H*-1,2,3-triazol-1-yl)tetrahydro-2*H*-pyran-3,4,5-triyl triacetate **15**

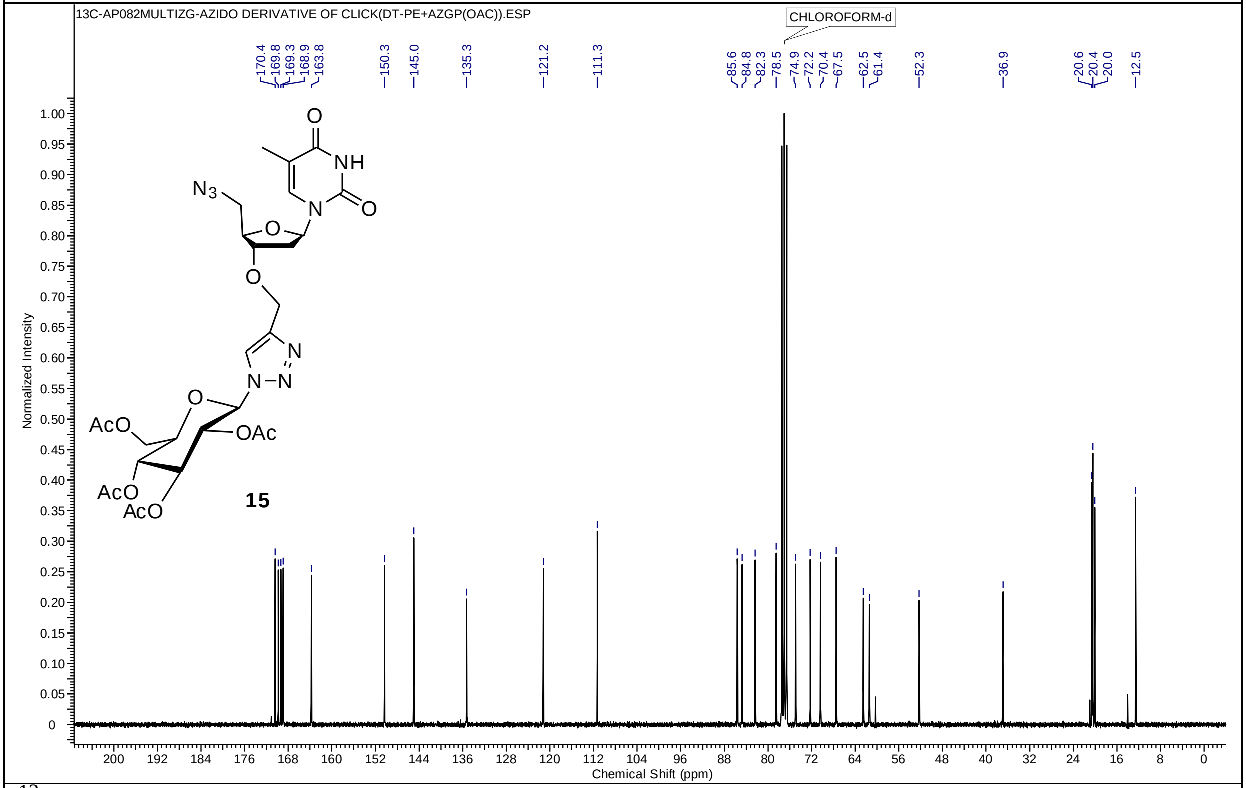

^{13}C-NMR spectrum of (2*R*,3*R*,4*S*,5*R*,6*R*)-2-(acetoxymethyl)-6-(4-(((2*R*,3*S*,5*R*)-2-(azidomethyl)-5-(5-methyl-2,4-dioxo-3,4-dihydropyrimidin-1(2*H*)-yl)tetrahydrofuran-3-yloxy)methyl)-1*H*-1,2,3-triazol-1-yl)tetrahydro-2*H*-pyran-3,4,5-triyl triacetate **15**

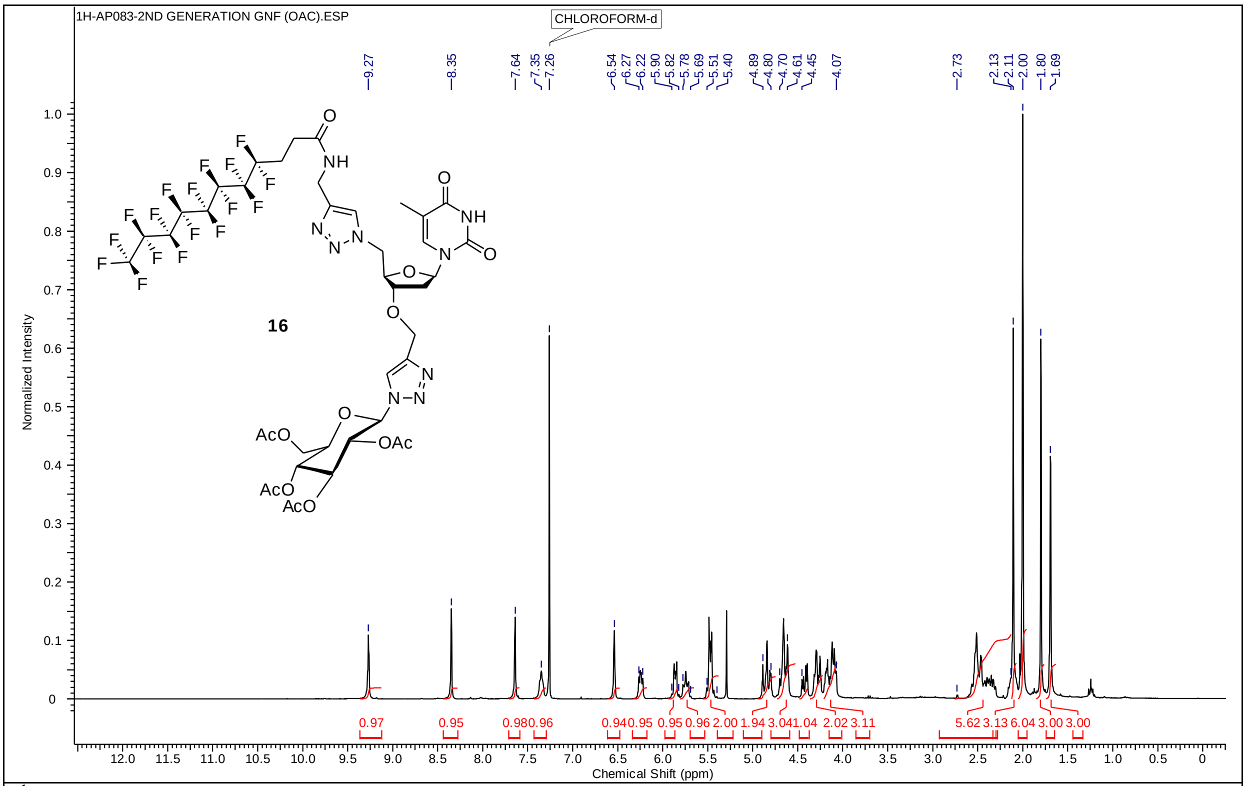

^{1}H-NMR spectrum of (2*R*,3*R*,4*S*,5*R*,6*R*)-2-(acetoxymethyl)-6-(4-(((2*R*,3*S*,5*R*)-2-((4-((4,4,5,5,6,6,7,7,8,8,9,9,10,10,11,11,11-heptadecafluoroundecanamido)methyl)-1*H*-1,2,3-triazol-1-yl)methyl)-5-(5-methyl-2,4-dioxo-3,4-dihydropyrimidin-1(2*H*)-yl)tetrahydrofuran-3-yloxy)methyl)-1*H*-1,2,3-triazol-1-yl)tetrahydro-2*H*-pyran-3,4,5-triyl triacetate **16**

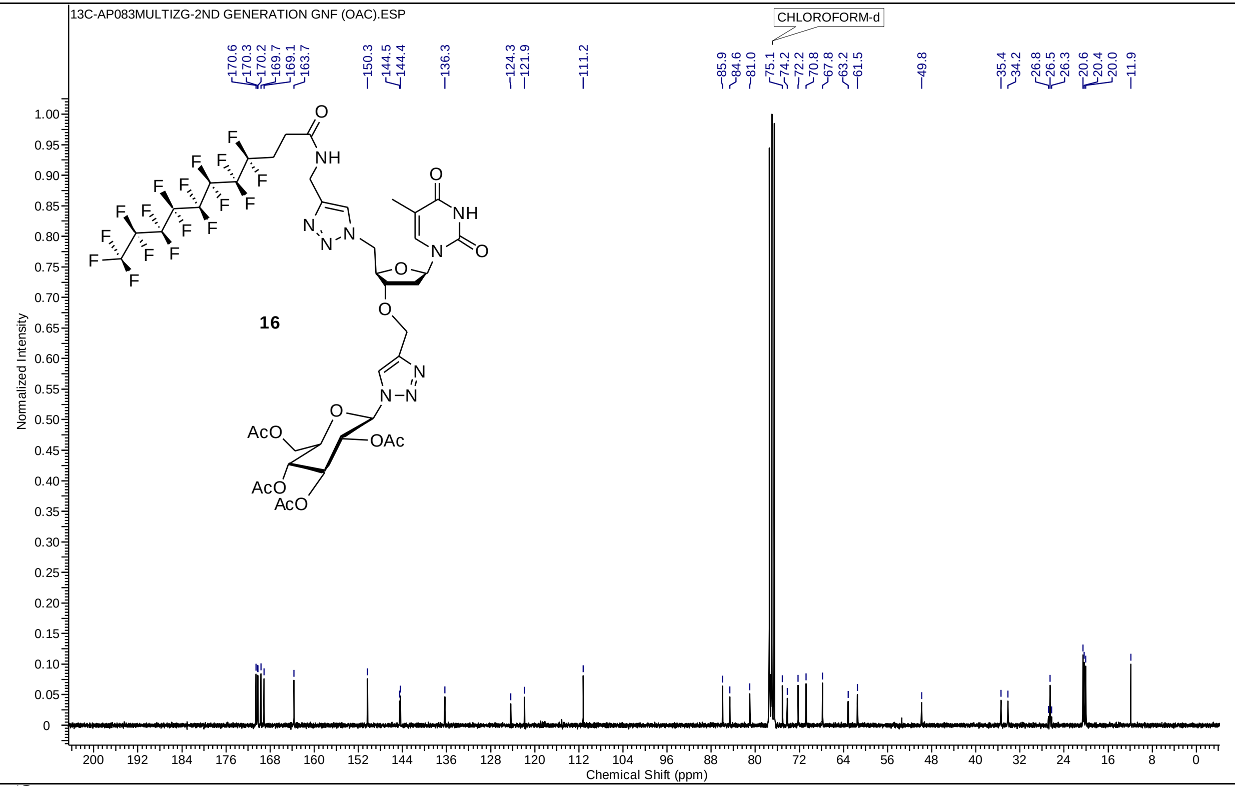

^{13}C-NMR spectrum of (2*R*,3*R*,4*S*,5*R*,6*R*)-2-(acetoxymethyl)-6-(4-(((2*R*,3*S*,5*R*)-2-((4-((4,4,5,5,6,6,7,7,8,8,9,9,10,10,11,11,11-heptadecafluoroundecanamido)methyl)-1*H*-1,2,3-triazol-1-yl)methyl)-5-(5-methyl-2,4-dioxo-3,4-dihydropyrimidin-1(2*H*)-yl)tetrahydrofuran-3-yloxy)methyl)-1*H*-1,2,3-triazol-1-yl)tetrahydro-2*H*-pyran-3,4,5-triyl triacetate **16**

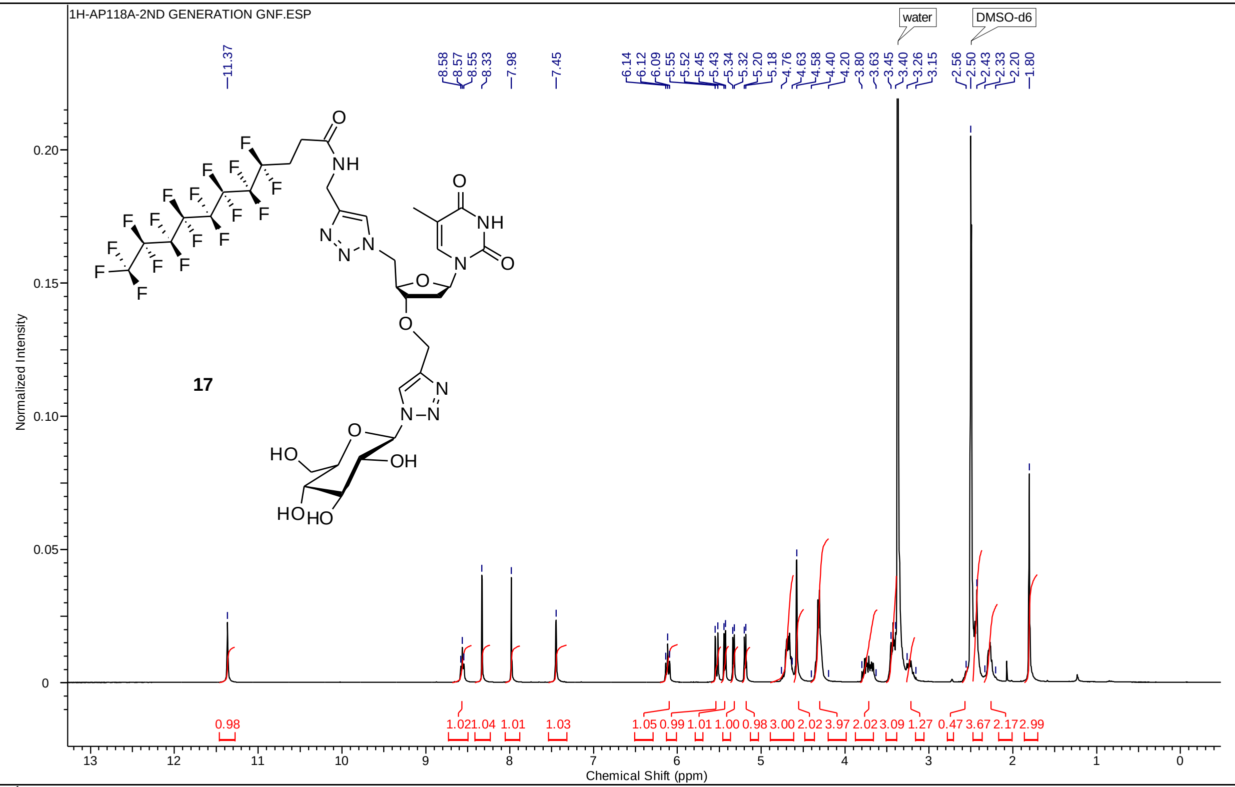

^{1}H-NMR spectrum of 4,4,5,5,6,6,7,7,8,8,9,9,10,10,11,11,11-heptadecafluoro-*N*-((1-(((2*R*,3*S*,5*R*)-5-(5-methyl-2,4-dioxo-3,4-dihydropyrimidin-1(2*H*)-yl)-3-((1-((2*R*,3*R*,4*S*,5*S*,6*R*)-3,4,5-trihydroxy-6-(hydroxymethyl)tetrahydro-2*H*-pyran-2-yl)-1*H*-1,2,3-triazol-4-yl)methoxy) tetrahydrofuran-2-yl)methyl)-1*H*-1,2,3-triazol-4-yl)methyl)undecanamide **17**

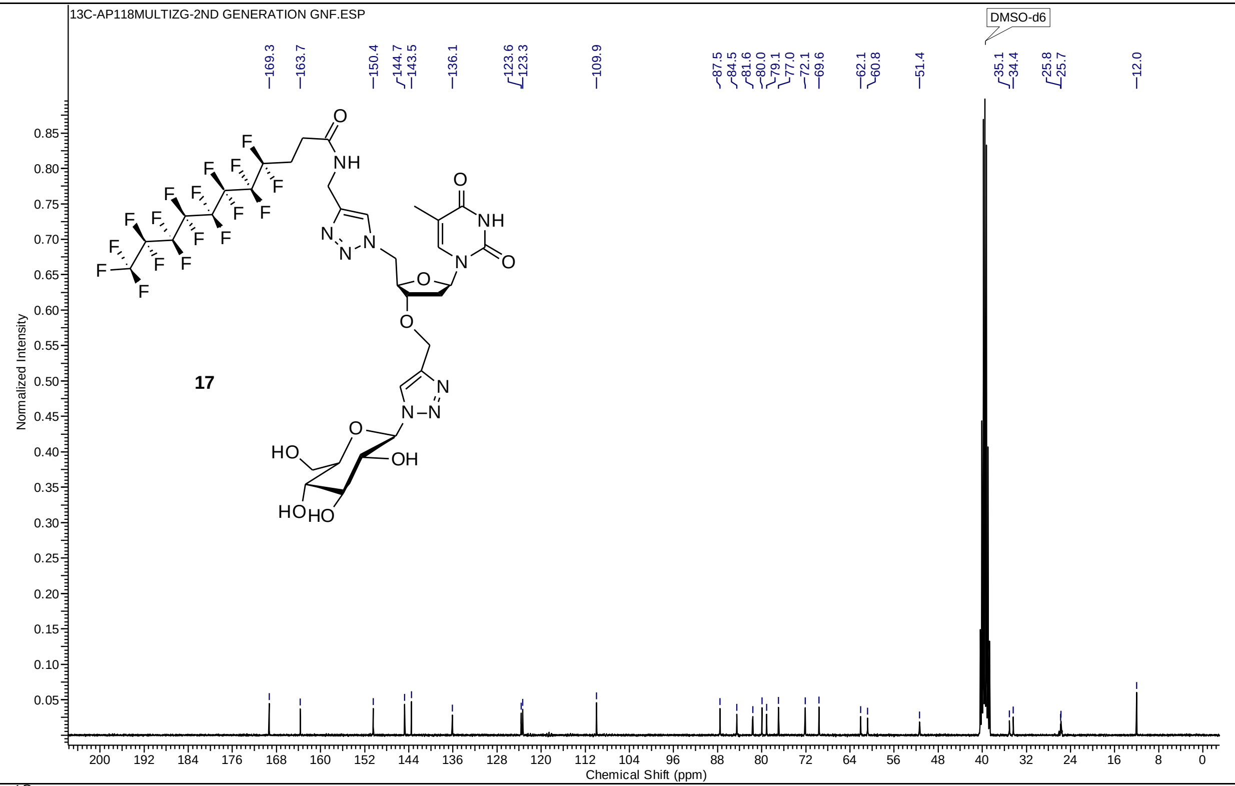

^{13}C-NMR spectrum of 4,4,5,5,6,6,7,7,8,8,9,9,10,10,11,11,11-heptadecafluoro-*N*-((1-(((2*R*,3*S*,5*R*)-5-(5-methyl-2,4-dioxo-3,4-dihydropyrimidin-1(2*H*)-yl)-3-((1-((2*R*,3*R*,4*S*,5*S*,6*R*)-3,4,5-trihydroxy-6-(hydroxymethyl)tetrahydro-2*H*-pyran-2-yl)-1*H*-1,2,3-triazol-4-yl)methoxy) tetrahydrofuran-2-yl)methyl)-1*H*-1,2,3-triazol-4-yl)methyl)undecanamide **17**

II. Mass Spectra (HR ESI-MS)

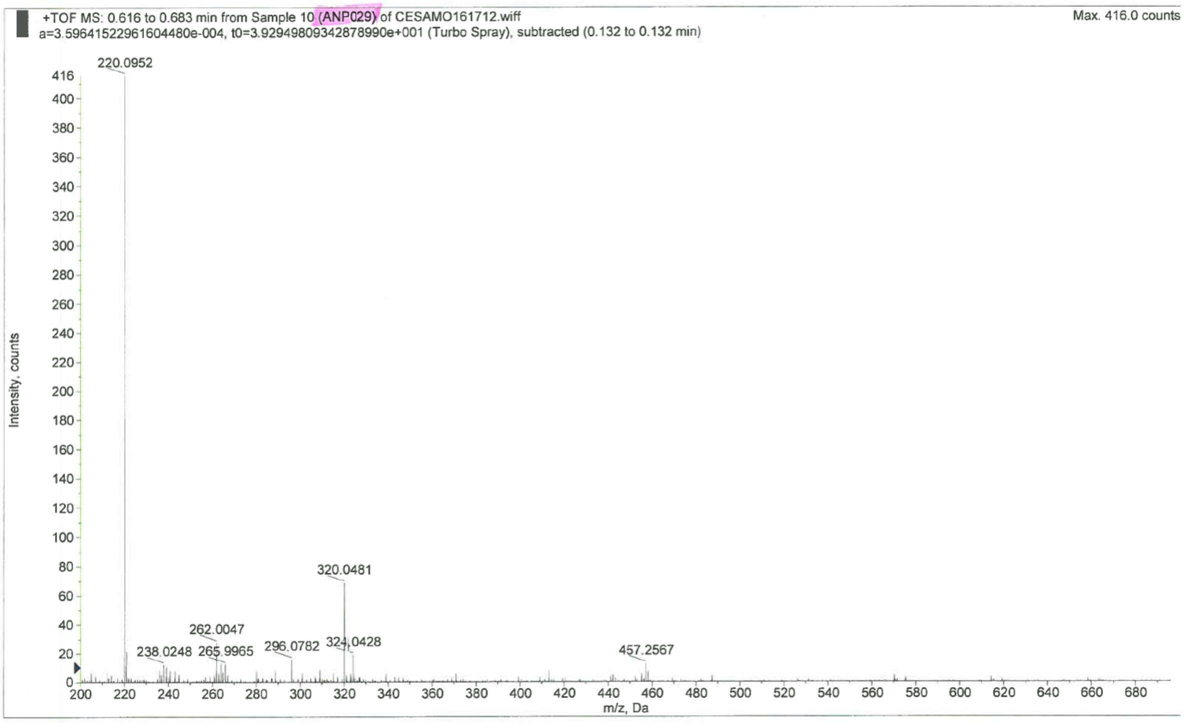

Mass spectrum of (*S*)-2-(2,2-dimethyl-1,3-dioxolan-4-yl)-*N*-(prop-2-ynyl)acetamide **4**

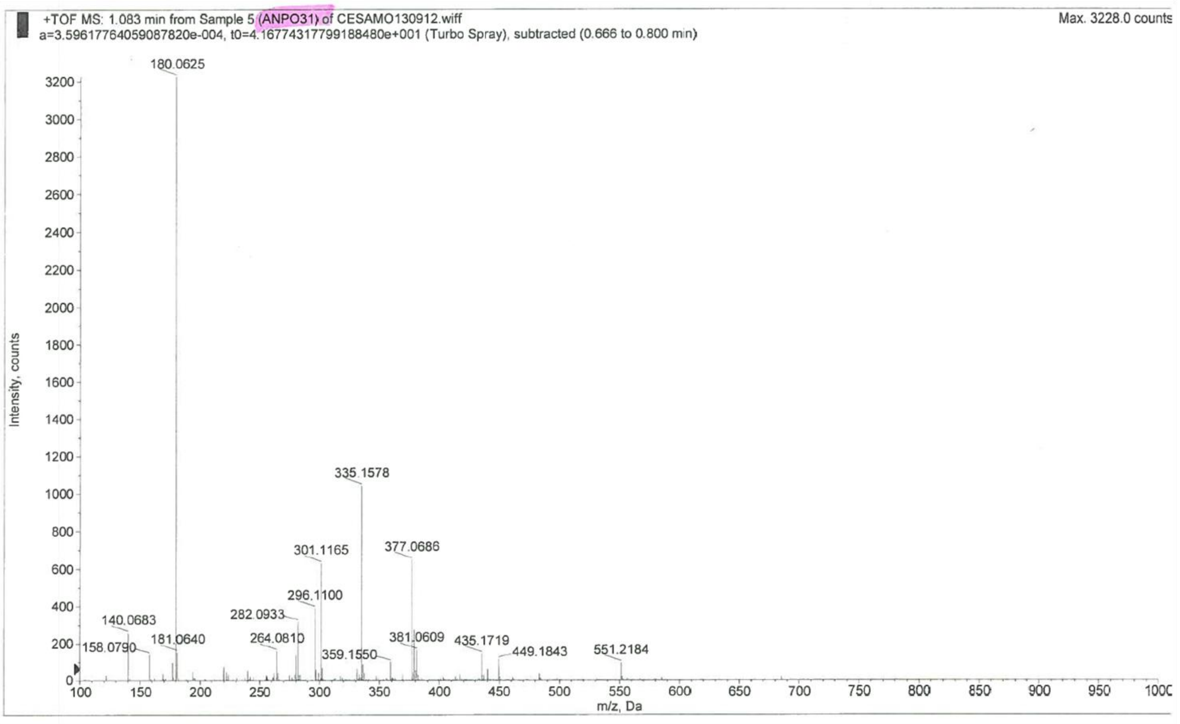

Mass spectrum of (*S*)-3,4-dihydroxy-*N*-(prop-2-ynyl)butanamide **5**

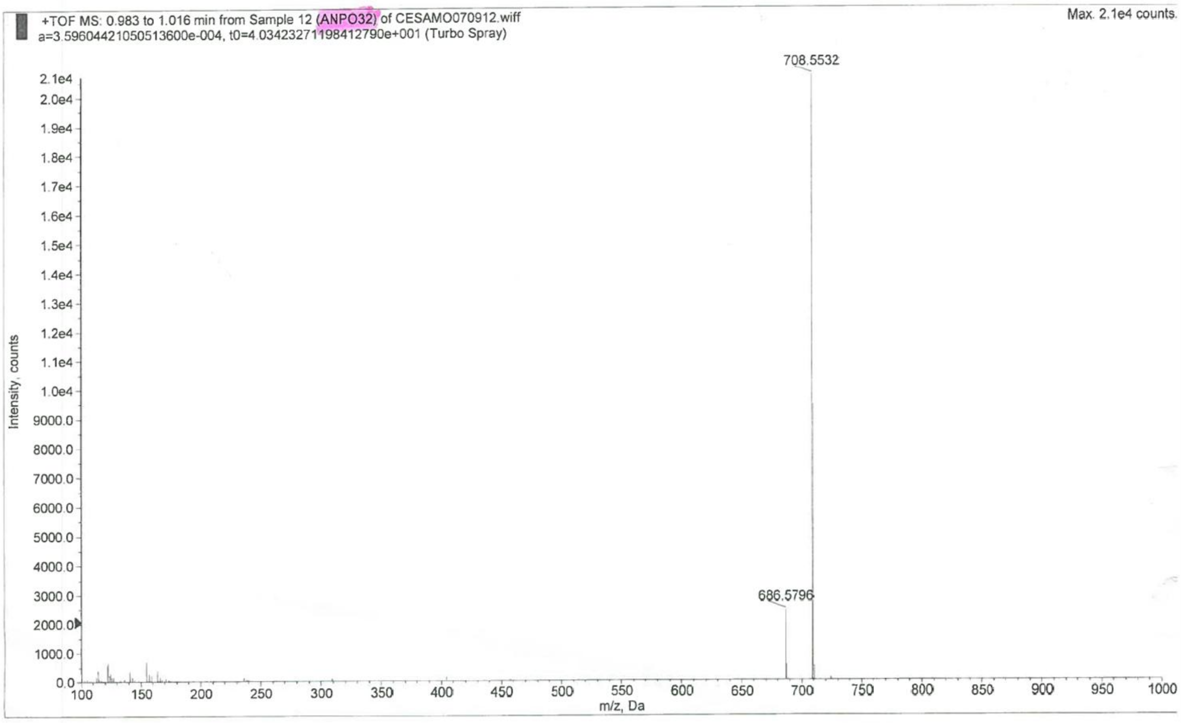

Mass spectrum of (*Z*)-((*S*)-4-oxo-4-(prop-2-ynylamino)butane-1,2-diyl) dioleate **6a**

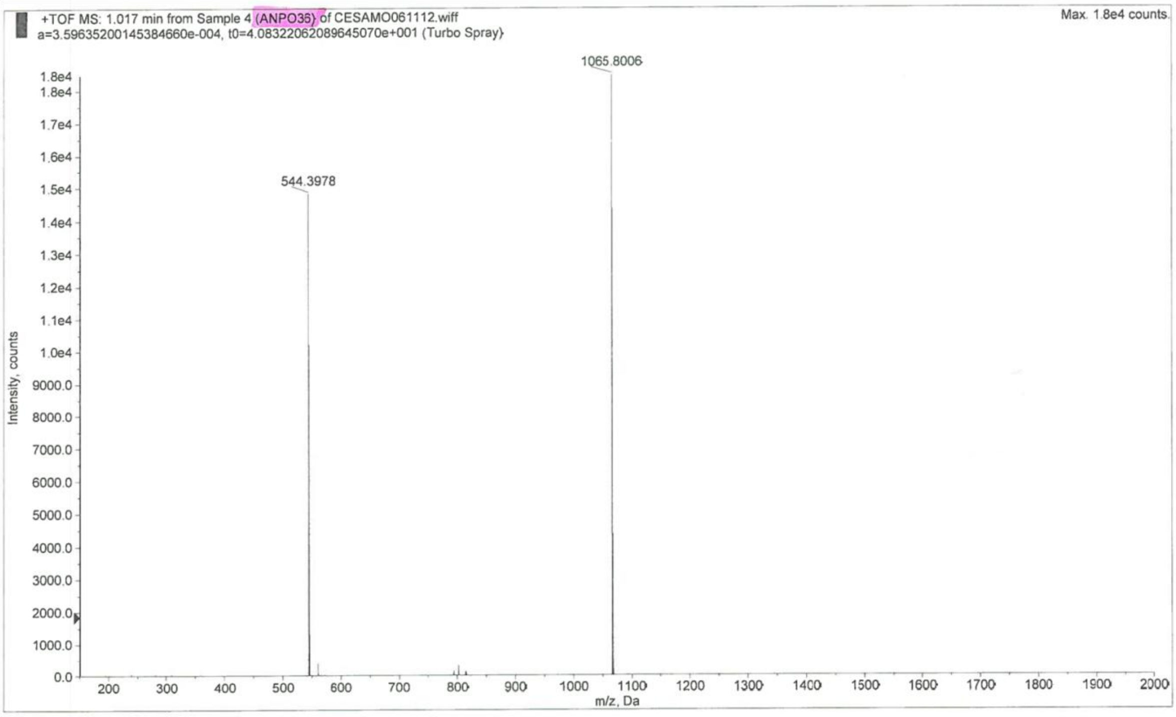

Mass spectrum of (*S*)-4-oxo-4-(prop-2-ynylamino)butane-1,2-diyl didodecanoate **6b**

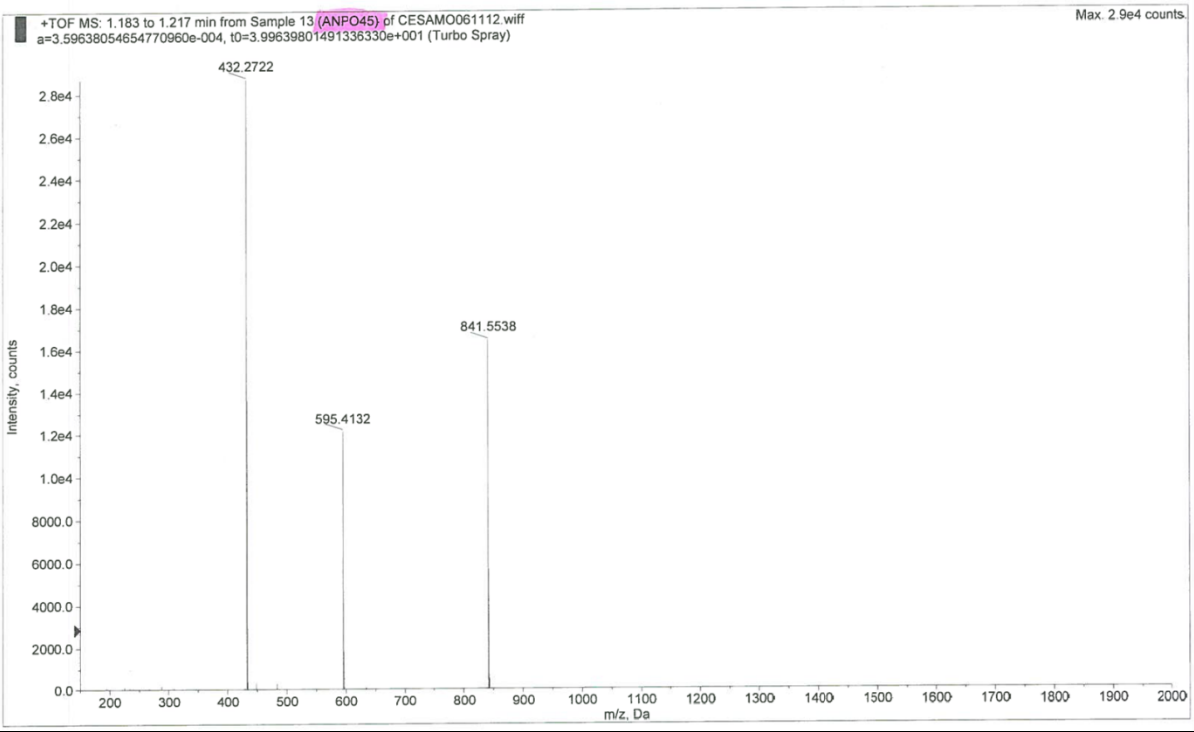

Mass spectrum of (*S*)-4-oxo-4-(prop-2-ynylamino)butane-1,2-diyl dioctanoate **6c**

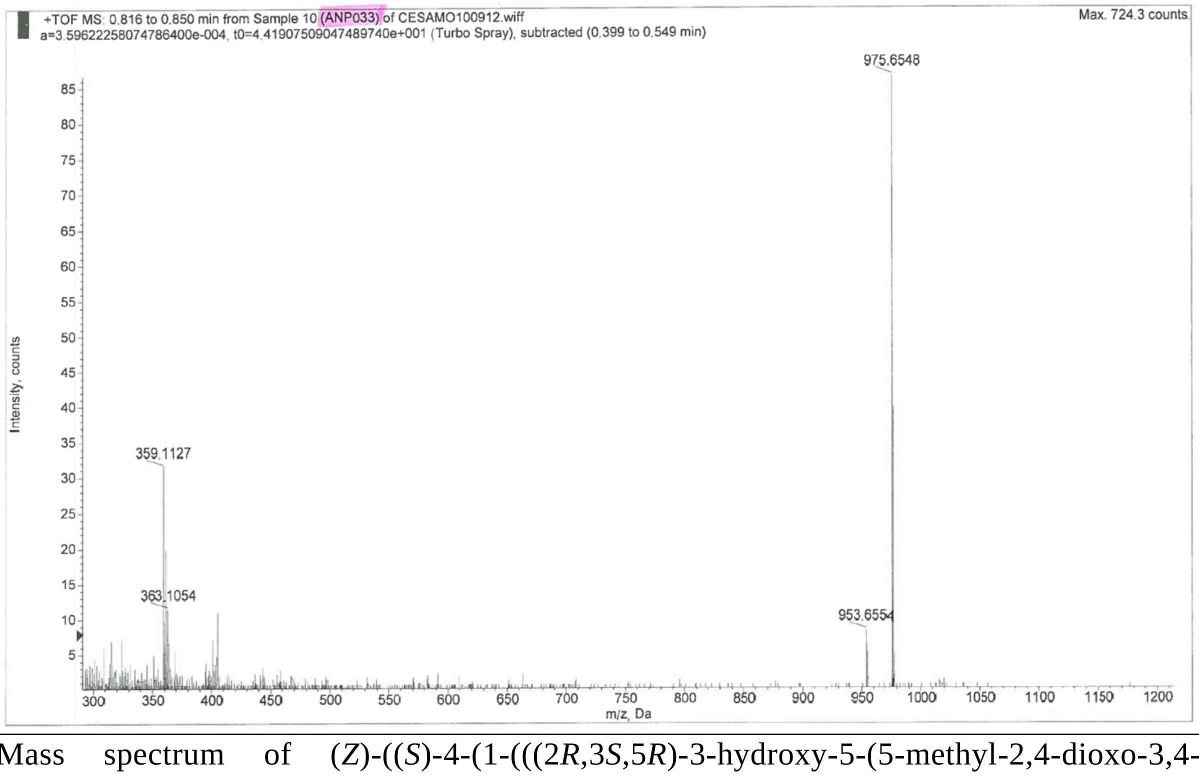

Mass spectrum of (*Z*)-((*S*)-4-(1-(((2*R*,3*S*,5*R*)-3-hydroxy-5-(5-methyl-2,4-dioxo-3,4-dihydropyrimidin-1(2*H*)-yl)tetrahydrofuran-2-yl)methyl)-1*H*-1,2,3-triazol-4-ylamino)-4-oxobutane-1,2-diyl) dioleate **7a**

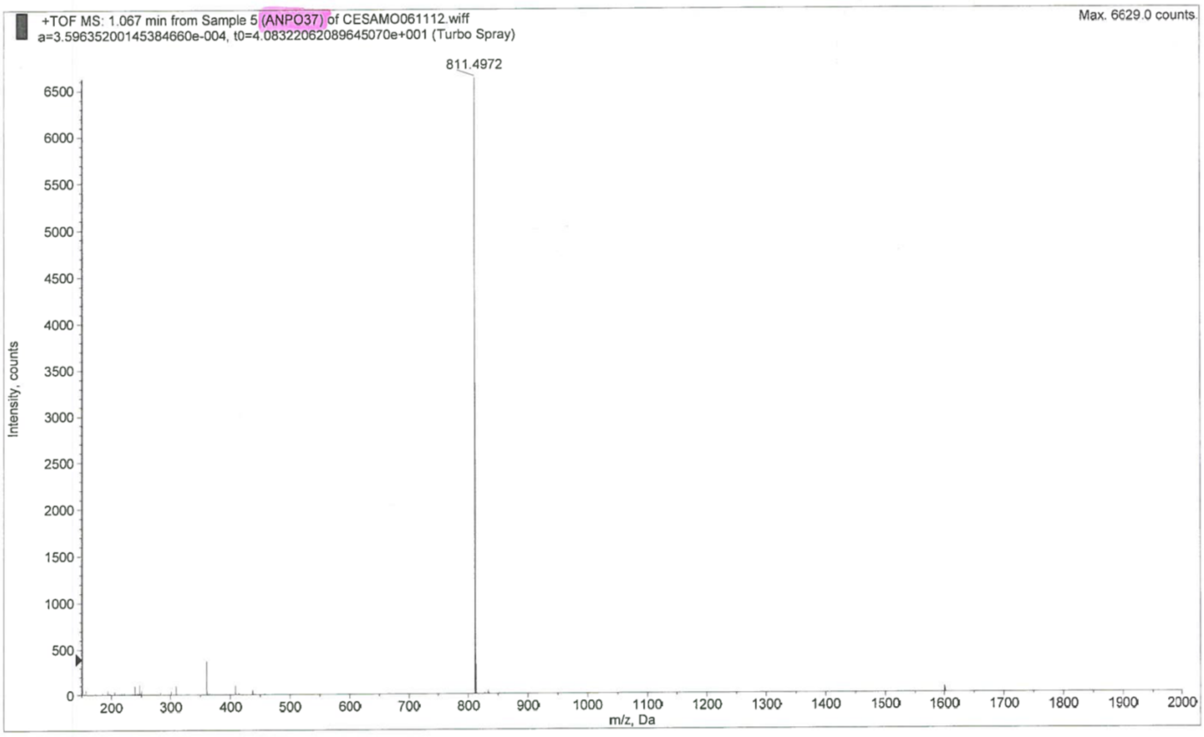

Mass spectrum of (*S*)-4-(1-(((2*R*,3*S*,5*R*)-3-hydroxy-5-(5-methyl-2,4-dioxo-3,4-dihydropyrimidin-1(2*H*)-yl)tetrahydrofuran-2-yl)methyl)-1*H*-1,2,3-triazol-4-ylamino)-4-oxobutane-1,2-diyl didodecanoate **7b**

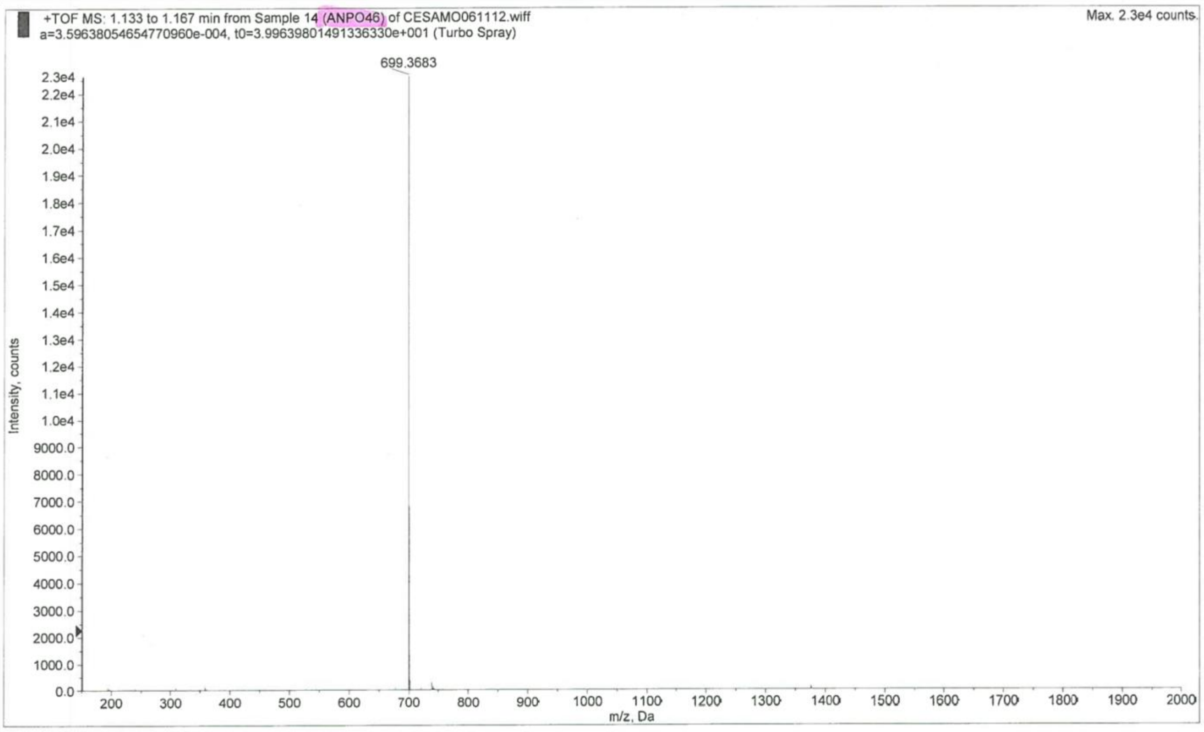

Mass spectrum of (*S*)-4-(1-(((2*R*,3*S*,5*R*)-3-hydroxy-5-(5-methyl-2,4-dioxo-3,4-dihydropyrimidin-1(2*H*)-yl)tetrahydrofuran-2-yl)methyl)-1*H*-1,2,3-triazol-4-ylamino)-4-oxobutane-1,2-diyl dioctanoate **7c**

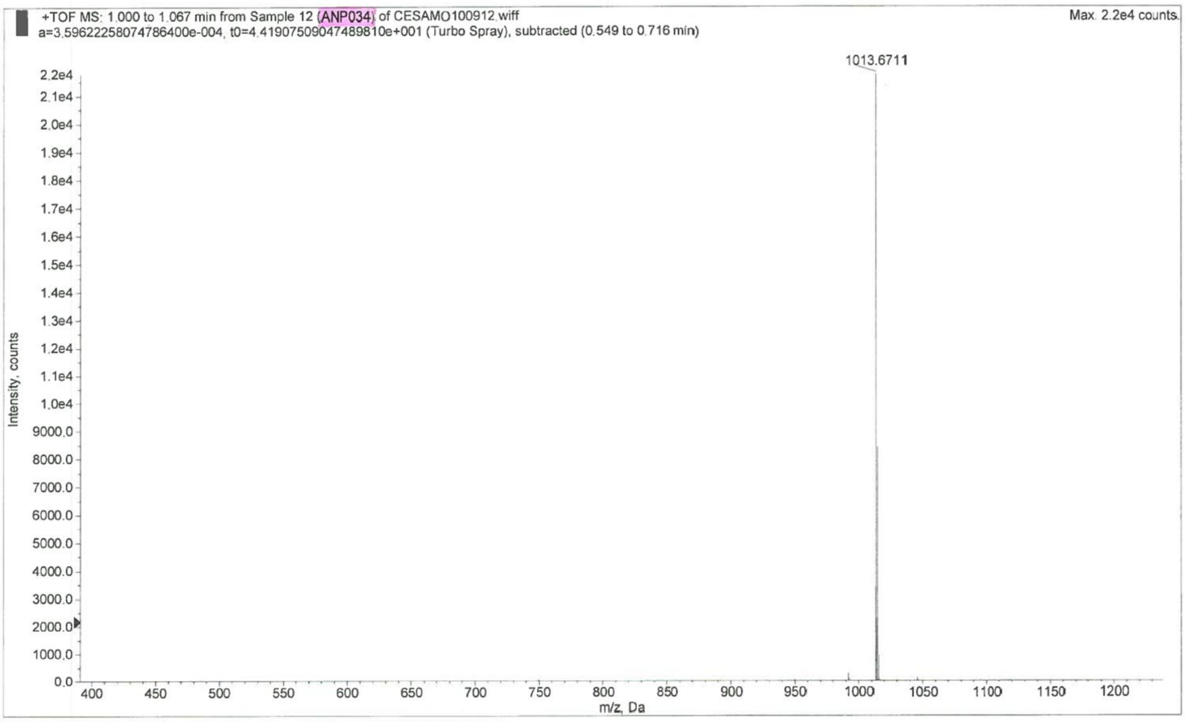

Mass spectrum of (*Z*)-((*S*)-4-(1-(((2*R*,3*S*,5*R*)-3-hydroxy-5-(5-methyl-2,4-dioxo-3-(prop-2-ynyl)-3,4-dihydropyrimidin-1(2*H*)-yl)tetrahydrofuran-2-yl)methyl)-1*H*-1,2,3-triazol-4-ylamino)-4-oxobutane-1,2-diyl) dioleate **8a**

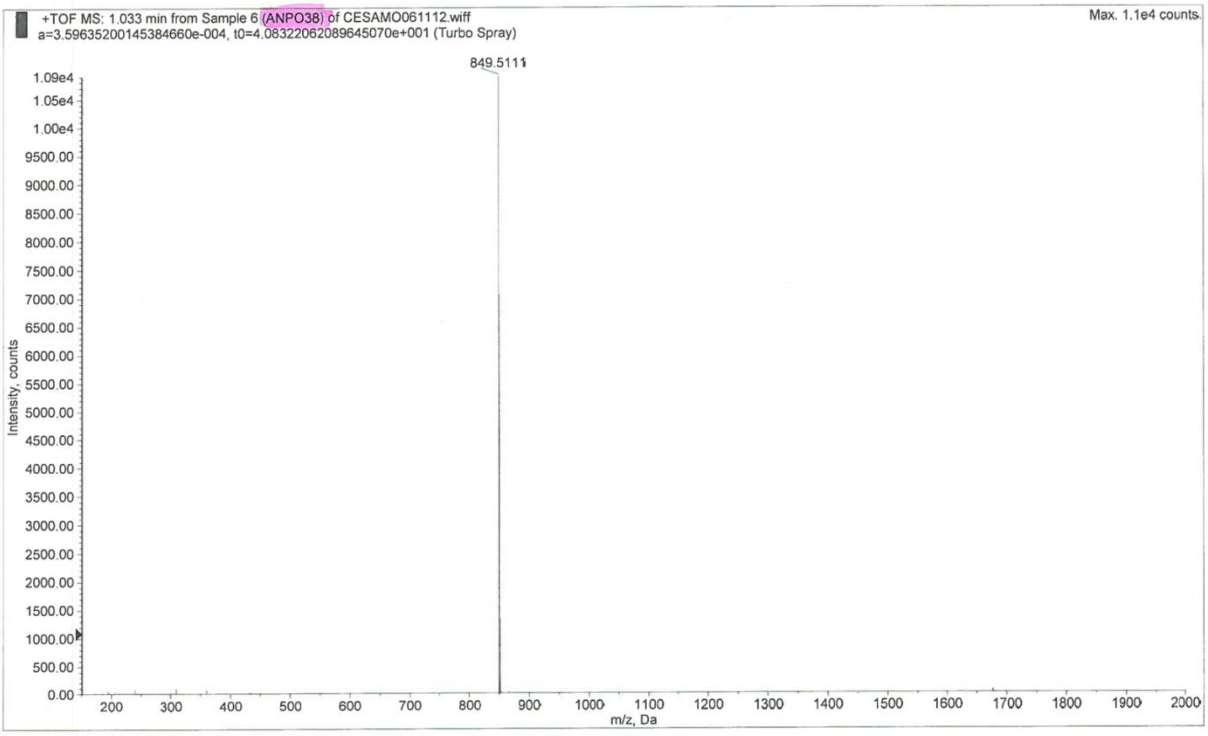

Mass spectrum of (*S*)-4-(1-(((2*R*,3*S*,5*R*)-3-hydroxy-5-(5-methyl-2,4-dioxo-3-(prop-2-ynyl)-3,4-dihydropyrimidin-1(2*H*)-yl)tetrahydrofuran-2-yl)methyl)-1*H*-1,2,3-triazol-4-ylamino)-4-oxobutane-1,2-diyl didodecanoate **8b**

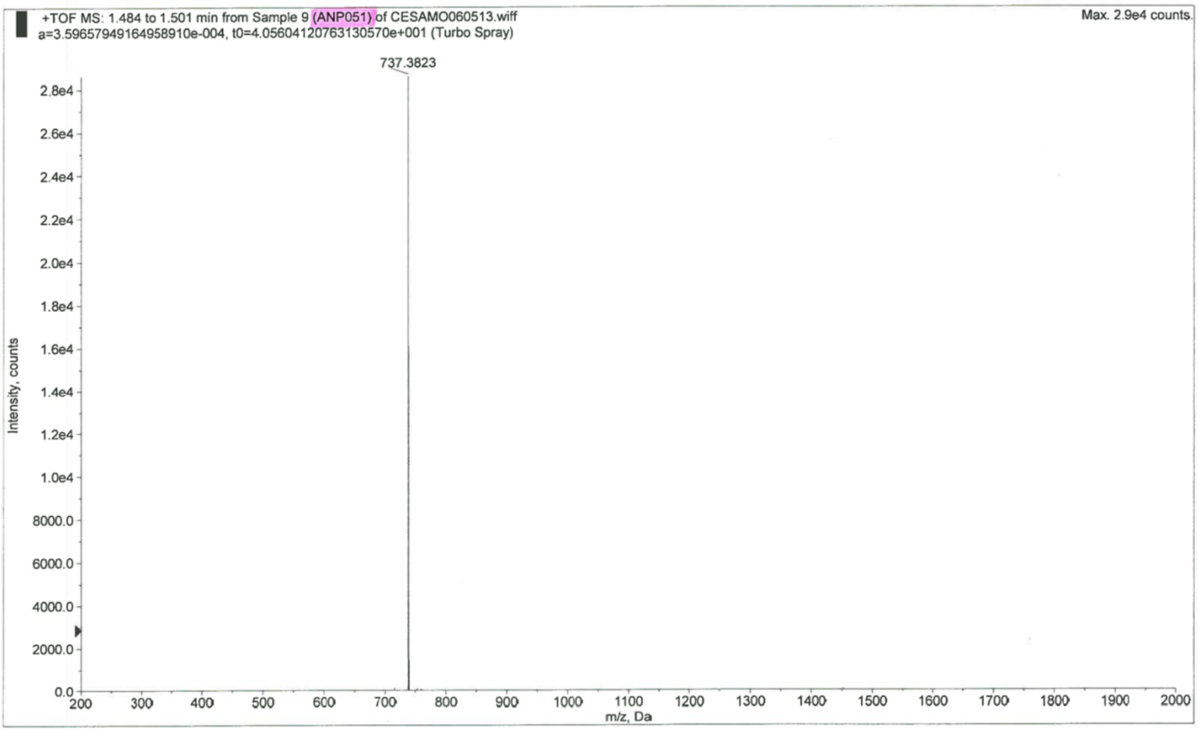

Mass spectrum of (*S*)-4-(1-(((2*R*,3*S*,5*R*)-3-hydroxy-5-(5-methyl-2,4-dioxo-3-(prop-2-ynyl)-3,4-dihydropyrimidin-1(2*H*)-yl)tetrahydrofuran-2-yl)methyl)-1*H*-1,2,3-triazol-4-ylamino)-4-oxobutane-1,2-diyl dioctanoate **8c**

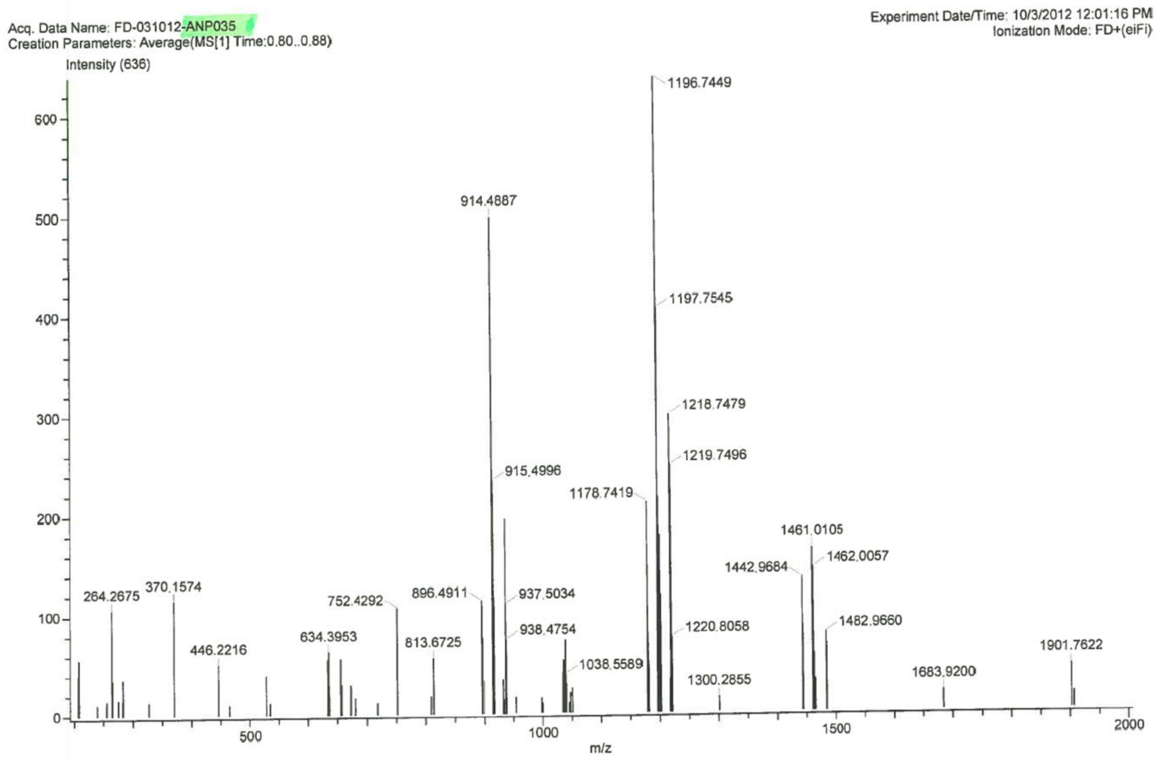

Mass spectrum of (*Z*)-4-(1-(((2*R*,3*S*,5*R*)-3-hydroxy-5-(5-methyl-2,4-dioxo-3-((1-((2*R*,3*R*,4*S*,5*S*,6*R*)-3,4,5-trihydroxy-6-(hydroxymethyl)tetrahydro-2*H*-pyran-2-yl)-1*H*-1,2,3-triazol-4-yl)methyl)-3,4-dihydropyrimidin-1(2*H*)-yl)tetrahydrofuran-2-yl)methyl)-1*H*-1,2,3-triazol-4-ylamino)-4-oxobutane-1,2-diyl dioleate **9a**

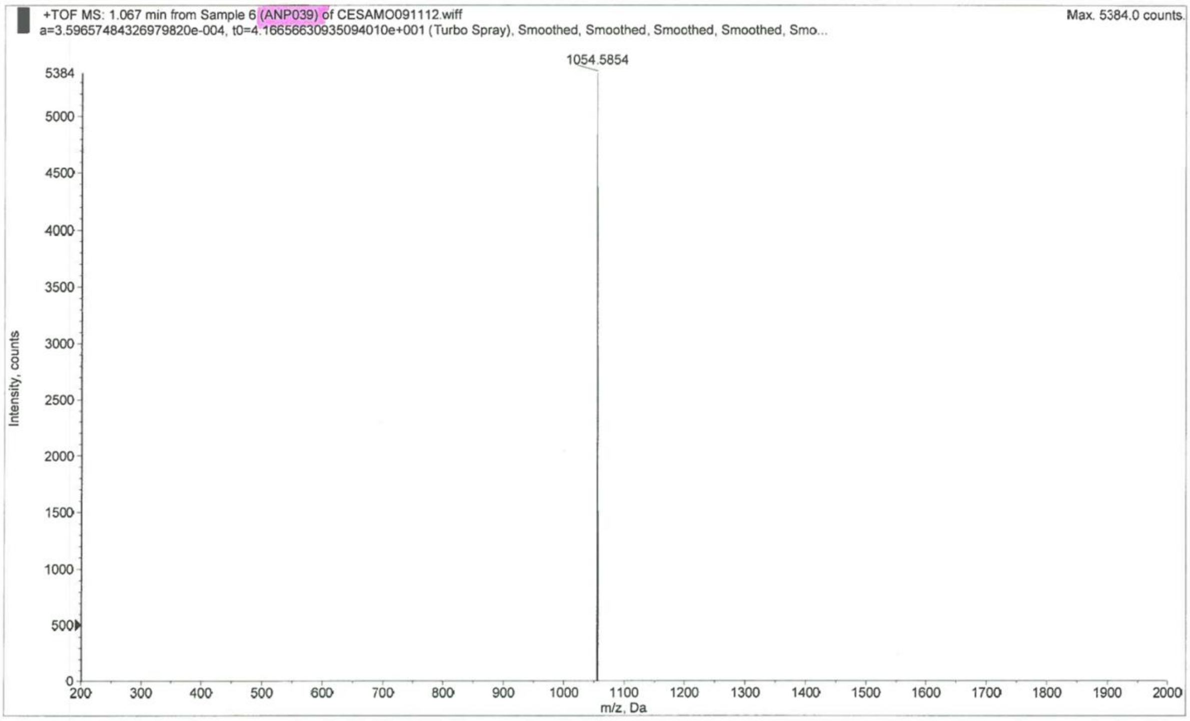

Mass spectrum of 4-(1-(((2*R*,3*S*,5*R*)-3-hydroxy-5-(5-methyl-2,4-dioxo-3-((1-((2*R*,3*R*,4*S*,5*S*,6*R*)-3,4,5-trihydroxy-6-(hydroxymethyl)tetrahydro-2*H*-pyran-2-yl)-1H-1,2,3-triazol-4-yl)methyl)-3,4-dihydropyrimidin-1(2*H*)-yl)tetrahydrofuran-2-yl)methyl)-1*H*-1,2,3-triazol-4-ylamino)-4-oxobutane-1,2-diyl didodecanoate **9b**

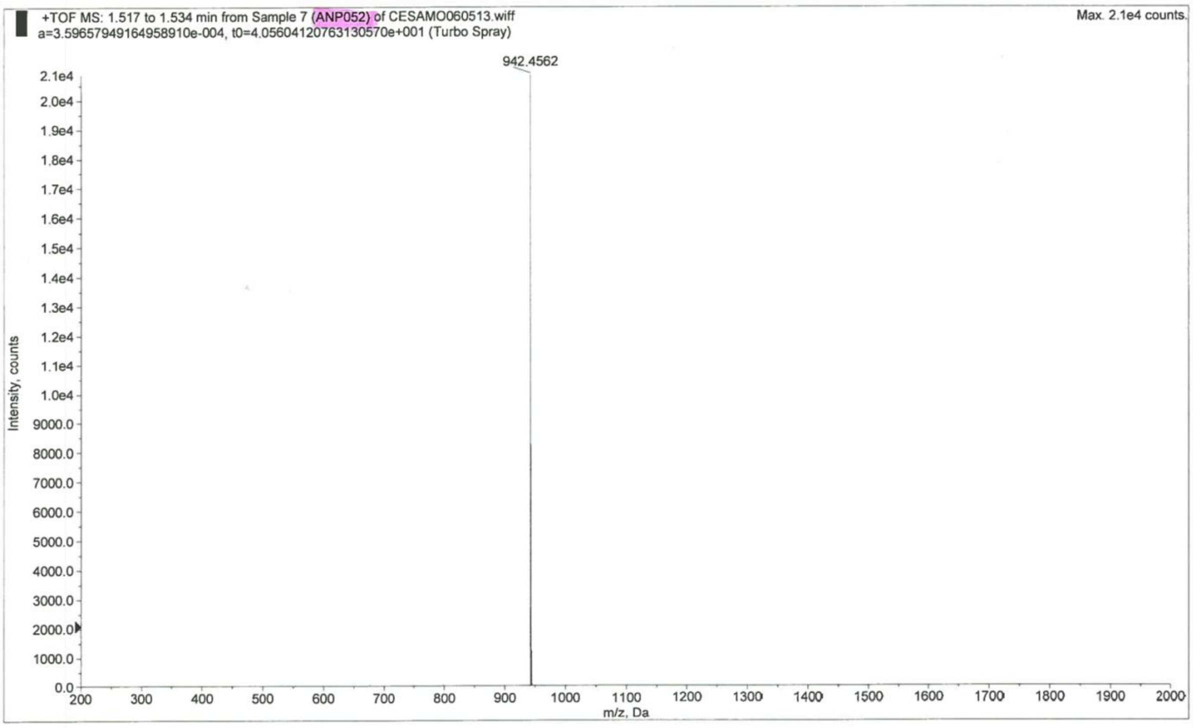

Mass spectrum of 4-(1-(((2*R*,3*S*,5*R*)-3-hydroxy-5-(5-methyl-2,4-dioxo-3-((1-((2*R*,3*R*,4*S*,5*S*,6*R*)-3,4,5-trihydroxy-6-(hydroxymethyl)tetrahydro-2H-pyran-2-yl)-1*H*-1,2,3-triazol-4-yl)methyl)-3,4-dihydropyrimidin-1(2*H*)-yl)tetrahydrofuran-2-yl)methyl)-1*H*-1,2,3-triazol-4-ylamino)-4-oxobutane-1,2-diyl dioctanoate **9c**

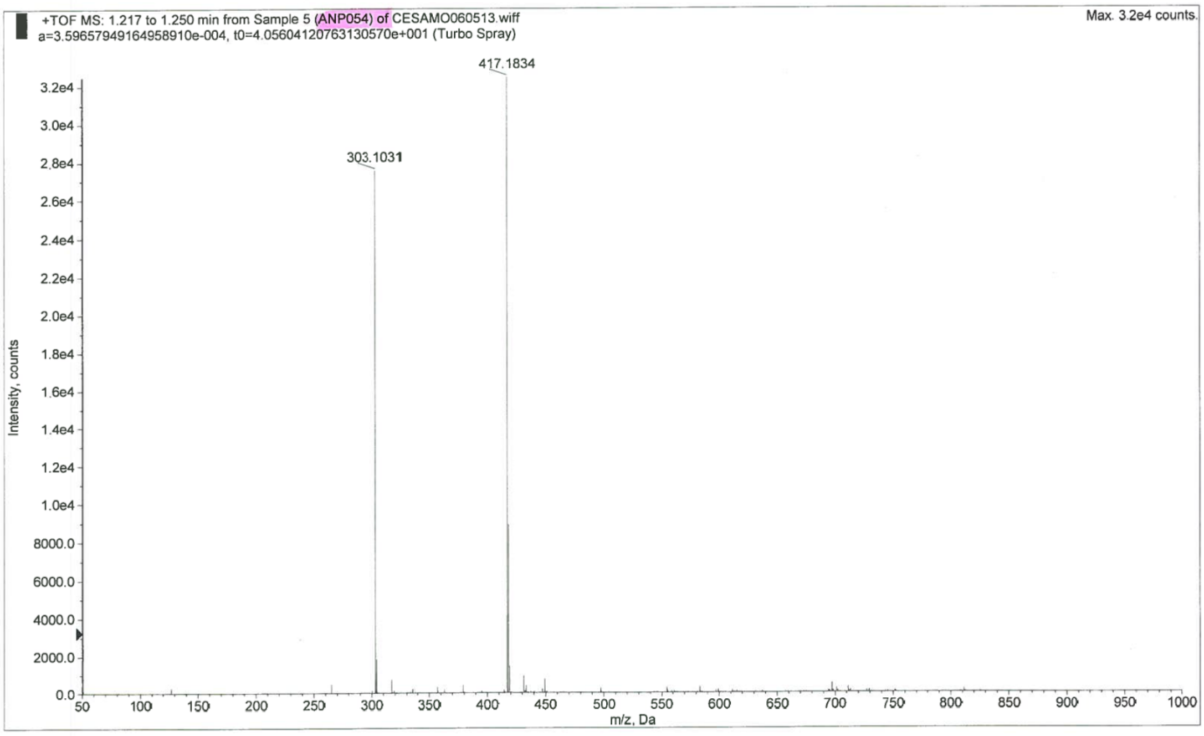

Mass spectrum of 1-((2*R*,4*S*,5*R*)-5-((tert-butyldimethylsilyloxy)methyl)-4-(prop-2-ynyloxy) tetrahydrofuran-2-yl)-5-methylpyrimidine-2,4(1*H*,3*H*)-dione **12**

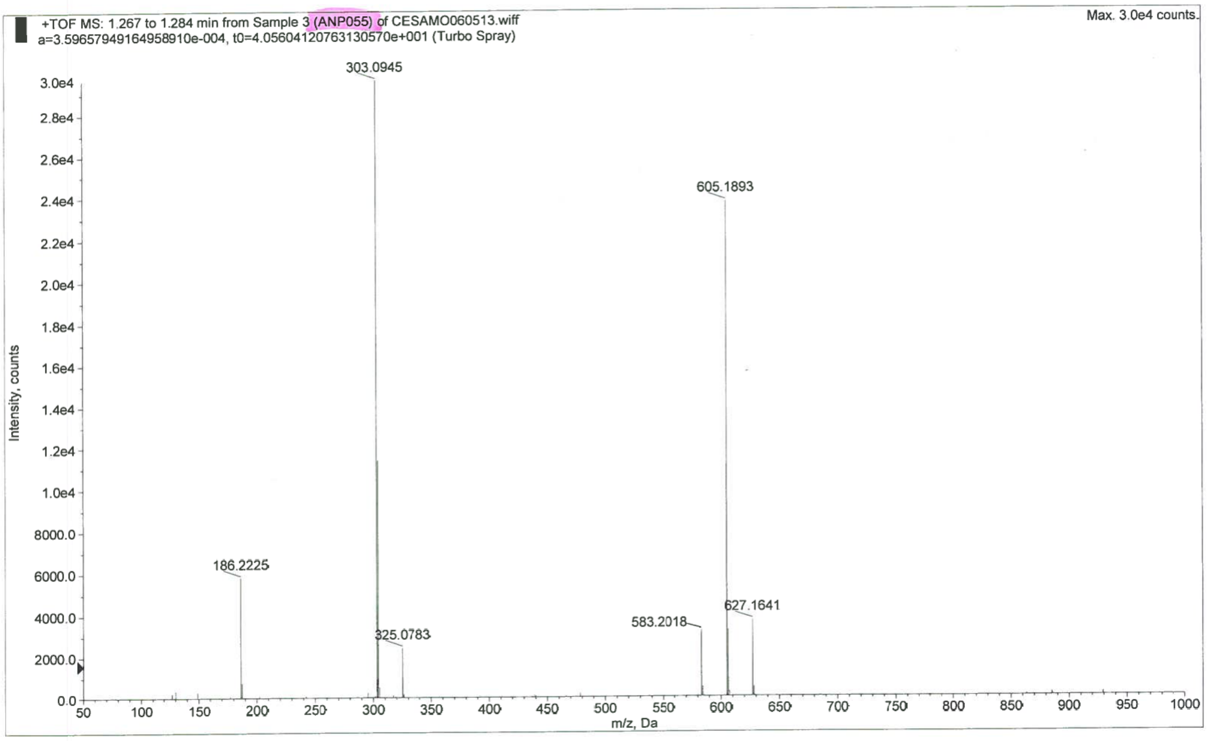

Mass spectrum of 1-((2*R*,4*S*,5*R*)-5-(hydroxymethyl)-4-(prop-2-ynyloxy)tetrahydrofuran-2-yl)-5-methylpyrimidine-2,4(1*H*,3*H*)-dione **13**

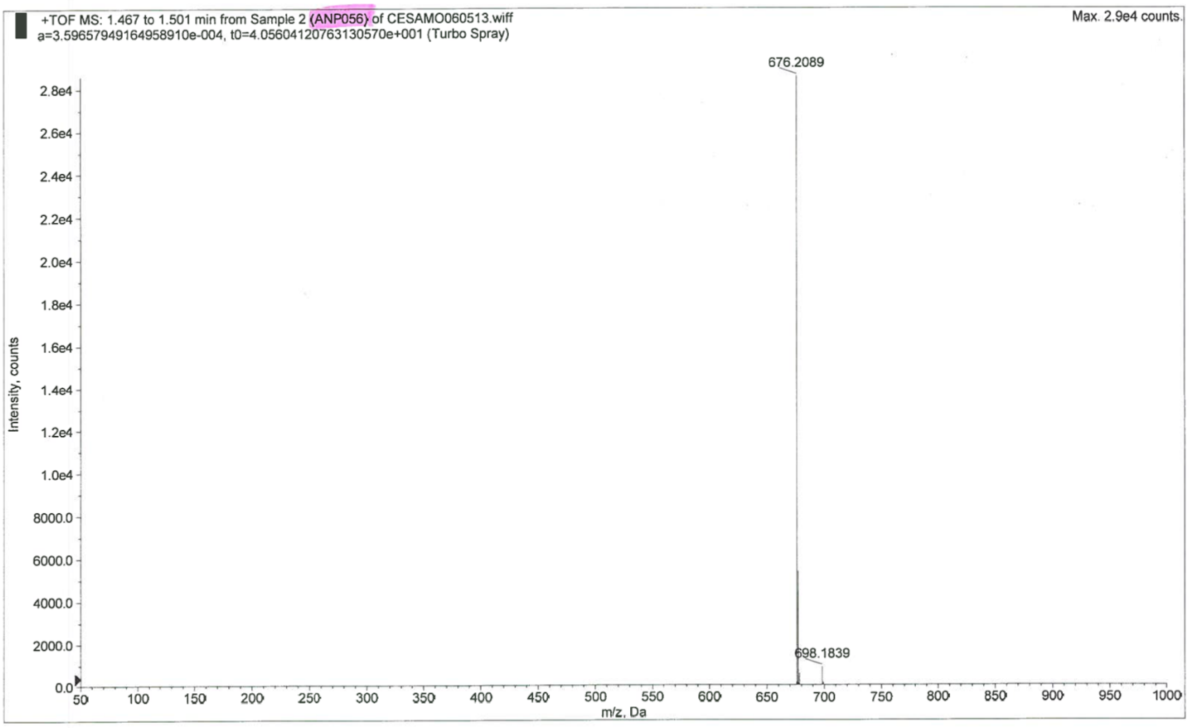

Mass spectrum of (2*R*,3*R*,4*S*,5*R*,6*R*)-2-(acetoxymethyl)-6-(4-(((2*R*,3*S*,5*R*)-2-(hydroxymethyl)-5-(5-methyl-2,4-dioxo-3,4-dihydropyrimidin-1(2H)-yl)tetrahydrofuran-3-yloxy)methyl)-1*H*-1,2,3-triazol-1-yl)tetrahydro-2*H*-pyran-3,4,5-triyl triacetate **14**

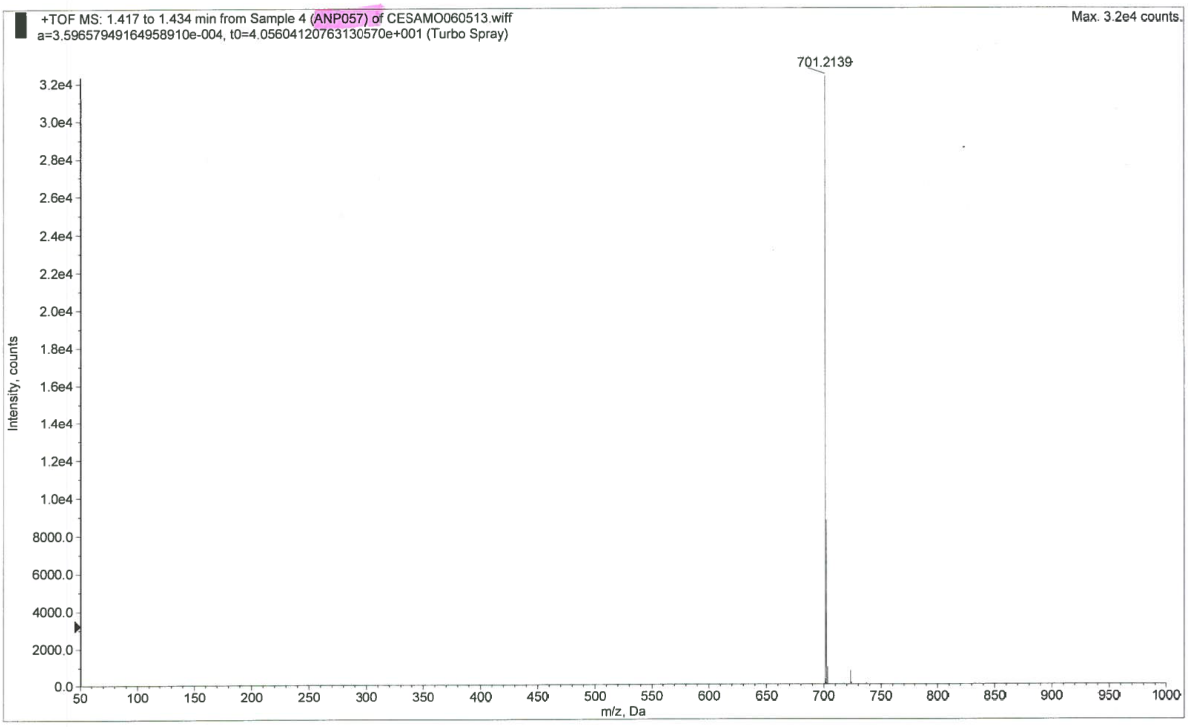

Mass spectrum of (2*R*,3*R*,4*S*,5*R*,6*R*)-2-(acetoxymethyl)-6-(4-(((2*R*,3*S*,5*R*)-2-(azidomethyl)-5-(5-methyl-2,4-dioxo-3,4-dihydropyrimidin-1(2*H*)-yl)tetrahydrofuran-3-yloxy)methyl)-1*H*-1,2,3-triazol-1-yl)tetrahydro-2*H*-pyran-3,4,5-triyl triacetate **15**

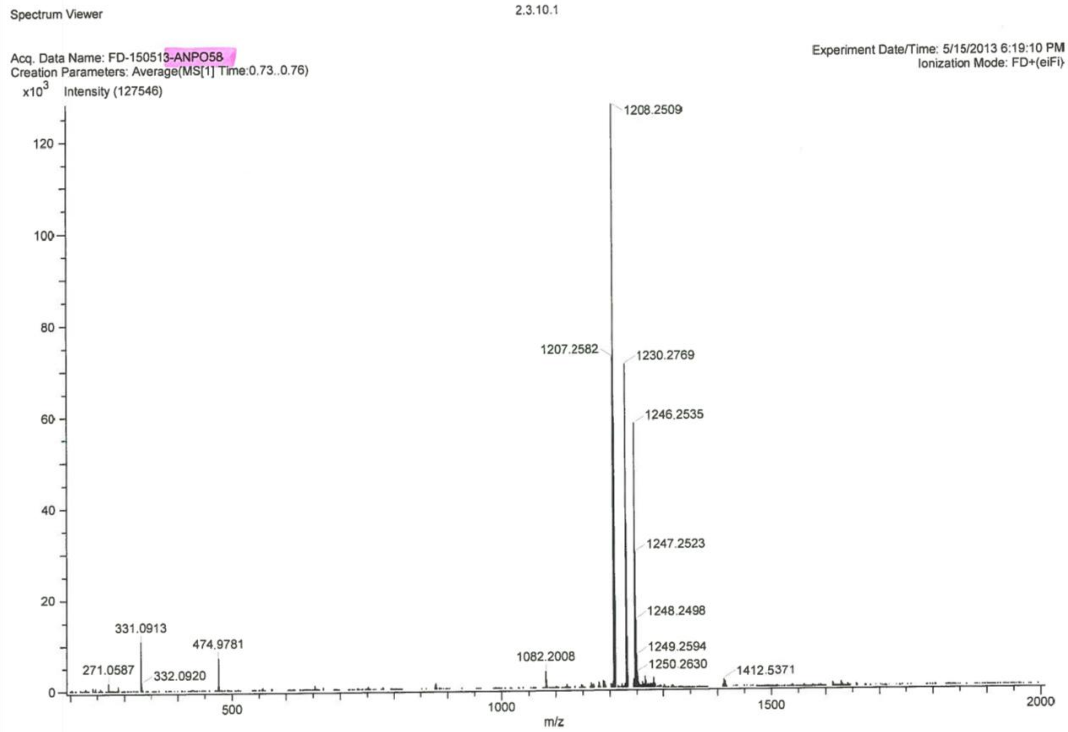

Mass spectrum of (2*R*,3*R*,4*S*,5*R*,6*R*)-2-(acetoxymethyl)-6-(4-(((2*R*,3*S*,5*R*)-2-((4-((4,4,5,5,6,6,7,7,8,8,9,9,10,10,11,11,11-heptadecafluoroundecanamido)methyl)-1*H*-1,2,3-triazol-1-yl)methyl)-5-(5-methyl-2,4-dioxo-3,4-dihydropyrimidin-1(2*H*)-yl)tetrahydrofuran-3-yloxy)methyl)-1*H*-1,2,3-triazol-1-yl)tetrahydro-2*H*-pyran-3,4,5-triyl triacetate **16**

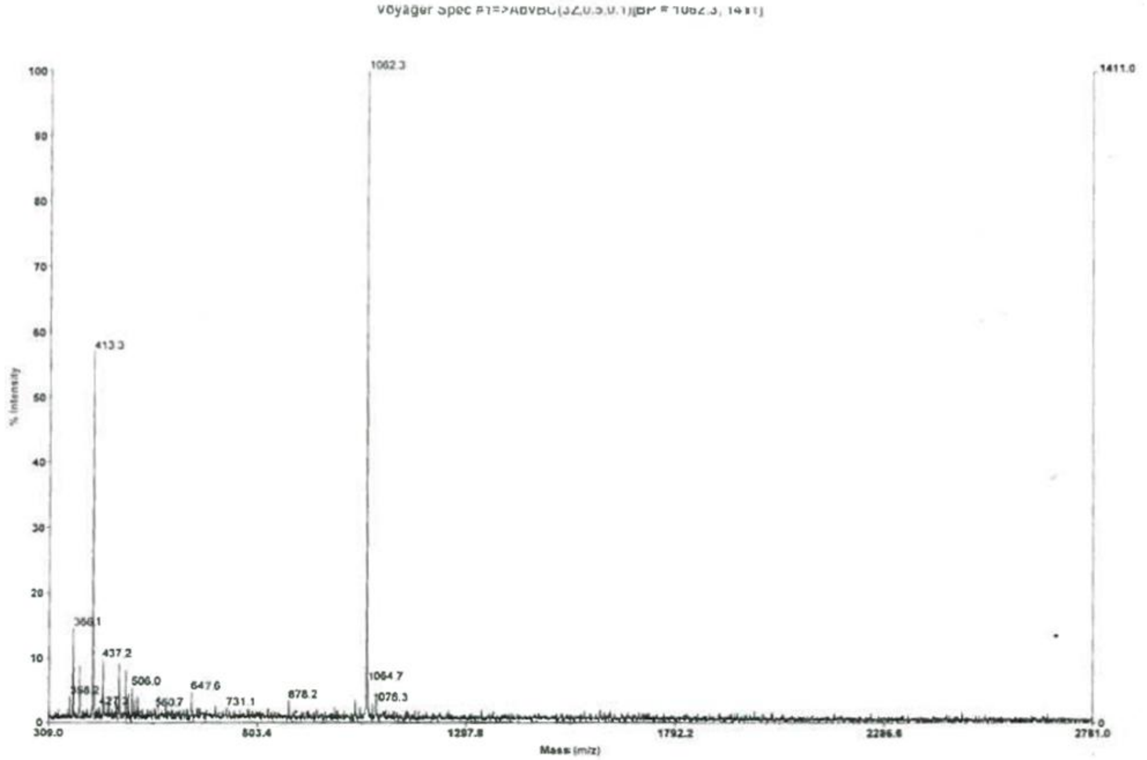

Mass spectrum of 4,4,5,5,6,6,7,7,8,8,9,9,10,10,11,11,11-heptadecafluoro-*N*-((1-(((2*R*,3*S*,5*R*)-5-(5-methyl-2,4-dioxo-3,4-dihydropyrimidin-1(2*H*)-yl)-3-((1-((2*R*,3*R*,4*S*,5*S*,6*R*)-3,4,5-trihydroxy-6-(hydroxymethyl)tetrahydro-2*H*-pyran-2-yl)-1*H*-1,2,3-triazol-4-yl)methoxy) tetrahydrofuran-2-yl)methyl)-1*H*-1,2,3-triazol-4-yl)methyl)undecanamide **17**
